# Supplementary material for: Data-driven insights to inform splice-altering variant assessment
Source: Am J Hum Genet. 2025 Mar 7;112(4):764–78. doi: 10.1016/j.ajhg.2025.02.012 (PMC12081236; doi:10.1016/j.ajhg.2025.02.012)
Supplement: Document S1. Figures S1–S4 [file mmc1.pdf]

The American Journal of Human Genetics, Volume *112*

Supplemental information

Data-driven insights to inform splice-altering variant assessment

Patricia J. Sullivan, Julian M.W. Quinn, Pamela Ajuyah, Mark Pinese, Ryan L. Davis, and Mark J. Cowley

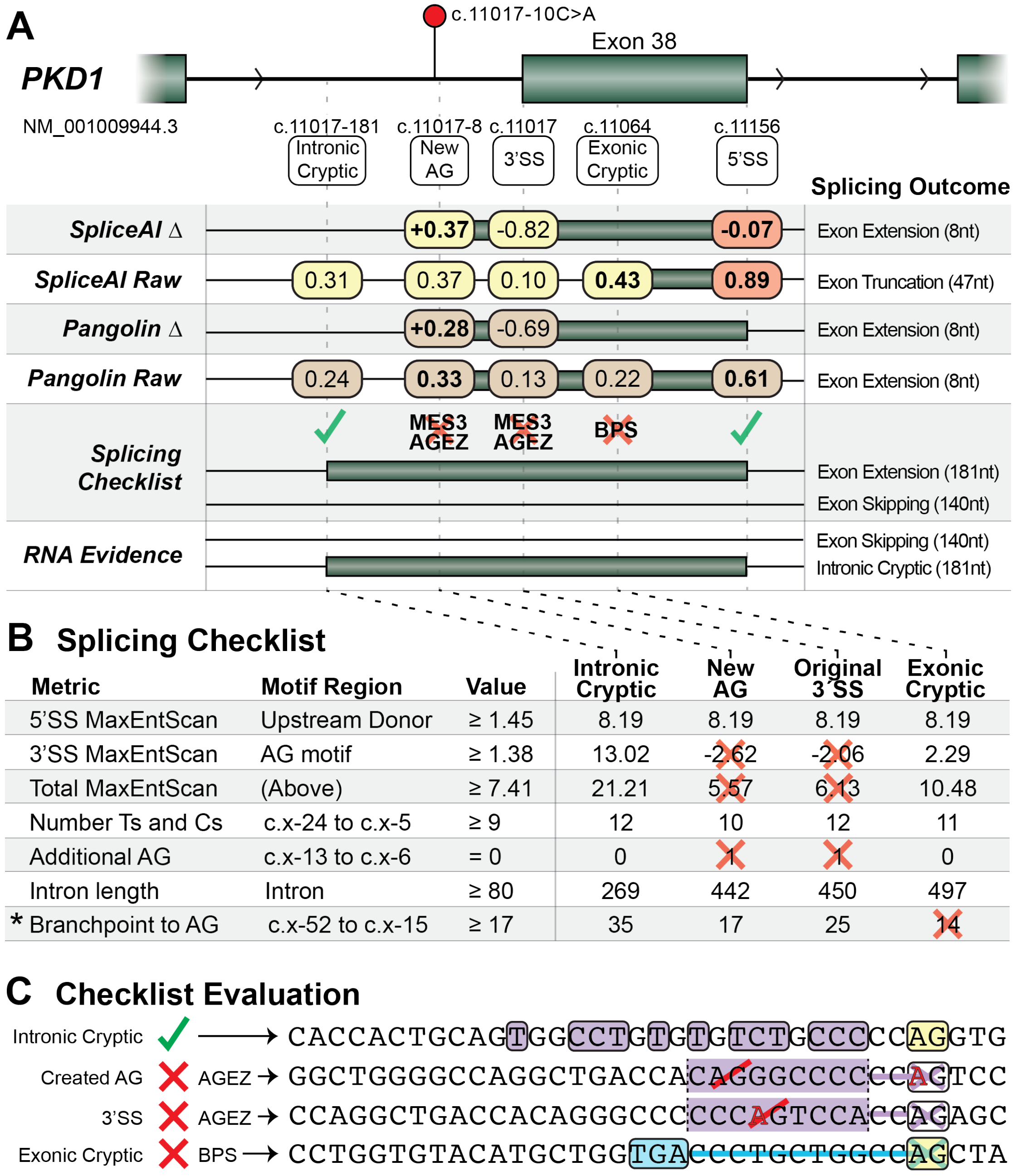

Figure S1: *In silico* prediction and heuristics comparison for a variant in *PKD1*. A) Lollipop plot of *PKD1* (NM_001009944.3) showing the transcript with the locations of the variants (not to scale). Dotted lines connect to the existing (3′SS and 5′SS) and proposed locations of splice sites, labeled with the exon’s first base (last base for 5′SS). Predictions are listed for the *PKD1*:c.11017-10C>A variant, using SpliceAI, Pangolin, our heuristics, and RNA Evidence (RT-PCR)[1]. Scores are color-coded based on the score class given (yellow: acceptor splice site; orange: donor splice site; brown: unspecified). The column on the right depicts the proposed splice site utilized as inferred from the scores. Δ: maximum score difference between the variant and wildtype. **B)** Heuristics-based interpretation of the proposed 3′SS locations. Values that do not meet the requirements are marked with a cross. **C)** Box colors correspond to the motif they represent (purple: polypyrimidine tract; yellow: acceptor motif; blue: branchpoint), white boxes at the canonical acceptor dinucleotides indicate the threshold for MaxEntScan 3′SS was not met. *: Not explicitly a requirement due to the difficulty of rule application.

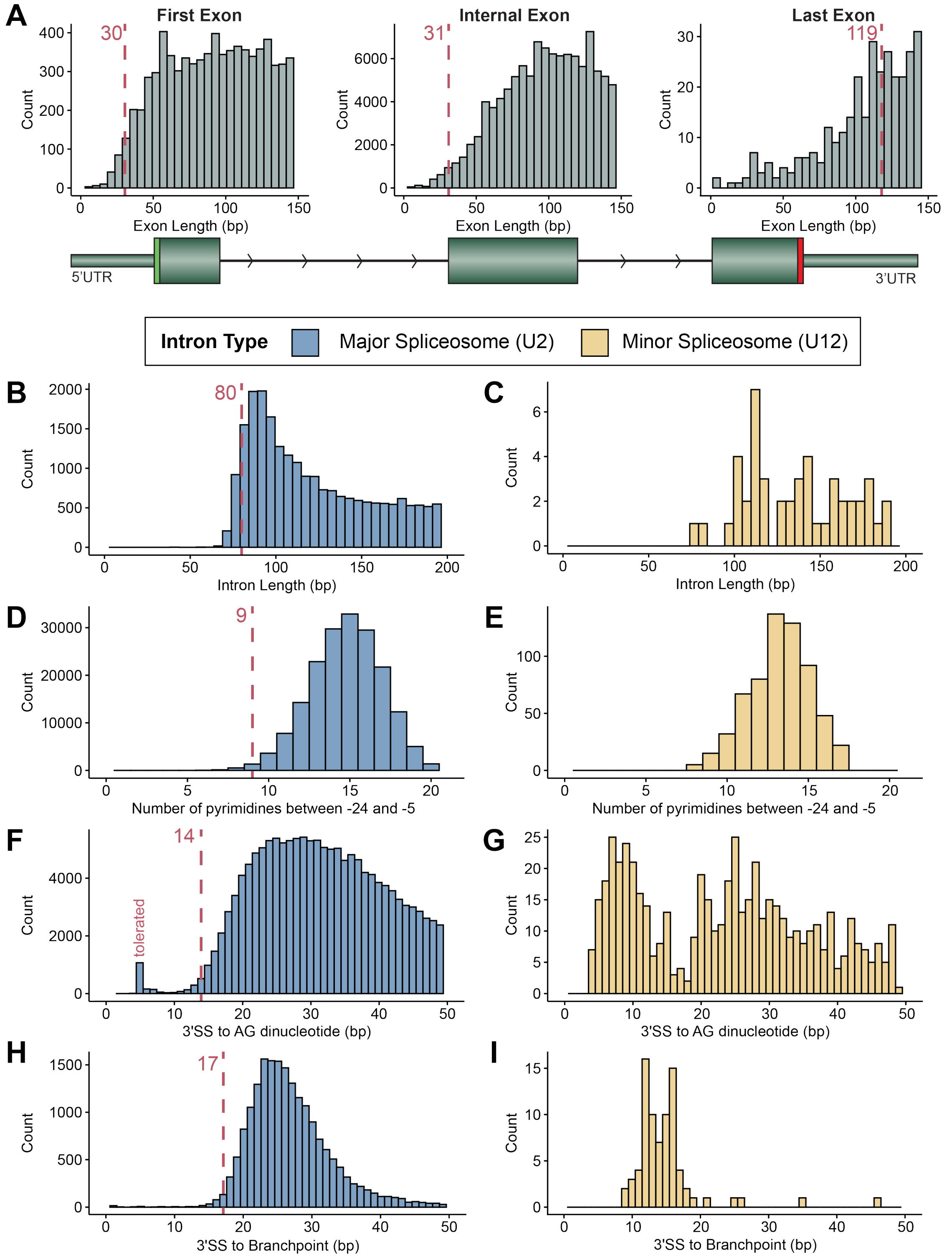

Figure S2: Characteristics of splicing features. Red lines show the value at which 99% of the U2 introns (left) are of equal value or greater. Characteristics are additionally shown for U12 introns (right) to show the difference in requirements for U2 and U12 introns. **A)** Minimum exon lengths for the first exon, internal exon (i.e., any exon not first or last), and last exon. **B-C)** Minimum intron lengths. **D-E)** Number of polypyrimidines (`T`s or `C`s) in the designated Polypyrimidine Tract Region (`−24` to `−5`). **F-G)** Location of the closest `AG` dinucleotide to the 3′SS. `AG` dinucleotides occurring at the `−4` location upstream of the 3′SS are tolerated. **H-I)** Positional requirement for the branchpoint motif in relation to the 3′SS. UTR: Untranslated Region. 3′SS: Acceptor Splice Site.

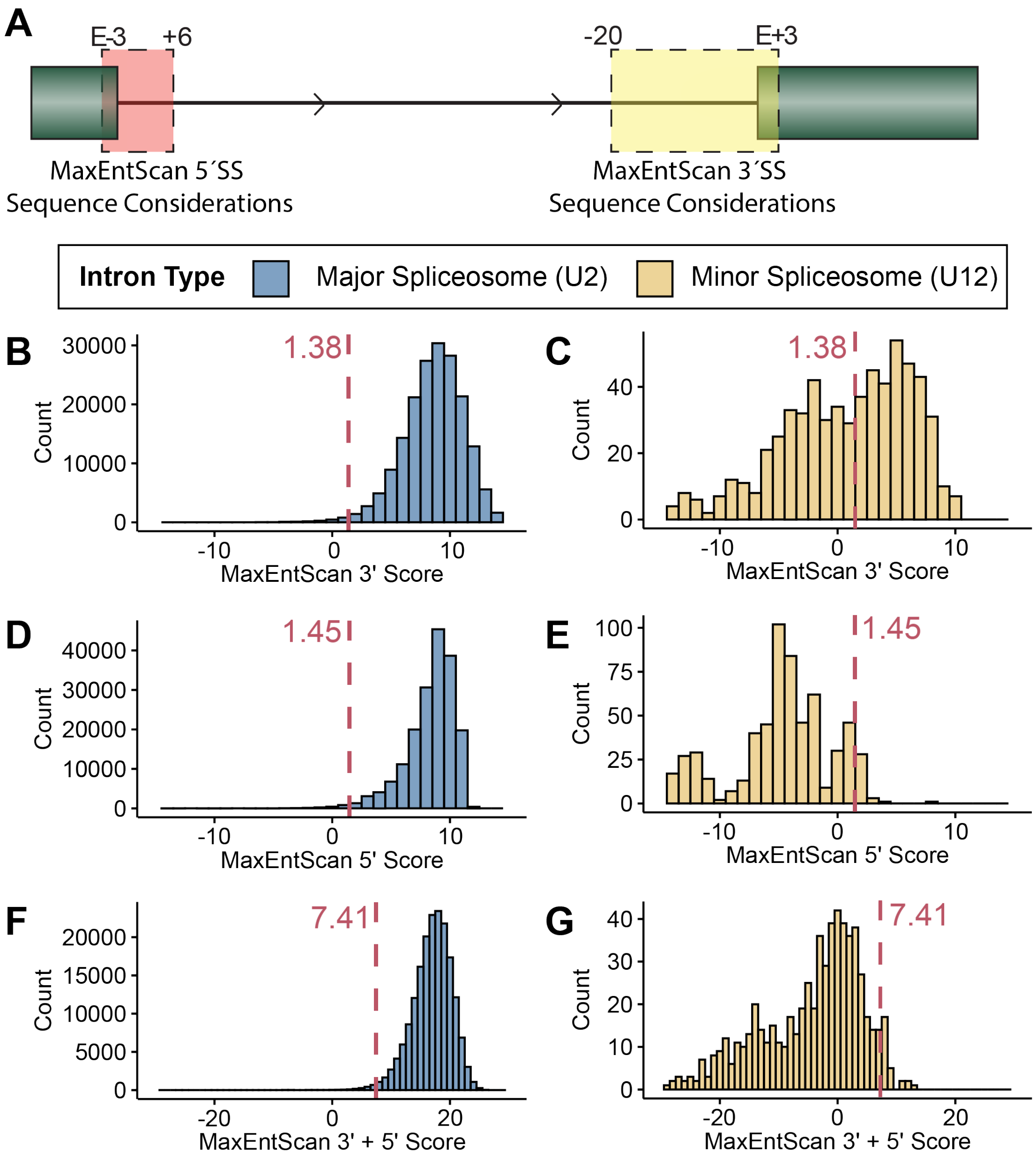

Figure S3: Splicing *In Silico* Score Distributions for the U2 Checklist. A) Green-filled boxes represent exons, and black lines with arrows represent the introns and the direction (5′ to 3′). The regions highlighted represent the sequence considered by MaxEntScan for 5′SS and 3′SS strength. **B-E)** Red lines show the value at which 99% of the U2 introns (left) are of equal value or greater. Score distributions are additionally shown for U12 introns (right) to show the limited applicability of the U2 requirements for U12 introns. **B-C)** MaxEntScan 3′ score distribution at the acceptor splice site. **D-E)** MaxEntScan 5′ score distribution at the donor splice site. **F-G)** MaxEntScan 3′ + 5′ score distribution at the upstream donor and acceptor splice sites (spanning the intron).

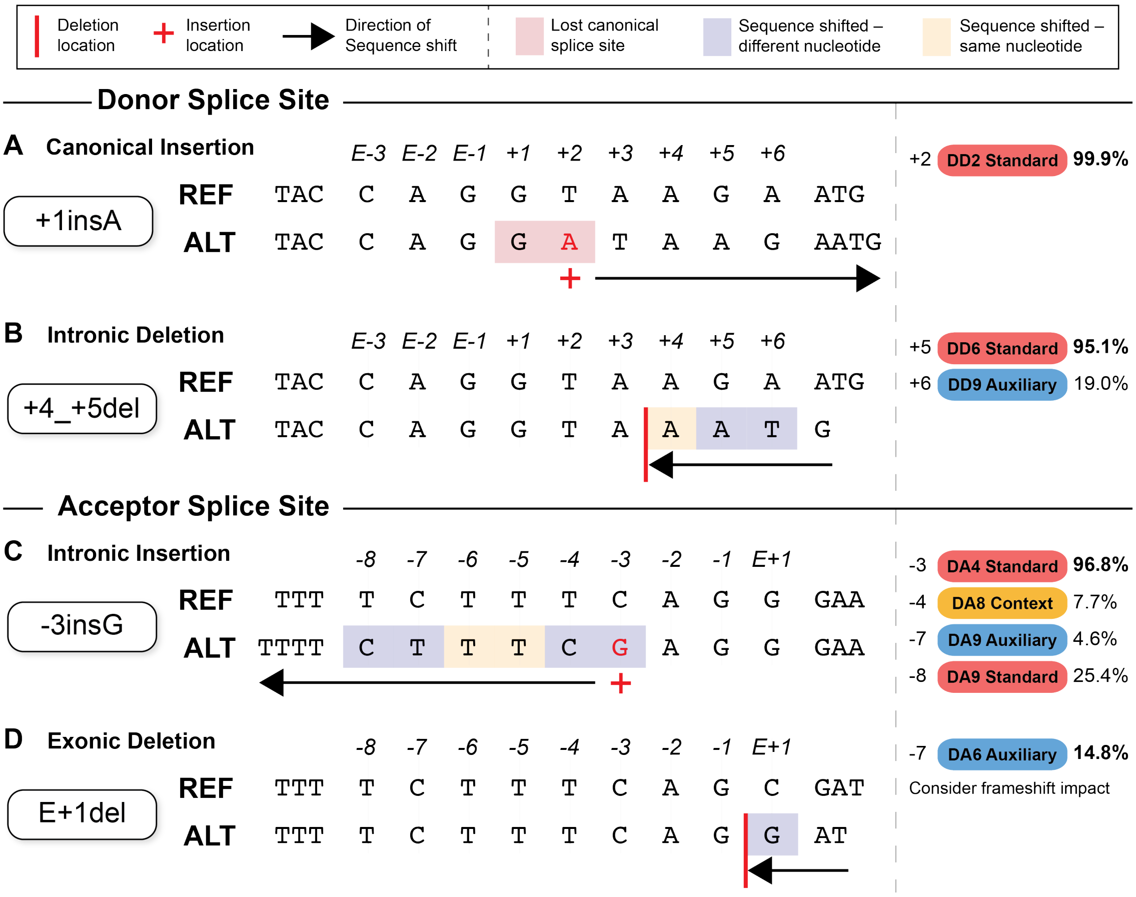

Figure S4: Application of the heuristics to small insertions and deletions. Splicing heuristics can be applied to small insertions and deletions by comparing the reference (ref) and variant (alt) sequences. The heuristics are applied to the nucleotides that differ between these sequences, with the highest spliceogenicity value assigned to the short insertion or deletion (indicated in bold).

References

[1] Hort, Y., Sullivan, P., Wedd, L., Fowles, L., Stevanovski, I., Deveson, I., Simons, C., Mallett, A., Patel, C., Furlong, T. *et al.* (2023). Atypical splicing variants in PKD1 explain most undiagnosed typical familial ADPKD. npj Genomic Medicine *8*. doi:10.1038/s41525-023-00362-z.
