# Supplementary material for: Data-driven insights to inform splice-altering variant assessment
Source: Am J Hum Genet. 2025 Mar 7;112(4):764–78. doi: 10.1016/j.ajhg.2025.02.012 (PMC12081236; doi:10.1016/j.ajhg.2025.02.012)
Supplement: Data S1. Handbook for data-driven splice-altering variant assessment [file mmc2.pdf]

Handbook for data-driven splice-altering variant assessment

Supplementary file for "*Data-driven insights to inform splice-altering variant assessment*"

This supplementary document accompanies the paper "Data-driven insights to inform splice-altering variant assessment." It is a stand-alone resource for researchers and variant curators seeking detailed insights beyond the core findings presented in the paper. It contains additional information not provided in the main paper, but as a standalone resource, it also includes necessary introductory material replicated from the main paper.

Contents

1 Terminology **3**

2 Splicing Requirements **5**

2.1 Methods Used to Determine the Splicing Requirements 6

2.2 Major Spliceosome (U2) Requirements 6

2.3 Major Spliceosome (U2) Checklist 9

2.4 Minor Spliceosome (U12) Requirements 11

2.5 Minor Spliceosome (U12) Checklist 12

3 Evaluating and Predicting Novel Splice Site Variants **15**

3.1 Case Study: Interpreting a Putative Novel Splice Site Variant, Within the AG-Exclusion Zone 15

3.2 Application of the Checklist for Pseudoexons 18

4 Splice Site Disruption Heuristics **20**

4.1 Disruption of the Donor (DD) Splice Site 22

DD1 Affecting a GC Donor Splice Site 22

DD2 Disrupting the canonical GT 24

DD3 Creating a TA at the third last base of the exon (E-3) 25

DD4 E-1 and +5 $\neq$ G, in combination 26

DD5 Creating a CC or GT dinucleotide at the +3 position 27

DD6 Affecting the +5 position 28

DD7 Affecting the last base of the exon (E-1) 29

DD8 Affecting the +3 position 31

DD9 Affecting the +6 position 33

DD10 Affecting the +4 position 34

DD11 Affecting the penultimate base of the exon (E-2) 35

DD12 Affecting the third last base of the exon (E-3) 37

4.2 Disruption of the Acceptor (DA) Splice Site 38

DA1 Creating an AG dinucleotide upstream of the canonical AG 38

DA2 Disrupting the canonical AG 41

DA3 First base of the exon (E+1) $\neq$ G and -3 $\neq$ C, in combination 41

DA4 Affecting the -3 position 43

DA5 Affecting the bases at intron -5 or -6 positions 44

DA6 Affecting the first base of an exon (E+1) 45

DA7 In the branchpoint region 47

DA8 Affecting the -4 position 49

DA9 In the Polypyrimidine tract region 50

Terminology

Acronyms

AGEZ	AG-Exclusion Zone.
Alt	The alternative nucleotide sequence introduced by a variant of interest.
BPS	Branchpoint Sequence.
DA	Heuristic pertaining to Disruption of the Acceptor splice site (3′SS).
DD	Heuristic pertaining to Disruption of the Donor splice site (5′SS).
E	Abbreviation used to denote an exonic base.
HGVS	Human Genome Variation Society.
PPT	Polypyrimidine tract.
Ref	The reference nucleotide sequence found in the reference genome.
SAV	Splice-Altering Variant.
SNV	Single Nucleotide Variant.
SRE	Splicing Regulatory Element.
SS	Splice site.
UTR	Untranslated region.

Definitions

Auxillary	Heuristic subgroup. Variants that are usually not splice-altering.
Contextual Modifier	Heuristic subgroup. The surrounding sequence context impacts the likely splicing outcome.
Spliceogenicity	Likelihood of a variant to alter splicing at its location or a nearby site. It does not indicate the directionality of the splicing event likelihood (i.e., whether increased or decreased) nor whether the change is pathogenic.
Standard	Heuristic subgroup. Variants that are usually splice-altering.

Splicing Outcomes

Exon extension	Part of an intronic sequence is included in the transcript.

Exon skipping	An entire exon is excluded from the transcript.
Exon truncation	Part of an exonic sequence is excluded from the transcript.
Intron retention	The entire intron is included in the transcript.
Pseudoexon inclusion	The inclusion of an intronic region in a transcript that does not adjoin or overlap any canonical exon and is not detected in mature transcripts.

Tools

MaxEntScan 3′SS	A maximum entropy model to measure 3′SS strength[1]. (http://hollywood.mit.edu/burgelab/maxent/Xmaxentscan_scoreseq_acc.html)
MaxEntScan 5′SS	A maximum entropy model to measure 5′SS strength[1]. (http://hollywood.mit.edu/burgelab/maxent/Xmaxentscan_scoreseq.html)
SpliceVarDB	A curated collection of experimentally investigated splice-altering variants from literature. Currently contains approximately 50,000 examples. (https://splicevardb.org)

Positional nomenclature

We adopted a splice variant nomenclature similar to the Human Genome Variation Society (HGVS) for reporting variant locations, relative to the closest exon/intron boundary. For intronic changes, we use '+' to mark positions at an intron's 5′ end and '−' for positions at its 3′ end. For changes within exons, 'E−' indicates positions at the exon's 3′ end, and 'E+' marks positions at the 5′ end. Thus, the first base of an exon is E+1, the penultimate base of an exon is E−2, and the third base of the intron following the 5′SS is +3.

Sequence Logos

Although splicing occurs at the RNA level, we use DNA sequences to represent the motifs, as most curators are attempting to interpret the effect of a putative SAV identified from DNA sequencing. DNA sequences are identical to the transcribed RNA sequences other than using T instead of U. All sequences and flowcharts in this document depicting introns and exons are oriented 5′ to the left and 3′ to the right.

Sequence motif logos throughout this study were created using *ggseqlogo*[2]. A sequence logo is created from a collection of aligned sequences to display the relative representation of nucleotides

at a given position, resulting in a consensus sequence. The height of a base (A, G, C, T) indicates the relative sequence conservation at that position (bits), with 2 bits representing an essential or required base.

Sequence logos for the 3′ and 5′ splice sites are created using protein-coding genes tagged with 'Ensembl canonical' sourced from Gencode v44. Sequence logos for the branchpoint were developed from experimentally validated splicing branchpoints collected from a large-scale experimental study (Mercer *et al.*[3], Supplementary Data 3).

Sequence logos included within a heuristic were created to show the conservation differences for motifs at the location of interest, with or without the sequence feature(s) of interest.

Splicing Requirements

We defined requisite splicing criteria by examining 183,000 canonical protein-coding exons and 19,000 experimentally-validated splicing branchpoints. This analysis determined the sequence constraint, relative spacing, and minimum strength of surrounding splice signals. These results were used to construct simple splicing requirements checklists that can be used to assess whether the spliceosome can recognize a candidate splice site.

The major (U2) and minor (U12) spliceosomes recognize different motifs for the branchpoint sequence, the acceptor splice site, and the donor splice site[4], resulting in different splicing prerequisites. The major spliceosome recognizes splice sites for the great majority of introns (∼99.5%); therefore, it is generally safe to consider only the U2 requirements. However, the splicing checklists do not apply well to introns spliced out by the minor spliceosome, with only 1.9% of U12 introns satisfying the U2 requirements and 2.7% of U2 introns satisfying the U12 requirements in return.

The requirements presented here were intentionally formulated to be inclusive in an attempt to summarize the conditions essential for splicing. Informed by splicing literature and evaluations that used known splicing motif locations, each checklist threshold was selected to capture nearly all protein-coding exons. When combined, 95.9% of U2 introns satisfy all of the U2 criteria, and 98.1% of U12 introns satisfy all of the U12 criteria.

Methods Used to Determine the Splicing Requirements

The splicing requirements were developed using 183,764 exons from protein-coding genes tagged with 'Ensembl canonical' sourced from Gencode v44. These exons also included 5′ and 3′ untranslated regions (UTRs), making the requirements applicable to the first and last exons as well.

U12 introns were identified using annotations from the Intron Annotation and Orthology Database[5]. Splice sites of these exons were separated into two tranches covering U2 introns (n=183,137) and U12 introns (n=627). We found that 0.3% of introns are of the U12 type, which is in line with the previously established proportion of ∼0.5% ref[4].

The splicing requirements related to the branchpoint were developed from 19,000 experimentally-validated splicing branchpoints collected from a large-scale experimental branchpoint study (Mercer *et al.*[3], Supplementary Data 3). Of the original 49,000 branchpoints, more than half of the exons (54.9%) in this dataset contain several potential branchpoint sites, while a minor portion (2.6%) have an annotated alternative 3′SS. To refine the analysis, we filtered the data to include only sequences featuring a single branchpoint and a singular annotated 3′SS within 50nt downstream, resulting in a focused dataset of 18,955 U2 and 79 U12 branchpoints for establishing our splicing requirements.

Major Spliceosome (U2) Requirements

Donor Splice Site (5′SS)

Recognition of the 5′SS by the U1 snRNP is one of the first steps in spliceosome complex formation[6]. Consistent with previous reports, the 5′SS consensus sequence demarcating an exon to be spliced by the major spliceosome was determined as `AG|GTRAG` (where `R` is purine; '`|`' is the exon-intron boundary with the exon underlined, throughout) (Figure 3A).

Acceptor Splice Site (3′SS)

Unlike the 5′SS, recognition of the 3′SS relies on splicing elements outside of the acceptor motif itself[6]. The recognition of multiple motifs results in specific positional constraints for the binding of these snRNPs[7], creating a "window" for each splicing motif (Figure 3B).

The constitutive motifs involved in 3′SS selection are the acceptor motif, the branchpoint motif, and the polypyrimidine tract (PPT). Consistent with previous reports, the 3′SS acceptor motif

consensus sequence demarcating an exon to be spliced by the major spliceosome was determined as YAG|G (where Y is pyrimidine) (Figure 5A).

Polypyrimidine Tract (PPT)

A stretch of pyrimidines (T and C nucleotides) of variable length upstream of the 3′SS forms the PPT. This expands the 3′SS consensus sequence to Y_nNYAG|G (where *n* is a variable number and N is any nucleotide) (Figure 3A). When considering a broad optimal PPT window between positions −24 and −5, the minimum number of pyrimidines required in that window was nine. While important for 3′SS recognition[8], we found that other PPT strength metrics, such as minimum uninterrupted polypyrimidines, thymine content, or maximum uninterrupted purines, were overly stringent to be applied as mandatory criteria.

Branchpoint

Upstream of the PPT, but within 17-50nt of the 3′SS, a branchpoint must be present. 72.3% of the filtered U2 branchpoints analyzed had position 0 located between −20 and −30 (inclusive). Consistent with previous reports, we determined a branchpoint consensus sequence of TNA. The A of the branchpoint is involved in the first splicing reaction (branching), to cleave the 5′ exon from the intron[6]. The A is largely invariable, occurring at position 0 in 91.8% of the filtered U2 branchpoints considered (Figure 5A)[3].

Although a branchpoint is essential for splicing, the canonical TNA motif was only found in 69.3% of branchpoints, indicating that this motif is too limited to be considered a defining sequence for the 3′SS requirement. By focusing on the introns that lacked a canonical TNA branchpoint motif, we identified four additional non-canonical branchpoint motifs that are not evident from the sequence logo (Table 1). The WNYA (where W is A or T and Y is C or T), CCN**A**, and CYNCC motifs were identified in 11.8%, 2.9% and 1.4% of branchpoints, respectively. Finally, 4.2% of the branchpoints contained a strong CTNA motif, offset by 1-2nt from the experimentally observed branchpoint, a previously described phenomenon[9].

Table 1: Motifs for branchpoint identification. The branchpoint locations were experimentally validated (Mercer *et al.*[3]) and filtered to include only sequences featuring a single branchpoint and a singular annotated 3′SS (See "Methods Used to Determine the Splicing Requirements"). The first section shows the sequence conservation of the A at position 0 of the branchpoint. Each was compared to the standard TNA motif to determine the value added by additional motifs.

Motif	Branchpoint Proportion	Branchpoint Proportion not captured by TNA
NNNAN	91.8%	22.5%
NTNAN	69.3%	-
WNYAN	27.9%	11.8%
CCNAN	2.9%	2.9%
CYNCC	1.4%	1.4%
CTNAN - shifted	4.2%	4.2%

The combination of these additional motifs resulted in 90.7% of filtered U2 branchpoints being identified.

AG-exclusion zone (AGEZ)

The 3′SS is sensitive to AG dinucleotides between the branchpoint and canonical splice site, a region termed the AG-Exclusion Zone (AGEZ)[10]. However, for 14% of exons, additional AG dinucleotides naturally occur between the branchpoint and 3′SS[11]. Nevertheless, we observed that AG dinucleotides were depleted between positions −13 and −6, but were tolerated at the −5 position (See Figure 1 for application). Toleration of some AG dinucleotides upstream of the 3′SS is due to the 3′SS AG-scanning recognition model, where the spliceosome loosely selects the first AG dinucleotide downstream of the branchpoint when lariat formation occurs[12]. Subsequently, a more precise identification of the canonical 3′SS occurs, allowing for a stronger AG to be used if within 5nt downstream of the first AG[13]. To complicate further the interpretation of the AGEZ, AG dinucleotides can be hidden by RNA secondary structure within stem-loops and thereby permit normal 3′SS recognition to proceed[12]. Our heuristics do not account for RNA secondary structures, like stem-loops, because these formations are uncommon and usually interfere with the recognition of the 3′SS[12,14]. A more detailed assessment of AGEZ variants by Zheng *et al.* recently conducted a genome-wide study of AG dinucleotides occurring in the 3′SS region[11].

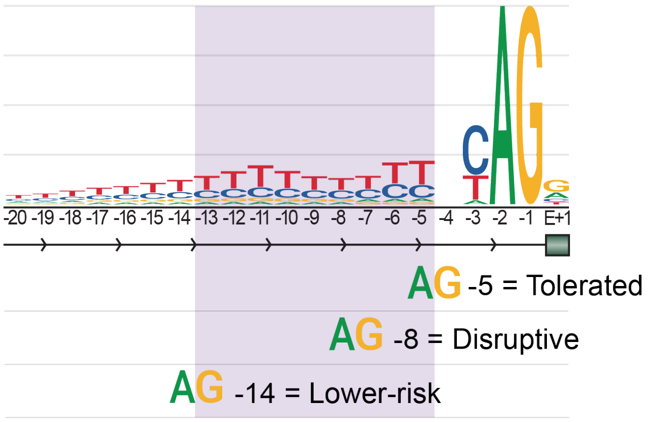

Figure 1: Toleration of AG dinucleotides preceding the 3′SS. AG dinucleotides located entirely within the purple shaded region thus disrupt the high-risk AGEZ zone and so are likely to interfere with 3′SS recognition at the 3′SS of interest. For plot terminology and construction, see Sequence Logos.

Major Spliceosome (U2) Checklist

To consolidate the above splicing requirements, we created a splicing checklist that can be used to determine the minimal requirements for whether an exon is likely to be included in a transcript (Figure 3C). We have found this checklist essential for interpreting the likely functional consequence of a given SAV as it enables the evaluation of potential cryptic splice sites to determine if they are usable (See "Evaluating and Predicting Novel Splice Site Variants"). This checklist serves as an initial, straightforward screening tool for assessing splice site viability. Below, we elaborate on heuristics for variants at established splice sites, where more rigorous criteria can be employed for analyzing a splicing motif already identified as recognizable.

We developed an *in silico* version of the checklist, by translating the qualitative assessments for both the 5′SS and 3′SS into specific thresholds for MaxEntScan, a widely used *in silico* tool for evaluating the strength of splice sites (Figures 3C and 2).

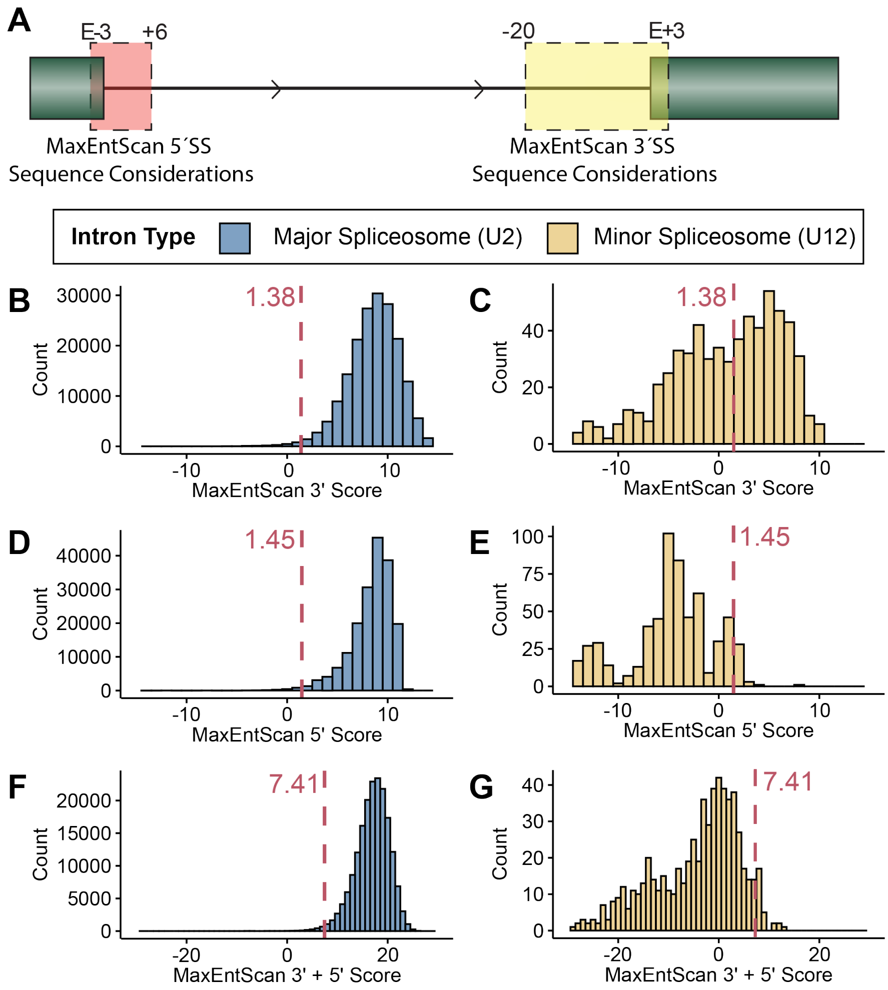

Figure 2: Splicing *in silico* score distributions for the U2 checklist. A) Green-filled boxes represent exons, and black lines with arrows represent the introns and the direction (5′ to 3′). Colored regions show the location of the sequence window used by MaxEntScan to score the relevant motif. **B-G)** Red lines show the value at which 99% of the U2 introns (left) are of equal value or greater. Score distributions are additionally shown for U12 introns (right) to show the limited applicability of the U2 requirements for U12 introns. **B-C)** MaxEntScan 3′ score distribution at the acceptor splice site. **D-E)** MaxEntScan 5′ score distribution at the donor splice site. **F-G)** MaxEntScan 3′ + 5′ score distribution at the upstream donor and acceptor splice sites (spanning the intron, as shown in A).

Although a branchpoint is essential for splicing, our checklists do not explicitly consider their identification. While utilizing the combined motifs presented in Table 1 identifies most of the experimentally determined branchpoint locations (90.7%), additional validation needs to be performed to assess the accuracy of the motifs returned. Additionally, branchpoint location can potentially be more accurately predicted using specialized *in silico* tools. However, even the most accurate among these, *Branchpointer*[15], has a low positive predictive value (PPV) of 30%, indicating a high rate of false positives[16].

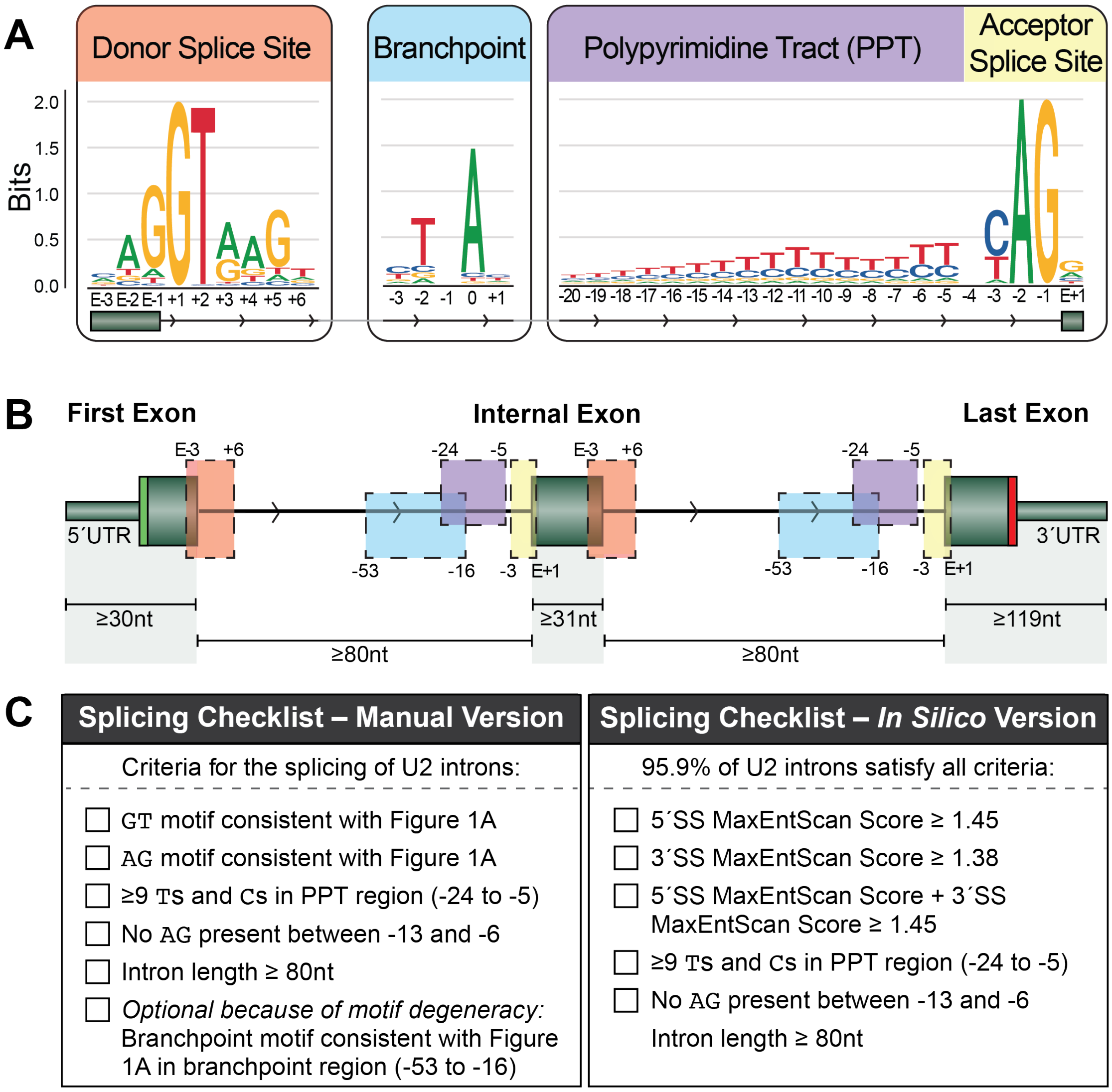

Figure 3: Splicing requirements for the major spliceosome (U2). A) Sequence logos of four main splicing motifs in protein-coding genes. For plot terminology and construction, see Sequence Logos. **B)** Relative location of splicing motifs, and length of exons and introns. Green-filled boxes represent exons, with the first, last, and internal exons displayed. Thinner sections of the first and last exons represent the untranslated regions (UTRs), demarcated by the start codon (light green) and stop codon (red). Black lines with arrows represent the introns and the direction (5′ to 3′). Colored boxes correspond to the locations of the motifs shown in **A**, with the flanking numbers demarcating their start and end locations relative to the nearest exon. The minimum intron and exon lengths are annotated below. **C)** Splicing checklists for manual and *in silico* determination of whether splicing will occur. `E+1`: First nucleotide of the exon. `E-3`: Third-last nucleotide of the exon. nt: nucleotide. PPT: Polypyrimidine Tract.

Minor Spliceosome (U12) Requirements

The consensus sequence for a minor spliceosome 5′SS is `|RTATCCT` (where `R` is a purine - either `A` or `G`). Unlike the 5′SS for the major spliceosome, the U12 5′SS motif is primarily determined by

the intronic base sequence. The consensus sequence for the minor spliceosome's acceptor motif is AS| (where S is either C or G), with no requirement for a PPT (Figure 5A).

There are two main combinations of the canonical acceptor and donor splice sites for the minor spliceosome: GT(5′SS)-AG(3′SS) and AT(5′SS)-AC(3′SS); other combinations of these motifs are unfavored [4]. The 3′SS for the U12 introns appears to be selected selected dependent on the branchpoint, with the consensus sequence of CCTTNA. The branchpoint for U12 introns does not follow the same distance requirements as the major spliceosome and is often as close as 9nt away from the 3′SS (Figure 5B).

Minor Spliceosome (U12) Checklist

U12 minor spliceosome motifs do not score favorably using general splicing tools. This is demonstrated by only 51% of U12 3′SS and 6% of U12 5′SS motifs scoring above MaxEntScan threshold (3′SS $\geq$ 1.38; 5′SS $\geq$ 1.45), where 99% of U2 splice sites exceed this threshold (Figure 2). Therefore, to accurately determine splicing prerequisites for the minor spliceosome, we developed position weight matrices (PWMs) for U12 splice sites and branchpoints (Figure 4).

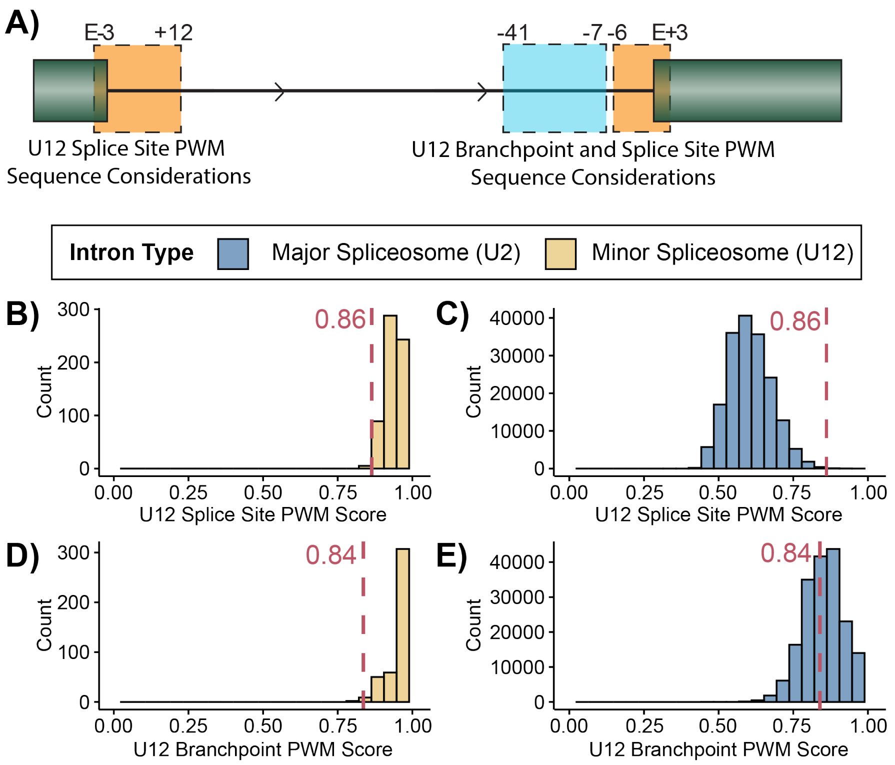

Figure 4: Splicing *in silico* score distributions for the U2 checklist. A) Green-filled boxes represent exons, and black lines with arrows represent the introns and the direction (5′ to 3′). **B-E)** Red lines show the value at which 99% of the U12 introns (left) are of equal value or greater. Score distributions are additionally shown for U2 introns (right) to show the limited applicability of the U12 requirements for U2 introns. **B-C)** Novel U12 PWM splice site scoring system for the upstream 5′SS and 3′SS (spanning the intron). **C-D)** Novel U12 PWM branchpoint sequence scoring system for the branchpoint region. PWM: Position Weight Matrix.

The branchpoint PWM scores the branchpoint alone, whereas the splice sites spanning either end of the intron are considered together as a strong preference for `GT`(5′SS)-`AG`(3′SS) and `AT`(5′SS)-`AC`(3′SS) is observed. These requirements determine the minimum splicing criteria for successfully identifying U12 introns, with 98.1% of U12 introns satisfying all of the U12 criteria (Figure 5B).

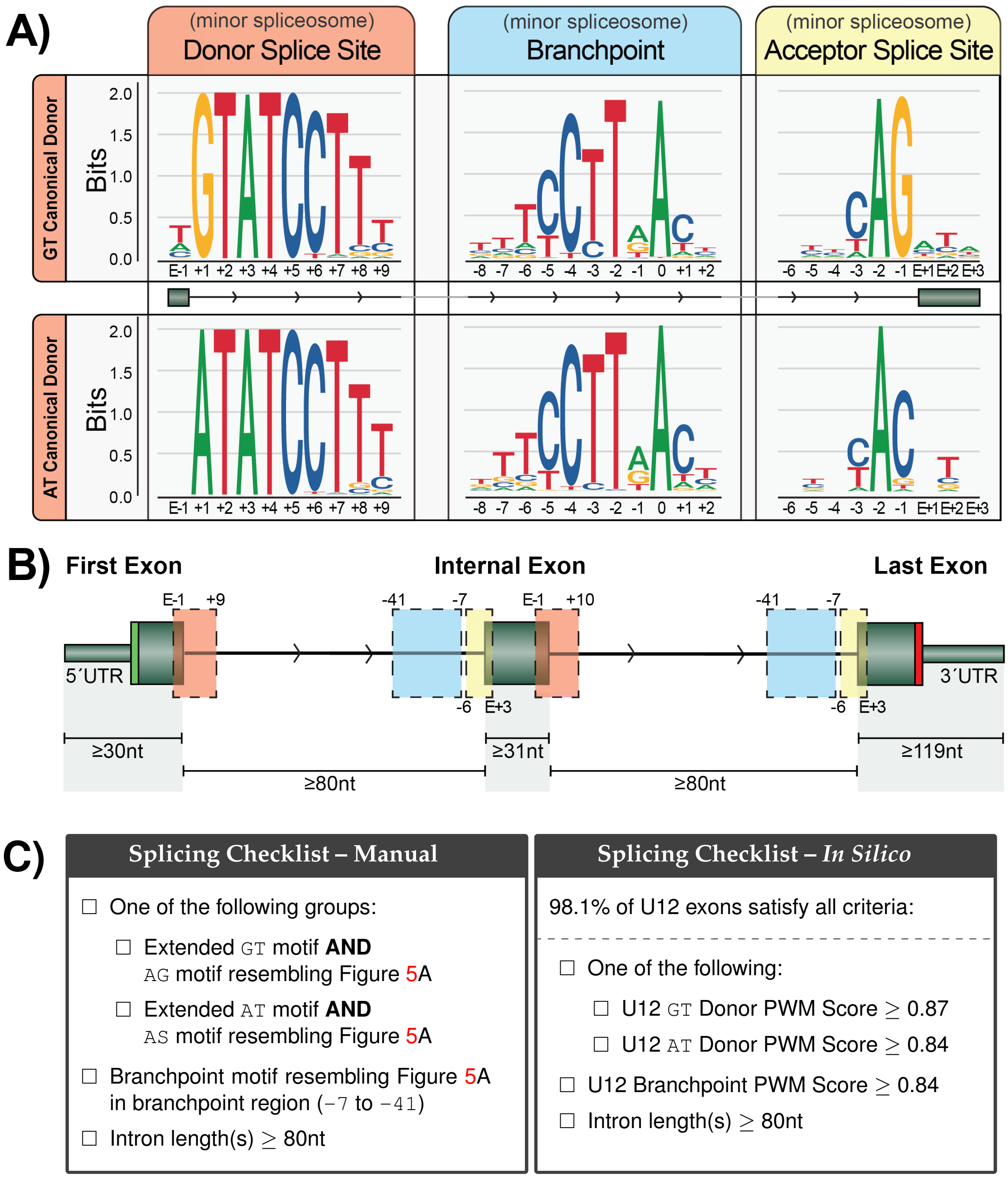

Figure 5: Splicing requirements for the minor spliceosome (U12). **A)** Sequence logos of four main splicing motifs in protein-coding genes, split into the two canonical donor site options: GT or AT. For plot terminology and construction, see Sequence Logos. **B)** Relative location of splicing motifs, and length of exons and introns. Green-filled boxes represent exons, with the first, last, and internal exons displayed. Thinner sections of the first and last exons represent the untranslated regions (UTRs), demarcated by the start codon (light green) and stop codon (red). Black lines with arrows represent the introns and the direction (5′ to 3′). Colored boxes correspond to the locations of the motifs shown in **A**, with the flanking numbers demarcating their start and end locations relative to the nearest exon. The minimum intron and exon lengths are annotated below. **C)** Splicing checklists for manual and *in silico* determination of whether splicing will occur (see "Minor Spliceosome (U12) Requirements"). `E+1`: First nucleotide of the exon. `E-3`: Third-last nucleotide of the exon. nt: nucleotide. PPT: Polypyrimidine Tract.

Evaluating and Predicting Novel Splice Site Variants

Identifying variants that create novel splice sites poses a greater challenge than assessing damage to existing ones because splicing requires sequences to appear in a precise order, with proper spacing, and with sufficient strength. The splicing heuristics help inform if a variant will disrupt existing splice sites, while the splicing checklists (See Figures 3 and 5) assist in assessing splice site functionality, for both new sites and those activating cryptic motifs.

Case Study: Interpreting a Putative Novel Splice Site Variant, Within the AG-Exclusion Zone

In this case study, we investigate the spliceogenicity of a rare, novel *PKD1*:c.11017-10C>A variant in a patient with suspected Autosomal Dominant Polycystic Kidney Disease. The variant created an `AG` dinucleotide near an existing 3′SS, and a cursory investigation with two leading *in silico* tools indicated that the variant would likely extend the exon by 8nt. However, applying our checklist revealed the correct cryptic splice sites and accurately predicted the functional impact[17].

Heuristic DA1 applies to variants in this region (as discussed below in "4.2"). As the `A` of the `AG` falls within `-13` to `-6`, the DA1 Standard category applies, which indicates that this variant is almost certain to affect splicing (Spliceogenicity of 99.7%, 95% CI [92.9,100], n=304). Furthermore, the most likely functional consequence is exon extension (86% likelihood), so the challenge is to identify the correct 3′SS, and for this, we use the splicing checklist.

Natural 3′SS Disruption and New AG Viability

Both SpliceAI and Pangolin predicted that the *PKD1*:c.11017-10C>A variant would create a novel 3′SS (+0.37 or +0.28, respectively) and that the natural 3′SS (Figure 6A "3′SS ") would no longer be recognized (-0.82 and -0.69, respectively). This would lead to an 8nt exon extension (Figure 6 "SpliceAI Δ" and "Pangolin Δ"), causing a frameshift.

However, when we apply our checklist, several aspects argue against this outcome. The novel `AG` had an existing `AG` dinucleotide within its AGEZ (at `-20`, 12nt upstream of the novel `AG`'s potential 3′SS), and the strength of the novel 3′SS and the combined 5′SS +3′SS fell below our thresholds. We scanned the surrounding sequence and identified two alternative `AG` motifs, which could act as cryptic splice sites, so here we apply the checklist to determine which is most likely to be utilized.

Exonic Cryptic 3′SS Viability

An exonic cryptic splice site (*PKD1*:c.11064) was the next strongest alternative 3′SS as predicted by SpliceAI's raw score (0.43). When applying the splicing checklist (Figure 6 "Exonic Cryptic"), this variant satisfied all but one of these requirements. We have highlighted the difficulties locating branchpoints, but if one can be located, it must be 17-50nt upstream of the 3′SS. Here, we identified a canonical `TNA` branchpoint motif just 14nt upstream of the putative cryptic 3′SS, rendering this cryptic 3′SS unusable.

Intronic Cryptic 3′SS Viability

The final remaining candidate cryptic splice site was intronic (*PKD1*:c.11017-181), and was not identified by either SpliceAI or Pangolin's delta scores, though their raw scores suggested this site could be a viable 3′SS. When applying the splicing checklist (Figure 6 "Intronic Cryptic"), this variant satisfied all splicing requirements defined by the checklist, including a very strongly scoring motif (by MaxEntScan), and a strong PPT (12 pyrimidines). Additionally, a potential canonical branchpoint was identified 35nt upstream of this cryptic splice site, within the required range of 17-50nt. Therefore, applying the splicing checklist determined that this intronic cryptic splice site was the most likely 3′SS.

Splicing Outcome Evaluation of the Putative Novel Splice Site Variant

An analysis of the potential 3′SS candidates surrounding *PKD1* exon 38 using the checklist (Figure 6B&C) revealed that only the intronic cryptic splice site was viable. This intronic cryptic 3′SS is 181nt upstream of the original; however, 93.3% of intronic cryptic splice sites in SpliceVarDB are within 100nt of the natural 3′SS. Thus, there is the potential that this cryptic splice site, although strong, will not be favored.

If cryptic splice sites are unavailable, the likelihood of exon skipping or intron retention must be analyzed. Therefore, as exon skipping was more commonly associated with AGEZ variants than intron retention (Heuristic DA1), we expect to observe some degree of exon skipping combined with exon extension.

This variant was experimentally validated following the application of the heuristics and revealed a combination of exon skipping and exon extension involving the intronic cryptic splice site, as predicted[17].

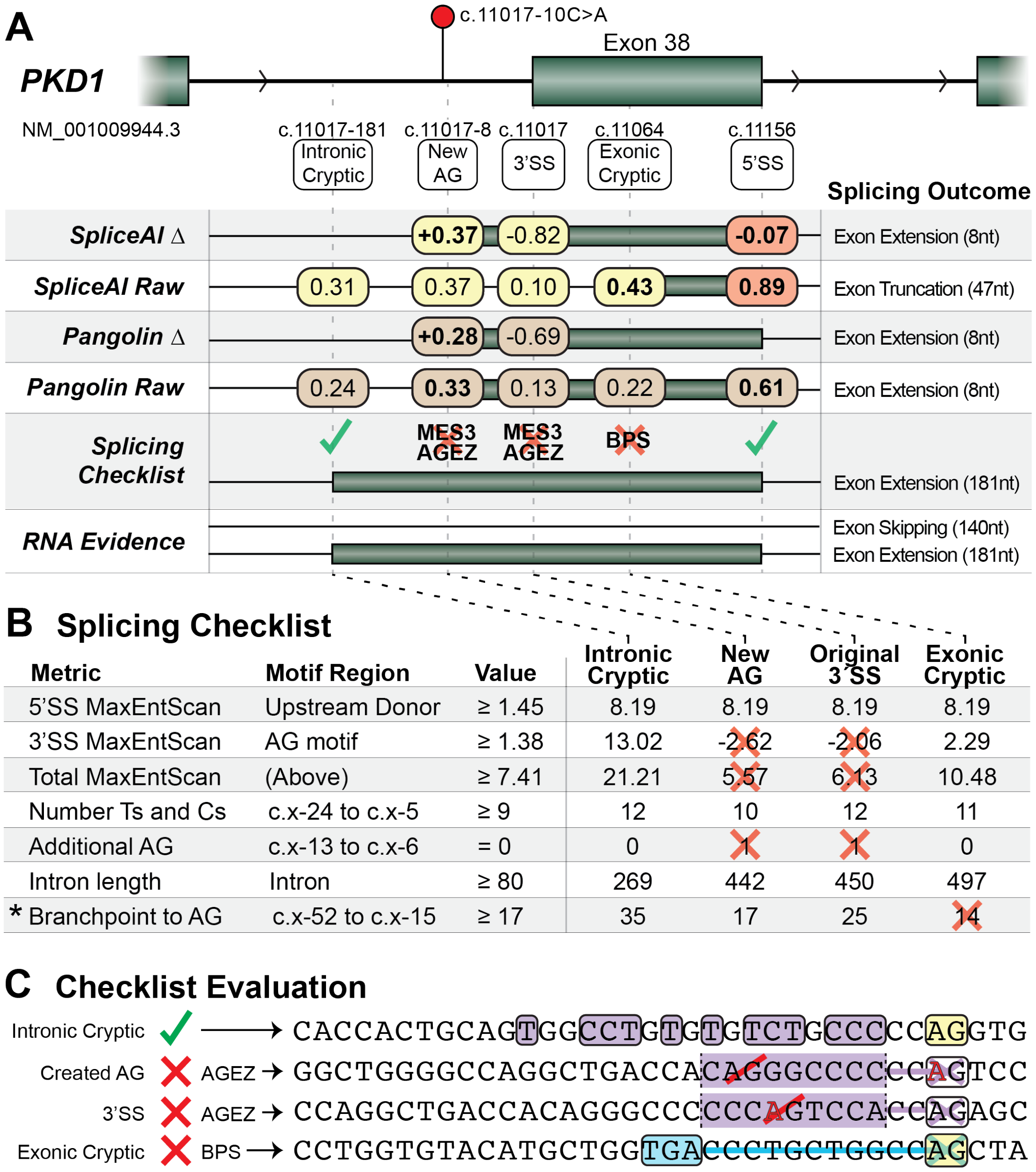

Figure 6: ***In silico*** **prediction and heuristics comparison for a variant in** ***PKD1*****.** **A)** Lollipop plot of *PKD1* (NM_001009944.3) showing the transcript with the locations of the variants (not to scale). Dotted lines connect to the existing (3′SS and 5′SS) and proposed locations of splice sites, labeled with the exon's first base (last base for 5′SS). Predictions are listed for the *PKD1*:c.11017-10C>A variant, using SpliceAI, Pangolin, our heuristics, and RNA Evidence (RT-PCR)[17]. Scores are color-coded based on the score class given (yellow: acceptor splice site; orange: donor splice site; brown: unspecified). The column on the right depicts the proposed splice site utilized as inferred from the scores. Δ: maximum score difference between the variant and wildtype. **B)** Heuristics-based interpretation of the proposed 3′SS locations. Values that do not meet the requirements are marked with a cross. **C)** Box colors correspond to the motif they represent (purple: polypyrimidine tract; yellow: acceptor motif; blue: branchpoint), white boxes at the canonical acceptor dinucleotides indicate the threshold for MaxEntScan 3′SS was not met. *: Not explicitly a requirement due to the difficulty of rule application.

Application of the Checklist for Pseudoexons

A pseudoexon, or a poison exon, is normally an intronic segment that becomes recognized by the spliceosome and included in the mRNA as an exon due to a 'deep intronic' SAV[18]. The alteration of just one base can be enough to result in pseudoexon inclusion, implying that most of the sequence elements required for an exon to be recognized are already present, albeit with suboptimal motif strength to have been recognized. Pseudoexons are predominantly deleterious as they often introduce a premature termination codon to the transcript through a frameshift or the inclusion of a stop codon in the newly included intronic sequence.

Variants from SpliceVarDB that were denoted as creating a pseudoexon were used to determine if the minimal splicing requirements could be used to identify the mechanism of pseudoexon inclusion. For the variants where a theorized mechanism of pseudoexon creation was identified, we once again utilized the minimal splicing requirements to identify potential partner splice sites. If the variant activated a 3′SS, we assessed all `GT` dinucleotides in the reference sequence, within a window of 31-750nt (corresponding to the minimum exon length vs a practical upper limit) against the splicing checklist (see Figure 3C) to determine if any met the minimal splicing requirements.

In SpliceVarDB, 167 deep intronic variants were explicitly reported by their original publications to create a pseudoexon. For these variants, the apparent predominant mechanisms for pseudoexon inclusion involved the creation of a canonical 5′SS (n=61), which occurs more commonly than the creation of a canonical 3′SS (n=23) (Figure 7). Alternatively, 44 variants occurring at a non-canonical base led to the inclusion of a pseudoexon by strengthening a pre-existing, yet unutilized, splice site (Figure 7 "Candidate site in Ref & Alt"). While these cryptic splice sites already met the *in silico* criteria for splicing to occur (Figure 3C), their inclusion in a transcript was dependent on the inclusion of the observed variant. The remaining non-canonical variants with a theorized pseudoexon inclusion mechanism satisfied the previously unmet splicing requirements, enabling the pre-existing `AG` or `GT` dinucleotide to be recognized as a splice site (Figure 7 "Candidate site only in Alt"). The remaining variants (n=17) lacked a suggested mechanism for their involvement in pseudoexon inclusion (Figure 7 "No Candidate Sites in Alt").

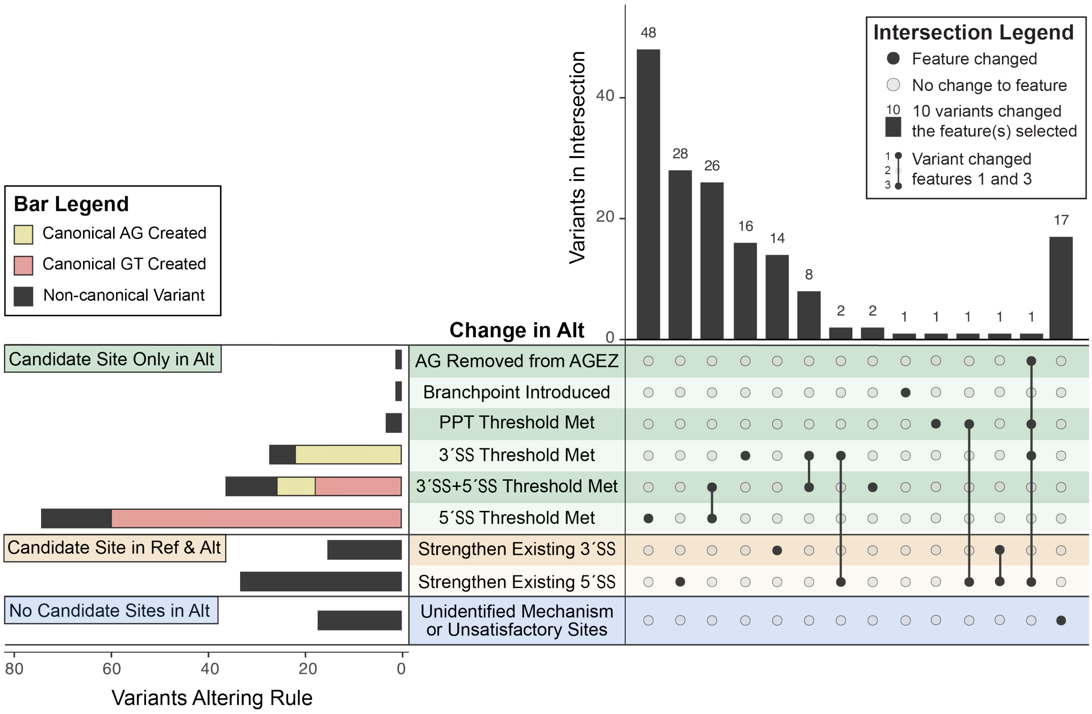

Figure 7: Theoretical reconciliation of pseudoexon inclusion mechanisms. UpSet[19] plot of the alterations made by deep intronic variants that promote the inclusion of pseudoexons, as determined by applying the splicing heuristics to the reference (Ref) and alternative (Alt) sequences. Alterations are divided into variants that were deemed by the splicing requirements (see "Splicing Requirements") to create a usable splice site not present in the reference sequence (green), variants that strengthened a cryptic splice site that pre-existed in the reference sequence (orange), and variants where the mechanism of pseudoexon alteration could not be determined using the requirements (blue). The number of variants that matched each feature change is shown in the left horizontal bar plot, where variants are colored according to whether the variant created a canonical `AG` in a candidate 3′SS (yellow), a canonical `GT` in a candidate 5′SS (red), or altered a splicing motif at a non-canonical nucleotide (black).

Predicting whether a deep intronic variant creates a pseudoexon involves finding nearby cryptic splice sites that could pair with it. By applying our splicing criteria (Figure 3C) to the sequence around the variant, we located on average three suitable cryptic splice sites for each variant capable of generating a pseudoexon, including 82% of those confirmed by experimental evidence.

We further investigated the 17 variants that led to the formation of pseudoexons without a clear mechanism fitting the splicing criteria (Figure 7 "No Candidate Sites in Alt"). Associated literature for 65% (n=11) of the variants reported creation or disruption of a splicing enhancer and/or silencer motif[20–29]. For the remaining six variants, we further investigated why they were not identified using the splicing requirements. We found that three promoted the use of unsatisfactory splice sites, two did not sufficiently alter the splice site strength to be classified as "strengthening", and one was considered to be outside the impact range for the nearby 5′SS. These mechanisms could explain the pseudoexons' presence, though an uninvestigated impact on splicing

regulation by the original study remains a possibility.

Splice Site Disruption Heuristics

This section presents a comprehensive set of heuristics for evaluating putative SAVs, their potential to disrupt splicing, and their likely impact. These heuristics have been developed using aggregated observations from 11,860 high-confidence, experimentally-validated variants from SpliceVarDB: 75.2% of which did, and 24.8% of which did not alter splicing. At least 10 functionally validated variants informed each heuristic to establish reliability for determining splicing outcome(s) and avoid overfitting.

These heuristics are specifically for assessing variants that disrupt the 5′SS (DD) or the 3′SS (DA). The creation of novel splice sites can be assessed by applying the "Splicing Requirements". A detailed section on the practical application of these requirements, including examples, is provided at "Evaluating and Predicting Novel Splice Site Variants".

There were too few examples of experimentally confirmed SAVs that affect splicing regulatory elements (SREs) or U12 introns to make specific heuristics for these cases.

The core of each disruption heuristic starts with determining the variant location relative to an annotated splice site (see Figure 8A). The location of the variant determines which heuristic(s) apply, such as DD4 and DD6 for a variant at the +5 position of the donor splice site (Figure 8A). Within each heuristic, variants are assigned to subgroups intended to capture variants with similar impacts on splicing and, consequently, similar spliceogenicity. Note that subgroups are named based on the relevant information used for grouping, rather than their spliceogenicity. All heuristics have a default 'Standard' subgroup, designed to capture the SAVs impacting that location. However, splicing motifs are not recognized in isolation, and additional factors can affect the spliceogenicity of a variant. A 'Contextual Modifier' subgroup was included to account for the influence of nearby variant-independent sequence context features on spliceogenicity. The 'Auxiliary' subgroup serves as a catch-all for variants not included in either subgroup, primarily representing variants less likely to be splice-altering. We endeavored only to add contextual modifiers if their inclusion led to a spliceogenicity separation of at least 15% between subgroups.

We provide a panel showing the decision flow diagram for all of the heuristics, indicating whether the 'Standard', 'Context', or 'Auxiliary' subgroup applies. For each subgroup, a spliceogenicity parameter was calculated to provide a measure of accuracy for curators and researchers wishing

to apply these heuristics. Spliceogenicity gives the proportion (%) of variants in the subgroup found to be splice-altering; the 95% confidence intervals for spliceogenicity are also provided in square brackets. We also indicate the number (n) of applicable genetic variants examined for each subgroup. We provide data on the proportion of variants that result in exon skipping, exon truncation, exon elongation, and intron retention. Regardless of their subgroup, the proportions indicated pertain to all SAVs in a heuristic. SAVs following the 'Context' and 'Auxiliary' flowcharts resulted in similar proportions of splicing outcomes, i.e., their effect on splicing differed from those of 'Standard' subgroup variants only in spliceogenicity.

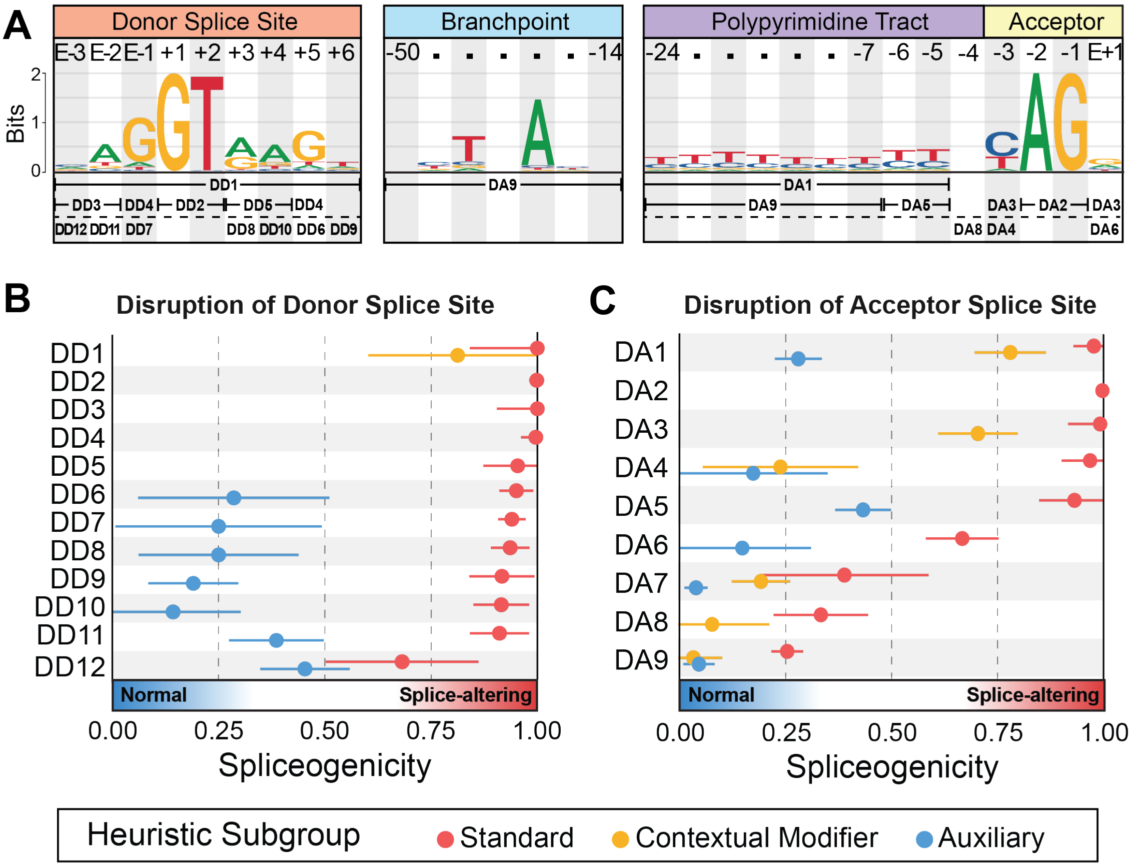

Figure 8: Overview of splicing heuristics for donor and acceptor splice site disruption. A) Sequence logos for splicing motifs at the donor splice site, branchpoint, polypyrimidine tract, and acceptor splice site. These logos represent the consensus sequences and relative frequencies of nucleotides at each position within the splicing motifs. The relevant splicing heuristics (outlined in B) and C)) are indicated below their corresponding nucleotide location. **B-C)** Spliceogenicity (likelihood of a splice-altering event occurring) metrics of heuristics for variants that B) disrupt the donor splice site (DD; 5′SS) and C) disrupt the acceptor splice site (DA; 3′SS). The dots on the horizontal lines reflect the spliceogenicity of each subgroup, while the extent of the line denotes the 95% confidence interval, illustrating the precision of the spliceogenicity estimate.

Disruption of the Donor (DD) Splice Site

Splice site selection at the 5′SS primarily depends on the donor motif. Of the 8,920 SAVs identified, 4,786 variants impacted the 5′SS. Applying the splicing requirements checklist (Figure 3C) to variants affecting the 5′SS revealed that 46% (n=2,205) render the original splice site unusable. A further 53% (n=2,542) weaken the original motif, leaving it functional but less likely to be recognized by the spliceosome. This underscores the significance of motif strength in splice site selection.

The DD heuristics are presented below, first addressing variants that affect 5′SS with `GT` as the canonical dinucleotides. They follow the numbering and splicing outcome diagram conventions used in Figures 3 and 6 of the main paper (Sullivan *et al.*).

Heuristic DD1

For variants: Affecting a GC Donor Splice Site

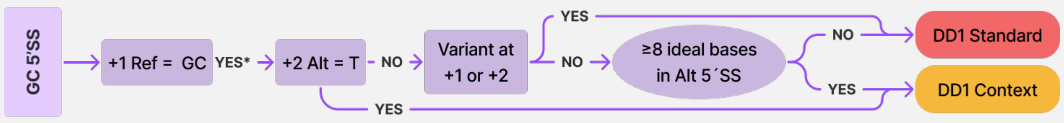

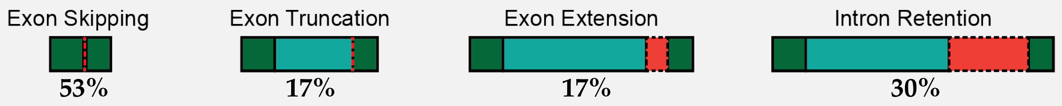

Standard Subgroup	Spliceogenicity=100% [84.1, 100]	n=28
Contextual Modifier	Spliceogenicity=81.2% [60.2,100]	n=16

Heuristic DD1 considers variants occurring at 5′SS with `GC` dinucleotides. `GC` 5′SS are still recognized, although weakly, by the major spliceosome, and should not be confused with the minor spliceosome. Although `GC` 5′SS are found in only about 1% of human introns[30], it is important to distinguish variants impacting a `GC` canonical dinucleotide 5′SS from those affecting a canonical `GT` motif. A `GC` is a much weaker motif for a 5′SS than a `GT` due to a mismatch between `GC` and the U1 snRNA spliceosome recognition component. This gives rise to a notable feature of `GC` 5′SS: much greater conservation of the motif than a `GT` 5′SS (Figure 9). Those conserved bases result in a closer match to the corresponding recognition nucleotides in U1 snRNA, presumably compensating for the `GC` 5′SS mismatch[30]. This makes the `GC`-type donor site vulnerable to weakening by variants occurring in the conserved bases. Additionally, these sites are more frequently involved in alternative splicing events than their `GT` counterparts[30].

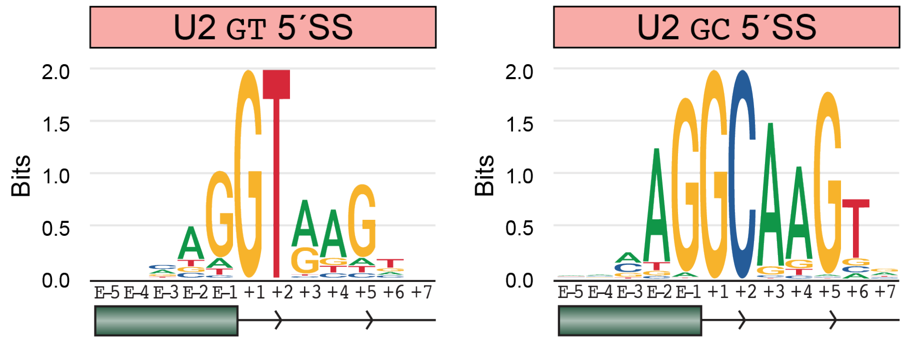

Figure 9: High sequence conservation of 5′SS with GC canonical dinucleotides. 5′SS with GC canonical dinucleotides, seen in the right-hand plot, exhibit very high sequence conservation (indicated by nucleotide symbol height) in the neighbouring base positions at E−3 to +6. Much lower sequence conservation is seen in their GT counterparts (left plot). GC 5′SS show 7 of the 9 positions with sequence conservation over 1 bit, corresponding to 80% occurrence in the motif. For plot terminology and construction, see Sequence Logos.

We note that traditional splice site strength measurements are not as applicable to GC 5′SS as the lack of +2T often scores below threshold. GC 5′SS return unfavorable scores with MaxEntScan, with the maximum score for a GC 5′SS of 3.24, corresponding to AAG|GCAAGT (as compared to 11.00 for AAG|GTAAGT). The highest scoring MaxEntScan motif corresponds to the "ideal" GC 5′SS consensus sequence, but some nucleotides are more important than others for 5′SS recognition, shown by the larger base symbols in Figure 9. This heuristic also considers a C at the E−3 position and variants converting the GC 5′SS to a GT 5′SS to be tolerated, resulting in the recognition sequence of (A/C)AG|G(C/T)AAGT.

Variants at the GC 5′SS gave outcomes of exon skipping (53%), exon truncation (17%), exon extension (17%), and intron retention (30%), shown on the heuristic DD1 panel.

Standard DD1 Variants at a GC 5′SS predominantly disrupt 5′SS recognition

Since the GC 5′SS donor region sequence (E−3 to +6) is constrained (Figure 9), even modest alterations in that region can alter splicing patterns. The DD1 Standard subgroup can apply to variants disrupting any location in a GC 5′SS. A variant at the +1 or +2 positions is classified as DD1 Standard unless it is a +2C>T. At other locations, the surrounding context sequence determines the classification of the variant and its outcome. However, if less than 8 positions in the sequence match the "ideal" motif ((A/C)AG|G(C/T)AAGT), the variant is classified as DD1 Standard, regardless of whether the variant causes the sequence to more closely resemble the ideal motif. 28 variants were observed in the DD1 Standard subgroup, with 100% spliceogenicity (with 95% confidence intervals 84.1 to 100%).

+2C>T variants, and variants that have 8 or the maximum 9 positions matching the ideal GC 5′SS motif (A/C)AG|G(C/T)AAGT, fall into the DD1 Contextual Modifier subgroup. For this subgroup, spliceogenicity was found to be 81.2% (with 95% confidence intervals 60.2 to 100%).

Variants changing +2C>T often improve donor recognition. Two such variants were present in SpliceVarDB, yet both were classified as 'Low-frequency splice-altering', causing exon inclusion to increase from ~92% to 100%. One paper studying 15 +2C>T variants has reported that 87% produced equivalent or increased levels of exon inclusion in transcripts[31]. Higher inclusion of an exon is not always well tolerated; variants causing increased exon inclusion interfere with alternative splicing and can lead to disproportional isoform expression. This phenomenon has been implicated in cancer[32], cardiac disease[33], and neurological diseases[34].

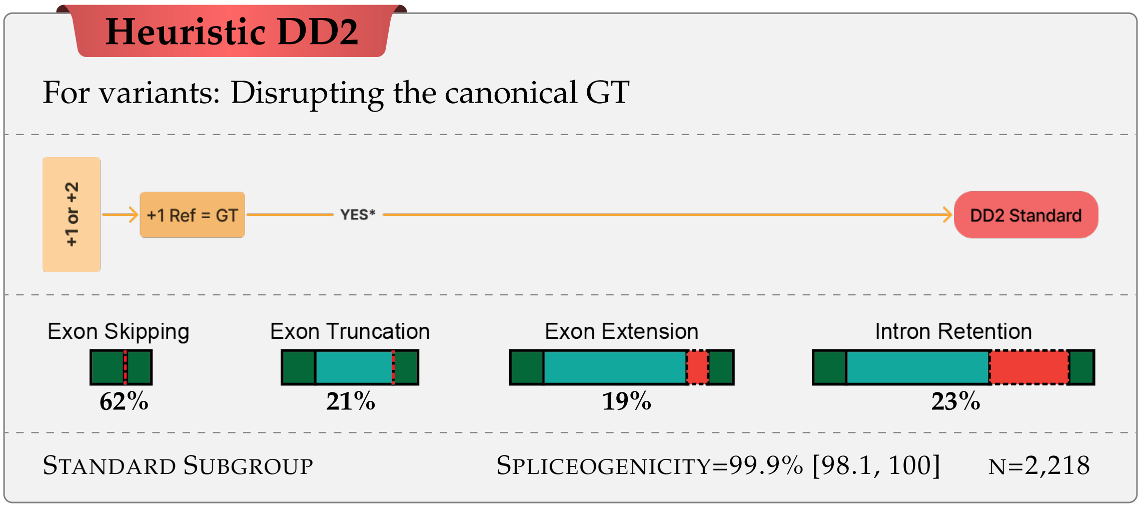

Heuristic DD2 considers variants that alter a canonical GT dinucleotide motif in the donor site. It is well established that these 5′SS variants alter splicing, so no additional steps are needed to classify variants at the canonical donor splice site; all variants are categorized under the DD2 Standard subgroup, where 99.9% were observed to alter splicing. 2,218 examples were considered in our study, and we observed outcomes of exon skipping (62%), exon truncation (21%), exon extension (19%), and intron retention (23%).

Variants that impact the canonical dinucleotides of the donor site do not always completely abolish the 5′SS. +2T>C variants can still result in normal splicing[35,36], as evident by the presence of GC 5′SS, explored in DD1 (Figure 9). However, this outcome depends on the motif being part of a sufficiently strong 5′SS that can tolerate the lack of a highly conserved base in the motif. Approximately 15% of +2T>C variants generate some normal transcripts (maximum observed was

84% of wildtype levels), but no examples showed equivalent or higher levels of 5′SS usage than the original GT[36]. In SpliceVarDB, there are 798 +2T>C variants. Of these, 80.2% are classified as 'Splice-altering', with the other 19.8% classified as 'Low-frequency splice-altering'. Thus, these variants are confidently splice-altering.

Heuristic DD3

For variants: Creating a TA at the third last base of the exon (E-3)

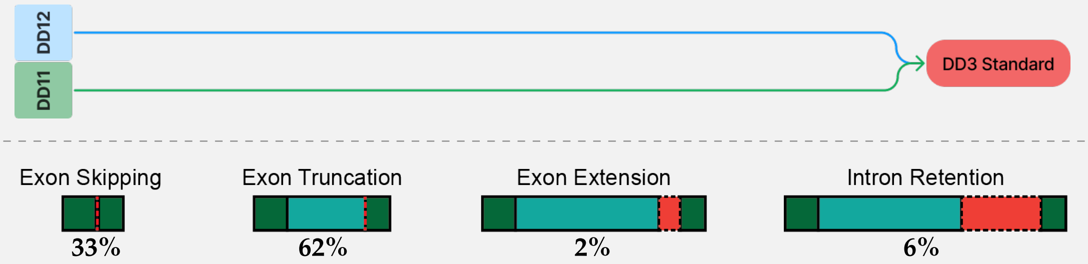

STANDARD SUBGROUP SPLICEOGENICITY=100% [90.5,100] N=78

Heuristic DD3 considers donor site variants that cause a TA dinucleotide to occur spanning positions E−3 and E−2 (i.e., E−3TA). These variants gave observed outcomes of exon skipping (33%), exon truncation (62%), exon extension (2%), and intron retention (6%). Variants classified using using heuristic DD3 are determined by DD11 (E−2) and DD12 (E−3).

A T at E−3 of the 5′SS is generally unfavorable and is found in only 11.7% of canonical protein-coding exons. However, despite A being the most common base at the E−2 position (64.5%), observing a TA dinucleotide at the E−3 and E−2 positions is relatively rare, seen in only 3.2% of exons examined (Figure 10). If these positions were not antagonistic, we would expect to observe this combination in approximately 7.5% of natural exons based on their standalone frequencies.

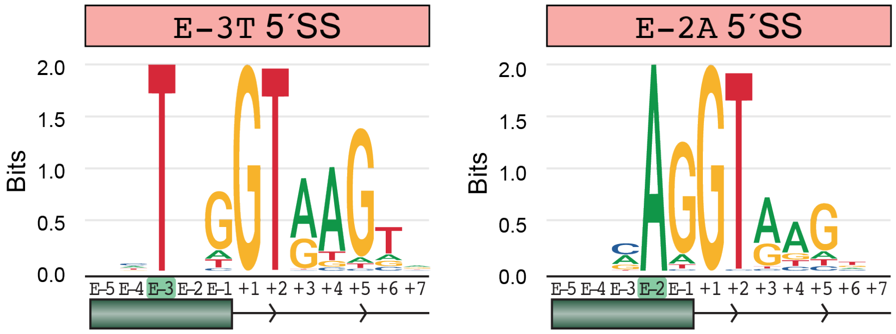

Figure 10: Low occurrence of E−3T in combination with E−2A. The left plot shows that the presence of E−3T results in a the lack of sequence conservation at the E−2 position, despite E−2A generally being conserved. The right plot shows that the presence of E−2A results in minor sequence conservation of all nucleotides except T at the E−2 position, indicating its unfavorability in combination with E−2A. For plot terminology and construction, see Sequence Logos.

It is interesting to note that TAG and TAA encode for stop codons, meaning that for approximately a third of all coding exons, E−3TA could result in a nonsense variant (depending on the reading frame).

We identified 78 variants that resulted in a E−3TA, all classed as DD3 Standard Subgroup. All were splice-altering, returning 100% spliceogenicity for (95% confidence interval of 90.5 and 100%).

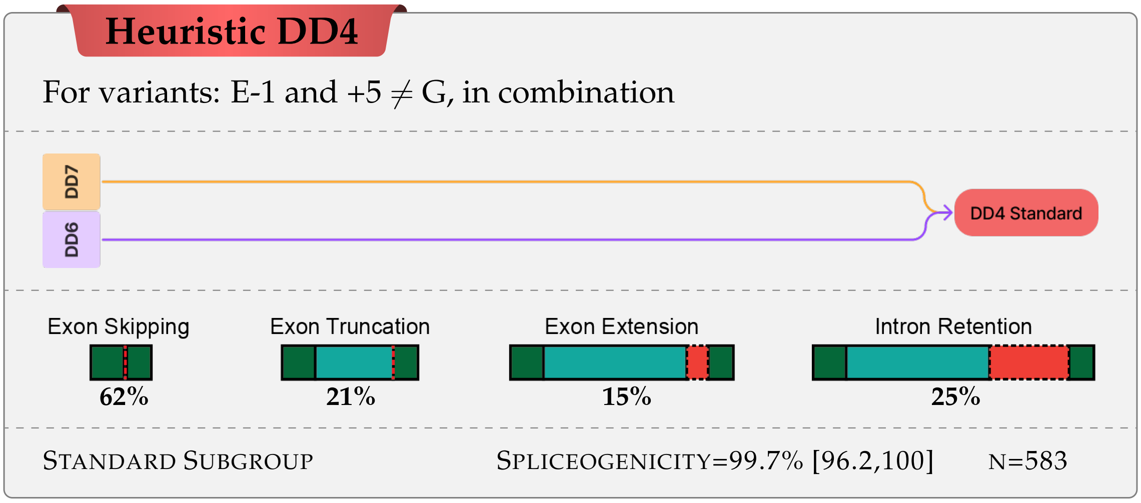

Heuristic DD4 considers variants affecting either E−1G or +5G which then results in a sequence containing both E−1≠G and +5≠G. For example, if E−1 is not a G, then a variant causing +5 also not to be a G would be classified under DD4. Heuristic DD4 is pointed to by heuristics DD6 (+5) and DD7 (E−1), requiring the use of the flowcharts provided in their respective panels to ensure the appropriate application of heuristic DD4. We thus categorized all these variants coming under heuristic DD4 as oneStandard subgroup.

Outside of the 5′SS canonical dinucleotide, the +5G and the E−1G are the most conserved bases that play a role in 5′SS recognition. Lack of their presence results in high sequence conservation for all remaining nucleotides in the 5′SS (Figure 11). The high conservation of E−1G and +5G has been suggested to arise due to their pairing with either U5 or U6 snRNA, which is a requirement for the spliceosome to recognize a 5′SS in the pre-catalytic stage of splicing[37].

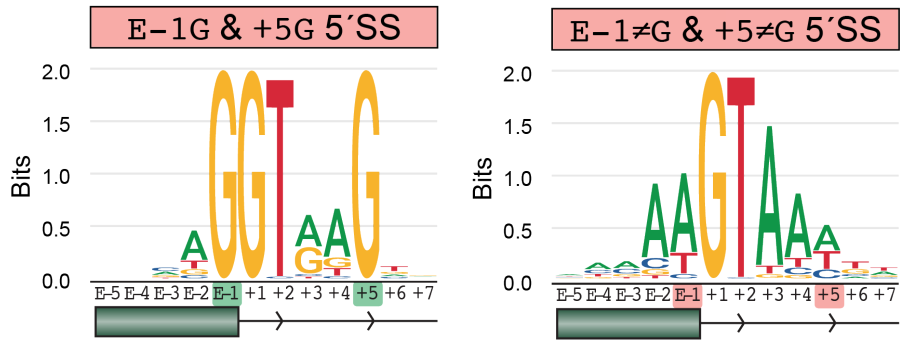

Figure 11: 5′SS sequence conservation in the presence of `E−1G` and `+5G`, compared to without. The left plot shows data from sequences where both `E−1G` and `+5G` are present, showing there is low sequence conservation for the rest of the 5′SS, other than the canonical dinucleotides. Conversely, `E−1≠G` and `+5≠G` (right plot) were associated with higher sequence conservation for all unaffected bases, primarily the preference of `A` throughout the 5′SS. For plot terminology and construction, see Sequence Logos.

We have observed that at least one of the `E−1` and `+5` sites is required to be a `G` for proper recognition of the 5′SS. Thus, when both are altered from `G`, the DD4 Standard subgroup is applied. Of 583 relevant variants examined, 99.7% were splice-altering (confidence intervals 96.2 to 100%). SAVs classified under DD4 returned the splicing outcomes of exon skipping (62%), exon truncation (21%), exon extension (15%), and intron retention (25%).

Heuristic DD5

For variants: Creating a CC or GT dinucleotide at the +3 position

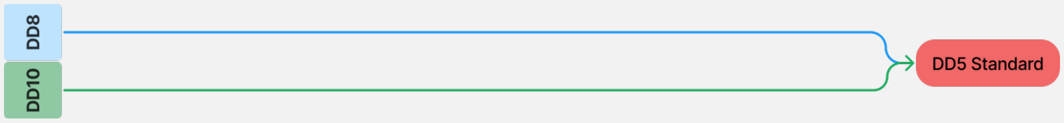

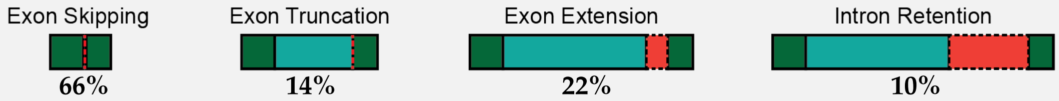

STANDARD SUBGROUP SPLICEOGENICITY=95.4% [87.3,100] N=108

Heuristic DD5 concerns variants that cause a `CC` or `GT` dinucleotide to occur spanning positions `+3` and `+4` (i.e., `+3CC`). The nucleotides at the `+3` and `+4` positions influence each other, with a strong preference for excluding `CC` or `GT` dinucleotides. These dinucleotide combinations are unfavored in naturally occurring splice sites (Figure 12).

The outcomes observed for variants in these positions were exon skipping (66%), exon truncation (14%), exon extension (22%), and intron retention (10%).

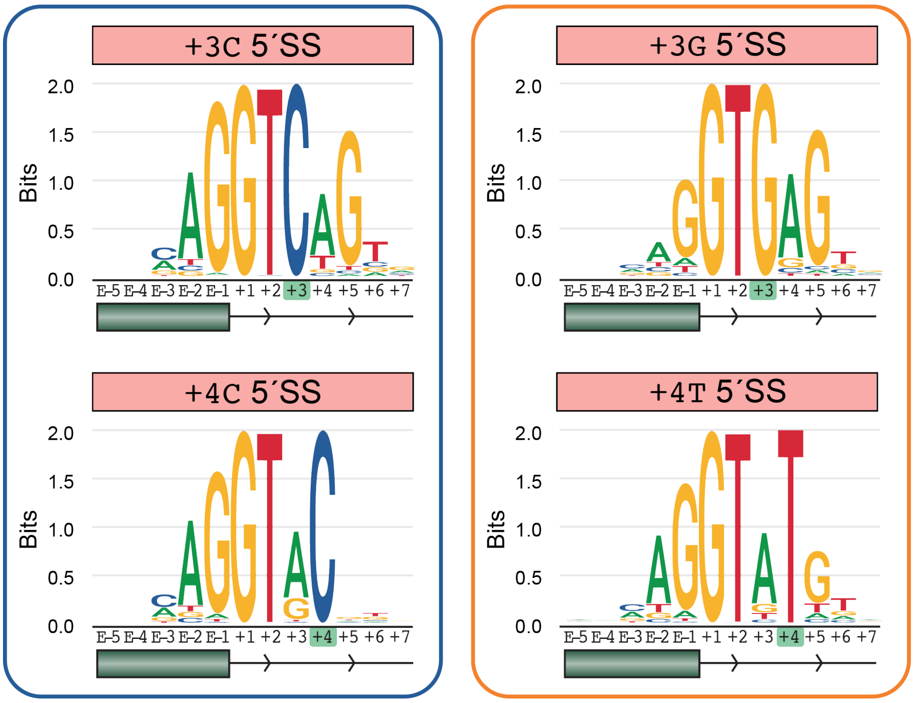

Figure 12: Low prevalence of +3CC and +3GT in 5′SS. Sequence conservation is shown for +3C, +4C, +3G, and +4T, noting that nucleotides shown boxed together are rarely seen in combination. For plot terminology and construction, see Sequence Logos.

108 variants were observed that conformed to this DD5 Standard subgroup, which displayed a spliceogenicity of 95.4% (95% confidence interval of 87.3 to 100%).

Heuristic DD6

For variants: Affecting the +5 position

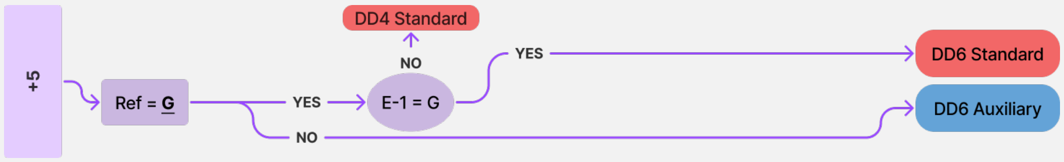

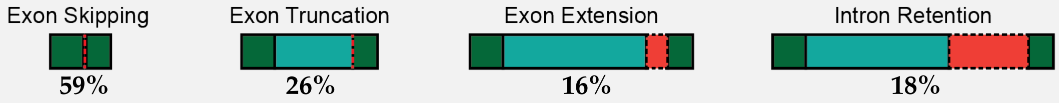

Standard Subgroup	Spliceogenicity=95.1% [91.1,99.1]	N=446
Auxiliary Subgroup	Spliceogenicity=28.6% [6.1,51.1]	N=14

Heuristic DD6 addresses variants affecting position +5. As noted in the DD4 heuristic, +5G is highly conserved (Figure 13). Indeed, +5 is the only splicing location outside of the canonical acceptor and donor dinucleotides to have its own Variant Effect Predictor (VEP) consequence:

'splice donor 5th base variant'[38].

There were 696 variants that affected the +5 location, and we observed outcomes of exon skipping (59%), exon truncation (26%), exon extension (16%), and intron retention (18%).

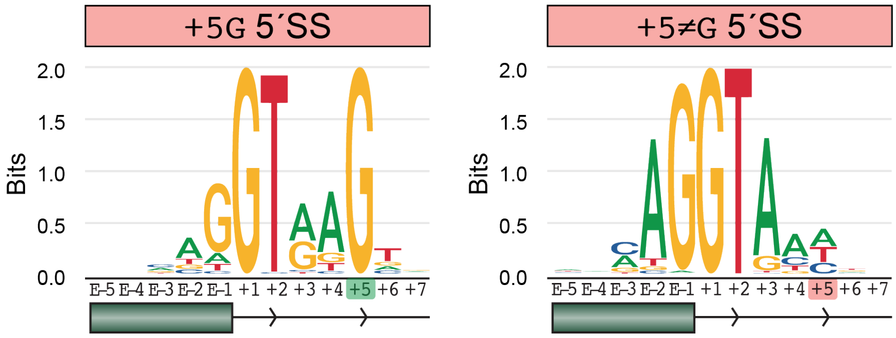

Figure 13: 5′SS sequence conservation for +5G compared to +5≠G. The left plot shows the pattern of conserved bases in sequences where there is a G at position+5; the right plot shows the same when a base other than G at that site. For plot terminology and construction, see Sequence Logos.

STANDARD DD6 VARIANTS AT THE +5 POSITION OF THE 5′SS WILL AFFECT SPLICING IF THE REFERENCE BASE IS A G

Where the reference sequence has a +5G and a E−1G, variants at the +5 position fall under this DD6 Standard subgroup if the base at E−1 position remains a G. 446 variants were observed in our dataset, with 95.1% of these variants altering splicing (95% confidence interval of 91.1 to 99.1%). Where both position +5 and E−1 are not G, heuristic DD4 should be consulted.

AUXILIARY DD6 VARIANTS AT E−1 WHERE THE REFERENCE SEQUENCE BASE IS NOT G ARE LESS LIKELY TO AFFECT SPLICING

We observed only 13 variants affecting position +5 when the reference sequence did not contain a G; these we classified by the DD6 Auxiliary subgroup. Only 28.6% of these altered splicing (95% confidence interval of 6.1 to 51.1%). Due to the small number of such variants, no further classification steps were made.

Heuristic DD7

For variants: Affecting the last base of the exon (E-1)

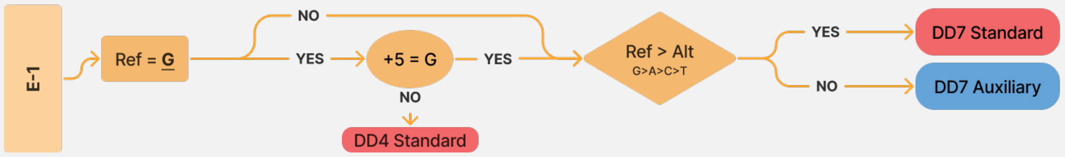

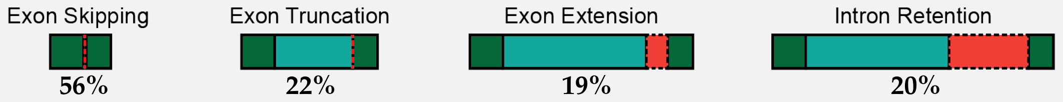

STANDARD SUBGROUP	SPLICEOGENICITY=94.0% [90.7,97.3]	N=667
AUXILIARY SUBGROUP	SPLICEOGENICITY=25.0% [0.7,49.3]	N=12

The significance of the final base of an exon in the splicing process has traditionally been underestimated, with greater emphasis placed on the alteration of the amino acid codon. 5′SS have a strong preference for the last base of an exon to be a `G`, as observed in 80% of naturally occurring exons (Figure 14). The order of prevalence of nucleotides at an exon's last base was determined to be `G>A>T>C`. However, when analyzing the experimentally-validated variants at this location, T>C variants were found not to alter splicing, whereas some C>T variants were. Therefore, we redefined the nucleotide preference order for being splice-altering at position `E-1` to be `G>A>C>T`.

If the reference sequence contains `E-1G` and `+5G`, this heuristic DD7 applies to any variant changing that `E-1G`. If position `+5` is not `G` in the reference sequence, heuristic DD4 should be consulted. This heuristic DD7 also applies to variants affecting `E-1` position where the reference sequence does not contain a `G`; in this case, the presence of `+5G` in the reference is not relevant. 679 variants falling under heuristic DD7 were considered in our dataset, and we observed exon skipping (56%), exon truncation (22%), exon extension (19%), and intron retention (20%).

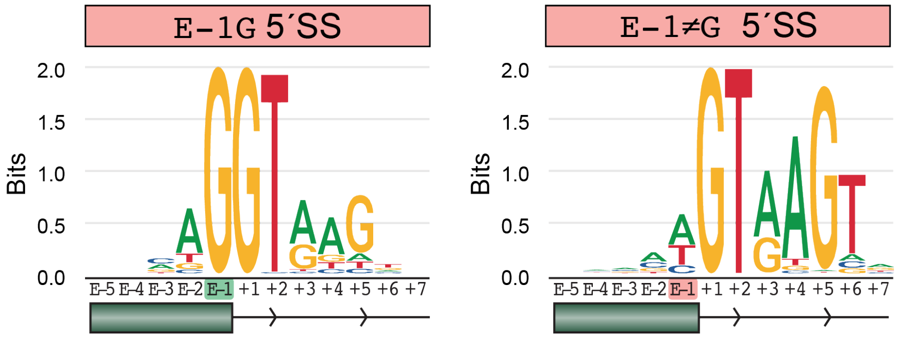

Figure 14: 5′SS sequence conservation for `E−1G` compared to `E−1≠G`. The left plot indicates the conservation of bases when a `G` is present at position `E−1`; the right plot shows a similar plot for sequences where a base other than `G` is at the site. For plot terminology and construction, see Sequence Logos.

Standard DD7 Variants at the `E−1` position decreasing the base preference affect splicing

The DD7 Standard subgroup concerns variants affecting position `E−1`, where the reference sequence shows `G` at the `E−1` and `+5` position; in this case, any variant at `E−1` conforms to the DD7 Standard subgroup. When the reference sequence does not contain `E−1G`, the DD7 Standard subgroup will only apply if a variant introduces a less preferred base; otherwise, the DD7 Auxiliary subgroup applies.

We observed 667 DD7 Standard subgroup variants in our dataset, which exhibited a spliceogenicity of 94% (95% confidence interval of 90.7 to 97.3).

Auxiliary DD7 Variants at the `E−1` position increasing the base preference are unlikely to affect splicing

DD7 Auxiliary subgroup variants were those that replaced the base at position `E−1` with a more preferred base, making splicing more likely to occur. Since the base preference was determined as `G>A>C>T`, an example would be reference `E−1T>G`, which would greatly favor splicing by installing the most preferred base at the `E−1` position. Only 12 DD7 Auxiliary subgroup variants were assessed, showing a low spliceogenicity of 25.0% (95% confidence interval of 0.7 and 49.3%). Given the small sample size, no further classification steps were set.

Heuristic DD8

For variants: Affecting the +3 position

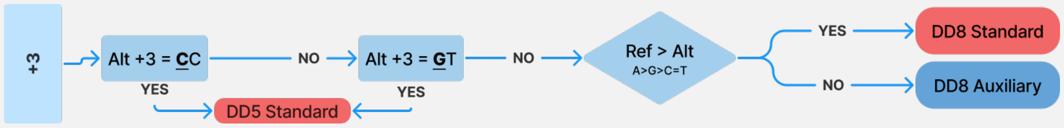

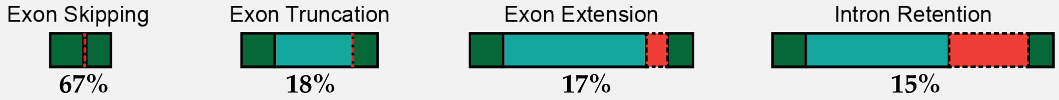

STANDARD SUBGROUP	SPLICEOGENICITY=93.6% [89.1,98.1]	N=344
AUXILIARY SUBGROUP	SPLICEOGENICITY=25.0% [6.2,43.8]	N=20

Heuristic DD8 concerns variants at the +3 position. If the variant, in combination with the base at position +4, does not create either a CC or a GT dinucleotide, then this heuristic DD8 applies. However, if it does create either CC or GT, then heuristic DD5 should be consulted. Statistical descriptions of the requirements for variants at the +3 position are detailed by Le Guedard-Mereuze *et al.*[39]. 364 variants conformed to heuristic DD8 in our datasets, and we observed outcomes of exon skipping (67%), exon truncation (18%), exon extension (17%), and intron retention (15%).

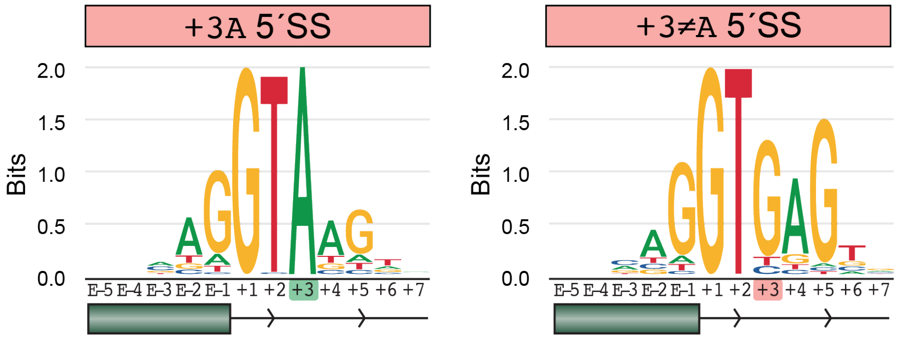

Figure 15: 5′SS sequence conservation patterns seen when +3A is present, compared to +3≠A. The left plot indicates observed base conservation in sequences where A is present at position +3; the right plot similarly shows sequence conservation where an A is not found at position +3. For plot terminology and construction, see Sequence Logos.

STANDARD DD8 VARIANTS AT THE +3 POSITION OF THE 5′SS ARE LIKELY TO AFFECT SPLICING IF THE ALTERNATIVE BASE IS LESS PREFERRED THAN THE REFERENCE.

We determined that the preference order at the +3 position of the donor splice site is A>G>C=T, with C and T being equally least favored. Figure 15 shows the base preference at this important location next to the conserved donor site GT dinucleotide. The base preference at the +3 site is

important for determining spliceogenicity of variants at this location. Where the reference base is more highly preferred than the alternative, the latter is classified as DD8 Standard subgroup; 344 variants fit in this subgroup and displayed a spliceogenicity of 93.6% (95% confidence interval of 89.1 to 98.1%).

Auxiliary DD8 Variants that increase the recognition of the 3′SS at the +3 position are likely to not affect splicing

Variants that increased or did not alter the preference were classified as the DD8 Auxiliary subgroup. Only 20 were considered, and 25% of these showed a likelihood of disrupting splicing (95% confidence interval of 6.2 to 43.8%).

Heuristic DD9

For variants: Affecting the +6 position

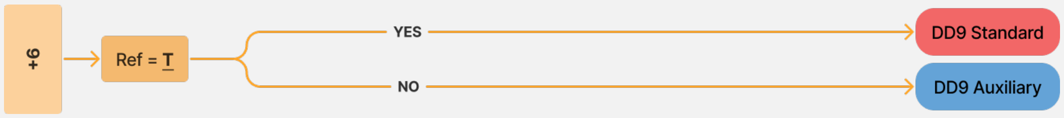

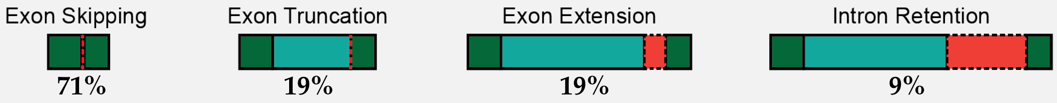

Standard Subgroup	Spliceogenicity=91.7% [84.0,99.4]	N=120
Auxiliary Subgroup	Spliceogenicity=19.0% [8.4,29.7]	N=63

Heuristic DD9 relates to variants at position `+6`. Details of the conserved bases around this site are shown in Figure 16, including the conserved `GT` donor splice site dinucleotide. There were 183 variants of this type, and we observed outcomes of exon skipping (71%), exon truncation (19%), exon extension (19%), and intron retention (9%).

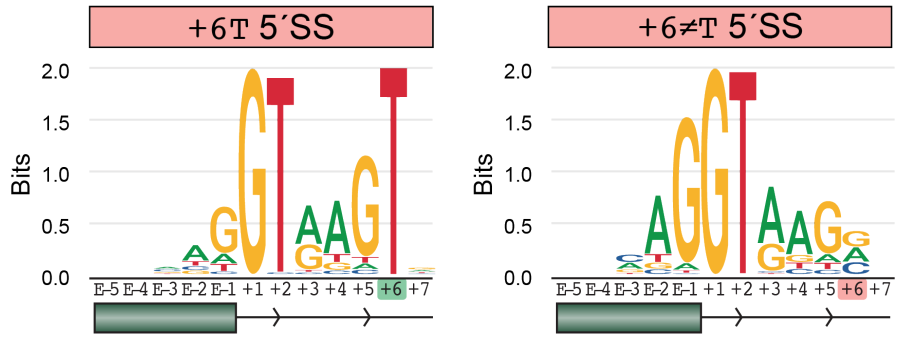

Figure 16: 5′SS sequence conservation of +6T compared to +6≠T. The left plot shows the conservation patterns where position +6 contains a T, while the right plot shows base conservation when it does not contain a T. For plot terminology and construction, see Sequence Logos.

STANDARD DD9 VARIANTS AT THE +6 POSITION WHERE THE REFERENCE BASE IS A T ARE LIKELY TO ALTER SPLICING

If the reference sequence shows a T at the +6 location, the DD9 Standard subgroup is applied. Spliceogenicity for the 120 DD9 Standard subgroup variants observed was 91.7% (95% confidence interval of 84.0 to 99.4%).

AUXILIARY DD9 VARIANTS AT THE +6 POSITION WHERE THE REFERENCE BASE IS NOT A T ARE UNLIKELY TO ALTER SPLICING

If the reference sequence does not show a +6T, then the DD9 Auxiliary subgroup applies. We saw only 63 such variants, for which there was a spliceogenicity of 19.0% (95% confidence interval of 8.4 to 29.7).

Heuristic DD10

For variants: Affecting the +4 position

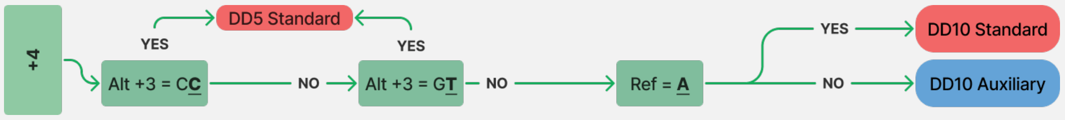

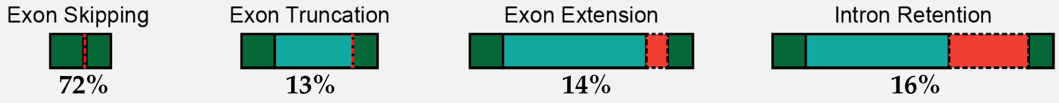

STANDARD SUBGROUP	SPLICEOGENICITY=91.5% [85,98.1]	N=165
AUXILIARY SUBGROUP	SPLICEOGENICITY=14.3% [0,30.2]	N=28

Heuristic DD10 concerns variants at position +4. Details of the conserved bases around this site are shown in Figure 17. This heuristic only applies if there is no base at the neighboring position +3 that will result in a CC or a GT dinucleotide; if there is then Heuristic DD5 should be consulted. There were 193 variants of this type, and we observed outcomes of exon skipping (72%), exon truncation (13%), exon extension (14%), and intron retention (16%).

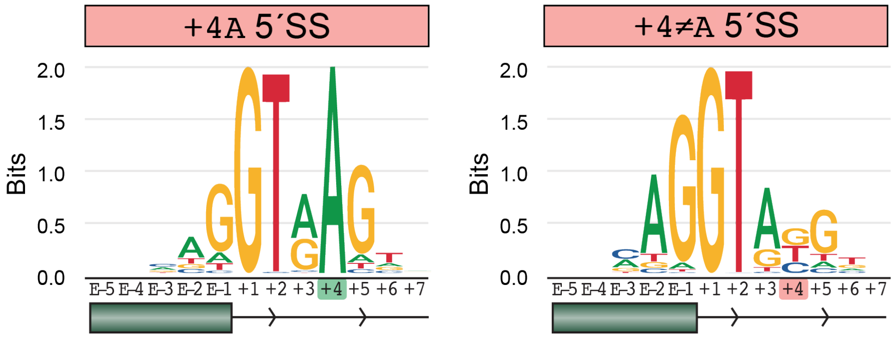

Figure 17: 5′SS sequence conservation of +4A compared to +4≠A. The left plot indicates sequences that contain +4A, while the right plot shows sequences with no A at position+4. For plot terminology and construction, see Sequence Logos.

STANDARD DD10 VARIANTS AT THE +4 POSITION OF THE 5′SS ARE LIKELY TO AFFECT SPLICING IF THE REFERENCE BASE IS AN A

If the reference sequence contains +4A then the DD10 Standard subgroup applies to a variant at this site. We found 165 of these variants, which displayed a spliceogenicity of 91.5% (95% confidence interval of 85 and 98.1%).

AUXILIARY DD10 VARIANTS ALTERING THE BASES UP THE PREFERENCE ORDER WILL NOT AFFECT SPLICING

If the reference sequence does not contain +4A then the DD10 Auxiliary subgroup should be applied to any variant at this location. There were only 22 examples of this type of variation, which had a much lower spliceogenicity of 14.3% (95% confidence interval of 0 and 30.2%).

Heuristic DD11

For variants: Affecting the penultimate base of the exon (E-2)

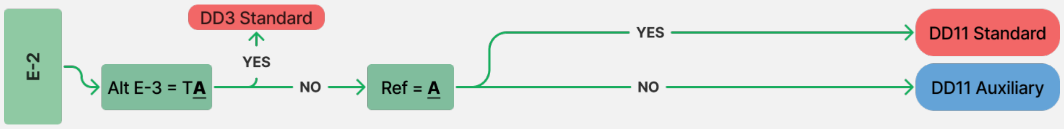

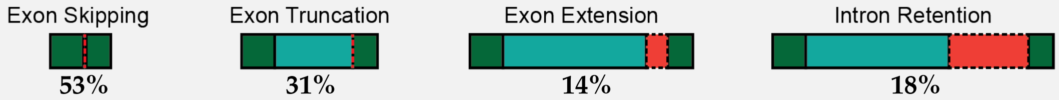

STANDARD SUBGROUP	SPLICEOGENICITY=91.1% [84.1,98.1]	N=146
AUXILIARY SUBGROUP	SPLICEOGENICITY=38.6% [27.4,49.7]	N=57

Heuristic DD11 applies to exon variants at position E−2. However, if a variant at the neighboring exon site of E−3 results in a TA dinucleotide, then heuristic DD3 (Standard) should be applied instead of DD11. We identified 203 DD11 variants, and observed outcomes were exon skipping (53%), exon truncation (31%), exon extension (14%), and intron retention (18%).

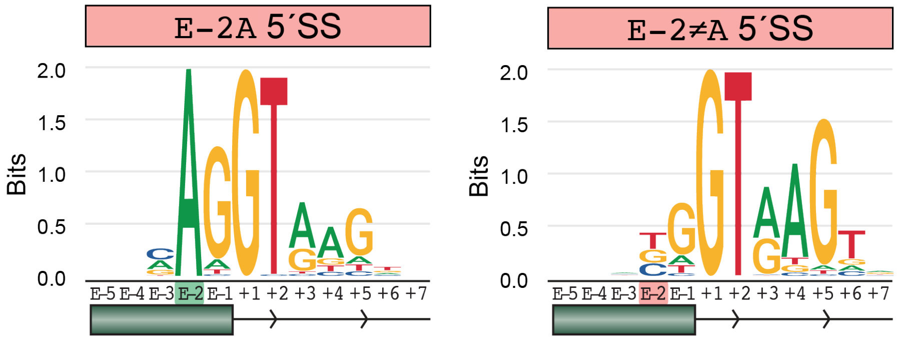

Figure 18: 5′SS sequence conservation of E−2A compared to E−2≠A. The left plot indicates the base conservation pattern seen when a E−2A is present, while the right plot shows the patterns when location −2 does not contain an A. For plot terminology and construction, see Sequence Logos.

STANDARD DD11 VARIANTS AT THE E−2 POSITION OF THE 5′SS ARE LIKELY TO AFFECT SPLICING IF THE REFERENCE BASE IS AN A

If the reference sequence contains E−2A then the DD11 Standard subgroup applies to a variant at this position. When the E−2 position does not contain an A, the remaining nucleotides at this position are not strongly selected for (Figure 18). We found 146 such variants, which showed spliceogenicity of 91.1% (95% confidence interval of 84.1 and 98.1%).

Auxiliary DD11 Other variants at the E−2 position have a small likelihood of altering splicing

If the reference sequence does not contain E−2A, then the DD11 Auxiliary subgroup applies to any variant at E−2. There were 57 such variants in our datasets, with a spliceogenicity of 38.6% (95% confidence interval of 27.4 and 49.7%).

Heuristic DD12

For variants: Affecting the third last base of the exon (E-3)

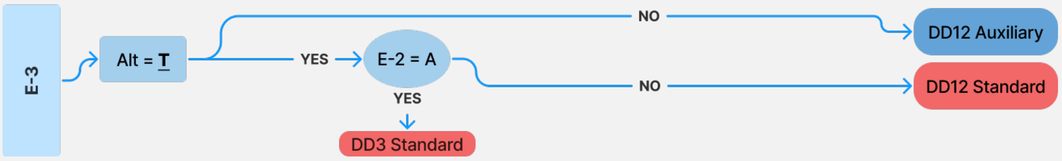

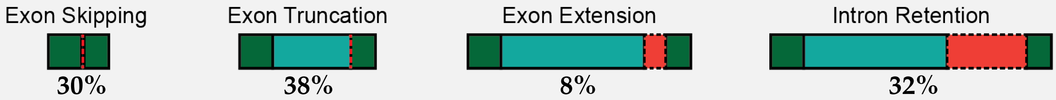

Standard Subgroup	Spliceogenicity=68.2% [50.2,86.1]	N=22
Auxiliary Subgroup	Spliceogenicity=45.3% [34.8,55.8]	N=64

Heuristic DD12 addresses variants located at exonic site E−3. Features of this region are shown in Figure 19. There were 86 of these variants, and the splicing outcomes were exon skipping (30%), exon truncation (38%), exon extension (8%), and intron retention (32%).

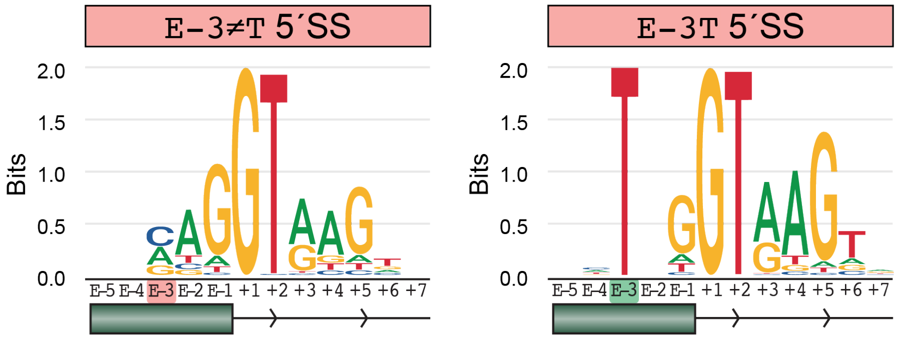

Figure 19: 5′SS sequence conservation for E−3T compared to E−3≠T. The left plot indicates the base conservation patterns we observed in sequences that do not contain T at position E−3, while the right plot shows these patterns where the sequences do contain T. For plot terminology and construction, see Sequence Logos.

Standard DD12 Variants introducing a T at the E−3 position alter splicing

Where there is a variant `T` at position `E−3`, then the DD12 Standard subgroup applies unless there is also an `A` at position `E−2`. In the latter instance, the DD3 Standard subgroup should be applied instead. We identified 22 variants conforming to the DD12 Standard subgroup with a spliceogenicity of 45.3% (confidence intervals 50.2 to 86.1%).

Auxiliary DD12 Remaining variants at the E−3 position of the 5′SS are moderately likely to affect splicing

For variants at position `E−3` that introduce a base other than `T`, the DD12 Auxiliary subgroup applies. We identified only 22 variants fitting this subgroup, which showed a spiceogenicity of 45.3% (confidence intervals 34.8 to 55.8%).

Disruption of the Acceptor (DA) Splice Site

Here, we detail heuristics that are more precise than the splicing checklist, which can be used for predicting whether a variant at an established 3′SS is an SAV. Unlike the donor splice site, recognition of the 3′SS relies on additional splicing elements outside the acceptor motif itself. Variants affecting the acceptor motif, the branchpoint, and the PPT are considered in the 3′SS disruption splicing heuristics below, as they are all necessary for 3′SS selection. Of the 6,764 experimentally-validated variants at the 3′SS, 61.1% (n=4,134) were SAVs. Applying the splicing requirements checklist to variants at the 3′SS revealed that 52% (n=2,151) render the original splice site unusable and 46% (n=1,919) weaken the original motif but leave it functional. The remaining SAVs (n=64) were anticipated (based on the splicing criteria) to strengthen the existing 3′SS.

The DA heuristics are presented below, first addressing the AGEZ and the 3′SS. They follow the numbering and diagram conventions used in figures 4 and 6 of the main paper (Sullivan *et al.*).

Heuristic DA1

For variants: Creating an AG dinucleotide upstream of the canonical AG

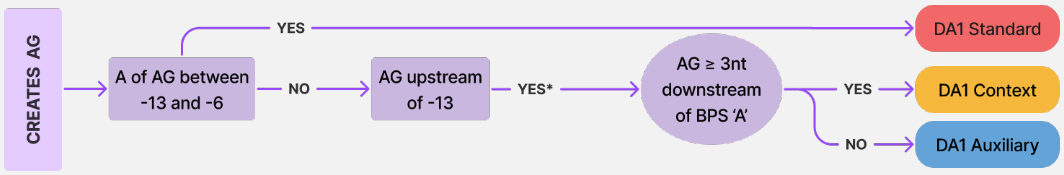

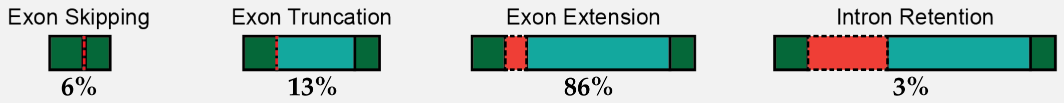

STANDARD SUBGROUP	SPLICEOGENICITY=99.7% [92.9,100]	N=304
CONTEXTUAL MODIFIER	SPLICEOGENICITY=78.0% [69.6,86.4]	N=100
AUXILIARY SUBGROUP	SPLICEOGENICITY=28.0% [22.5, 33.5]	N=232

Heuristic DA1 addresses variants introducing an AG dinucleotide upstream of the 3′SS. The region between the branchpoint and the 3′SS is known as the AG-exclusion zone (AGEZ)[10,40], since the 3′SS site is sensitive to disruption if AG dinucleotides exist or are introduced into this region; these variants, therefore, display high spliceogenicity[41]. Variant outcomes, relative to location, are summarised in Figure 20. We observed 636 of these variants resulted in exon skipping (6%), exon truncation (13%), exon extension (86%), and intron retention (3%).

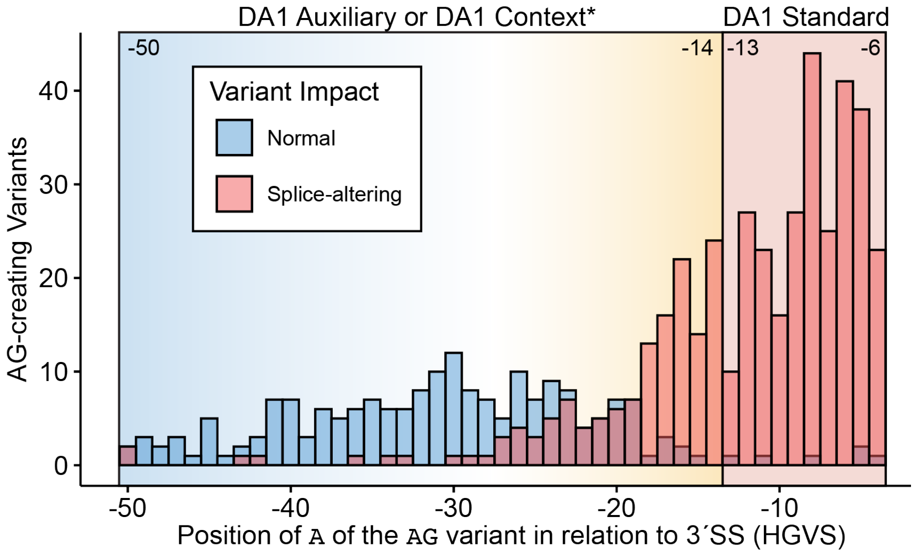

Figure 20: Splicing effect of AG-creating variants upstream of the 3′SS. The plot indicates the number of AG-creating variants at each location upstream of the 3′SS, coloured by their effect on splicing (Red: Splice-altering; Blue: Normal; Violet: both overlapping). The colour of the plot background indicates which DA1 subgroup likely applies (Red: Standard; Yellow: Contextual Modifier; Blue: Auxiliary). *DA1 Auxiliary and DA1 Context Modifier Rules are applied depending on the location of the AG-creating variant in relation to the branchpoint.

STANDARD DA1 CREATING AN AG DINUCLEOTIDE BETWEEN THE −13 AND −6 POSITION RESULTS IN ALTERED SPLICING

We observed that AG dinucleotides were under-represented between positions −13 and −6 upstream of the 3′SS (Figure 20). When a variant introduces an AG dinucleotide into the AGEZ, specifically when the A of the AG dinucleotide lies at or between −13 and −6, the variant is categorized as DA1 Standard. This is not dependent on the exact branchpoint location. These variants are expected to display high spliceogenicity[41] and consistently with this prediction, the 304 incidences of these AG variants conforming to the DA1 Standard subgroup gave a spliceogenicity score of 99.7% (95% confidence interval of 92.9 to 100%).

CONTEXT DA1 CREATION OF AN AG DINUCLEOTIDE AT LEAST 3 NUCLEOTIDES DOWNSTREAM OF THE BRANCHPOINT (YNANNAG) CAN DISRUPT SPLICING

The DA1 Contextual Modifier subgroup captures variants that create an AG dinucleotide upstream of position −13 and at least 3nt downstream of the closest useable (not less than -50nt) branchpoint (e.g., YNANNAG). Here, we define the branchpoint sequence (BPS) as a permissive sequence of YNA, though, as noted in Splicing Requirements, we have observed a high degree of sequence heterogeneity at the branchpoint with degenerate motifs often being used. If desired,

alternative techniques for locating branchpoints can be employed to implement heuristic DA1. 100 variants were observed in the DA1 Contextual Modifier subgroup, which showed 78.0% spliceogenicity (95% confidence interval of 69.6 and 86.4%), considerably lower than the DA1 Standard subgroup variants.

AUXILIARY DA1 CREATION OF AN AG DINUCLEOTIDE UPSTREAM OF THE BRANCHPOINT IS UNLIKELY TO ALTER SPLICING

This DA1 Auxiliary subgroup represents variants creating an AG dinucleotide upstream of the branchpoint. This occurrence is unlikely to disrupt the existing 3′SS unless it meets the requirements of a usable splice site that outcompetes the canonical splice site (see "Splicing Requirements"). Of the 232 variants that fit this Auxiliary subgroup, only a minority were splice-altering, resulting in a low spliceogenicity of 28.0% (95% confidence interval of 22.5 and 33.5).

Heuristic DA2

For variants: Disrupting the canonical AG

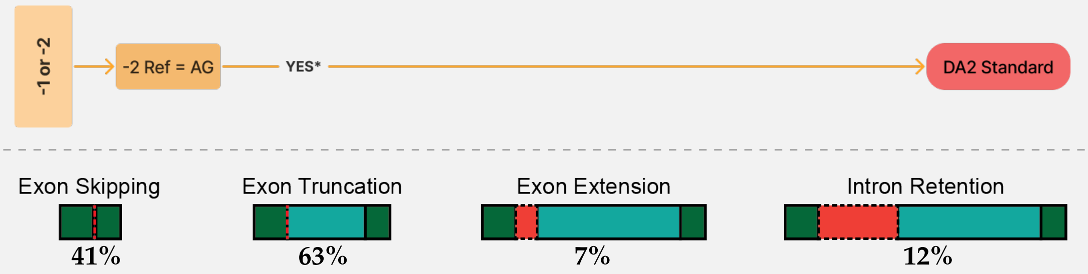

STANDARD SUBGROUP SPLICEOGENICITY=99.7% [98.1,100] N=2,817

Heuristic DA2 concerns variants at the canonical 3′SS; the high importance of this canonical AG dinucleotide means that disruption at this site is automatically identified as splice-altering, and only a Standard subgroup applies. We observed a spliceogenicity of 99.7% (95% confidence interval of 98.1 to 100%) for the 2,417 examples we examined. 65% of these showed evidence of exon skipping, 59% showed the use of a cryptic acceptor splice site (applicable if one exists that can be recognized by the spliceosome, see "Splicing Requirements"). Interestingly, an alteration of the canonical 3′SS was more likely to activate a cryptic exonic 3′SS (48%) than an upstream intronic 3′SS (11%). The DA2 Standard variants resulted in exon skipping (41%), exon truncation (63%), exon extension (7%), and intron retention (12%).

Heuristic DA3

For variants: First base of the exon (E+1) $\neq$ G and -3 $\neq$ C, in combination

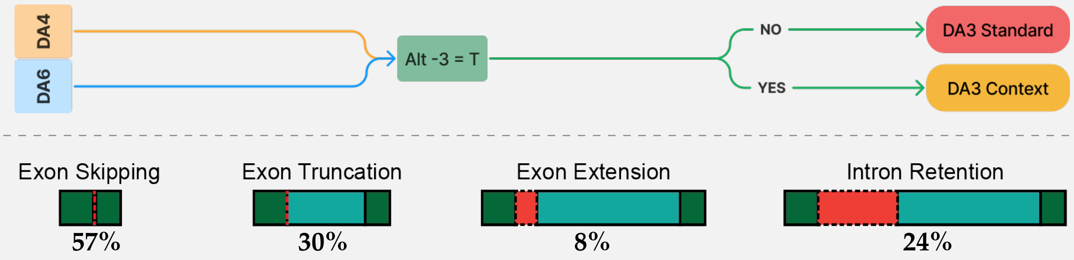

STANDARD SUBGROUP	SPLICEOGENICITY=99.2% [91.6,100]	N=124
CONTEXTUAL MODIFIER	SPLICEOGENICITY=70.4% [61.0,79.7]	N=81

Heuristic DA3 addresses variants that cause both the `E+1` not to be its preferred `G`, and the `−3` not to be its preferred `C` (Figure 21). This heuristic intersects with DA6 and DA4 heuristics. Variants that come under this heuristic (n=205) resulted in exon skipping (57%), exon truncation (30%), exon extension (8%), and intron retention (24%).

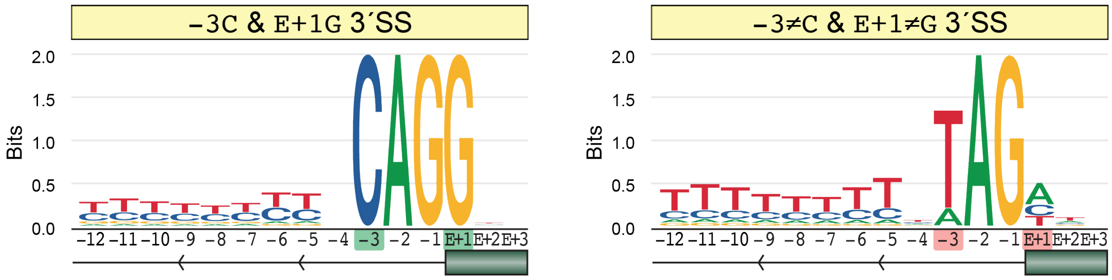

Figure 21: 5′SS sequence conservation of −3=C and E+1=G. The left plot shows base conservation in sequences containing both `−3C` and `E+1G`, while right plot shows the patterns when `−3` and `E+1` do not contain `C` and `G`, respectively. For plot terminology and construction, see Sequence Logos.

STANDARD DA3 VARIANTS RESULTING IN E+1 $\neq$ G IN COMBINATION WITH −3 $\neq$ C WILL ALTER SPLICING

Where the variant introduces a `G` or an `A` at position `−3`, the DA3 Standard subgroup applies. This also requires that position E+1 does not have a `G`. We observed 124 of these variants in our datasets that showed a high spliceogenicity of 99.2% (confidence interval of 91.6 to 100%).

Context DA3 A T at the −3 position can functionally replace −3 C

When a variant at position −3 introduces a T, then the DA3 Contextual Modifier subgroup holds. The 81 variants observed displayed a notably lower spliceogenicity of 70.4% (95% confidence interval of 61.0 and 79.9%), suggesting that the T confers some partial compensation for the lack of a C.

Heuristic DA4

For variants: Affecting the -3 position

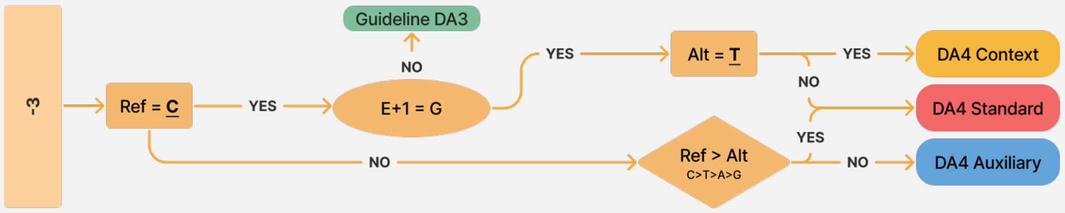

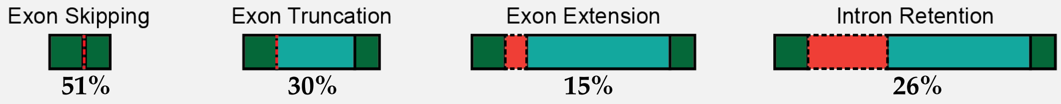

Standard Subgroup	Spliceogenicity=96.8% [90.1,100]	N=157
Contextual Modifier	Spliceogenicity=23.8% [5.4,42.2]	N=21
Auxiliary Subgroup	Spliceogenicity=17.4% [0,34.9]	N=23

Heuristic DA4 considers variants that introduce a change of nucleotide at the −3 position of the 3′SS when the first exon base (E+1) is a G. Note that if there is no G at the E+1 position then Heuristic DA3 should be applied rather than DA4.

These features at positions −3 and E+1 affect the ability of the acceptor splice site to be recognized by the spliceosome. An important feature is that at position −3 a C is most preferred, with the order of preference in our datasets being C>T>A≫G (Figure 22). We made observations from 201 variants that conformed to the DA4 heuristic type. Variants of this type resulted in exon skipping (51%), exon truncation (30%), exon extension (15%), and intron retention (26%).

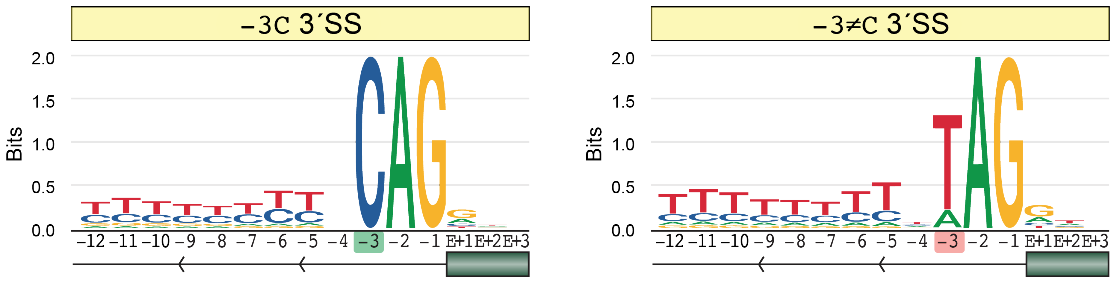

Figure 22: 5′SS sequence conservation of −3=C. The left plot indicates base conservation patterns in sequences that contain `−3C` while the right plot shows patterns seen in sequences not containing `C` at position `−3`. For plot terminology and construction, see Sequence Logos.

STANDARD DA4   VARIANTS AT THE `−3` POSITION WHILE POSITION `E+1=G`

The DA4 Standard subgroup applies if the reference sequence at position `−3` has a `C` and the variant changes this to a `G` or `A`; this also requires that the `E+1` position contains a `G` (Figure 22). In some cases, the reference sequence does not have a `C` at position `−3`. In these cases, the DA4 Standard subgroup still applies if the variant introduces a more preferred base at position `−3`, e.g., the reference is `G`, and the variant introduces `A`. We found 157 variants with DA4 Standard subgroup features, which showed a spliceogenicity of 96.8% (95% confidence interval of 90.1 to 100%).

CONTEXT DA4   AT POSITION `−3`, A C>T VARIANT IS LESS LIKELY TO BE SPLICE-ALTERING

When the reference sequence has `C` at position `−3` and the variant changes this to `T` (while position `E+1` is `G`), then the DA4 Contextual Modifier subgroup applies. We observed 21 cases of DA4 Contextual Midfier variants, with a much lower spliceogenicity than for the DA4 Standard subgroup, at 23.8% (95% confidence interval of 5.4 to 42.2%).

AUXILIARY DA4   VARIANTS AT `−3` THAT HAVE LOWER PREFERENCE WILL LIKELY NOT ALTER SPLICING

When the reference sequence at position `−3` is not `C` and the variant introduces a less preferred base at that position (e.g., the reference is `T` and the variant introduces `G`) then the DA4 Auxiliary subgroup applies. We found 23 instances of these variants in our datasets, and the spliceogenicity of 17.4% (95% confidence interval of 0 to 34.9%) was much lower than for the DA4 Standard variants.

Heuristic DA5

For variants: Affecting the bases at intron -5 or -6 positions

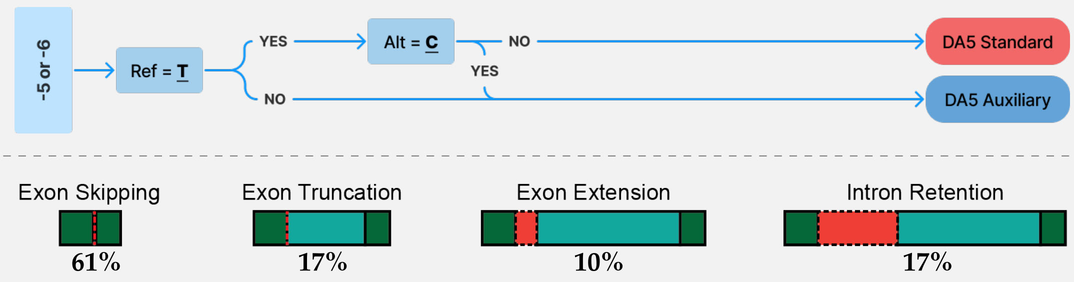

STANDARD SUBGROUP	SPLICEOGENICITY=93.1% [84.8,100]	N=102
AUXILIARY SUBGROUP	SPLICEOGENICITY=43.3% [36.7,49.9]	N=164

Heuristic DA5 applies to variants affecting position −5 or −6, which typically both contain T in the reference sequence. Variants that affected either site resulted in splicing outcomes of exon skipping (61%), exon truncation (17%), exon extension (10%), and intron retention (17%).

STANDARD DA5 VARIANTS AT EITHER POSITIONS −5 OR −6 THAT ALTER T TO BE AN A OR G ARE SPLICE-ALTERING

Variants follow the DA5 Standard subgroup if the reference sequence at positions −5 and −6 is TT and one of these was altered to a G or A. These variants showed a spliceogenicity of 93.1% (95% confidence interval of 84.8 to 100%).

AUXILIARY DA5 REMAINING VARIANTS AT THE −5 OR −6 LOCATIONS ARE NOT LIKELY TO BE SPLICE-ALTERING

Variants that change a reference T to a C at either location (−5 or −6) follow the DA5 Auxiliary subgroup and are less likely to be splice disrupting than those following the DA5 Standard subgroup. 164 variants following the DA5 Auxiliary subgroup were identified; for this type of variant we observed a spliceogenicity of 43.3% (95% confidence interval of 36.7 to 49.9%).

Heuristic DA6

For variants: Affecting the first base of an exon (E+1)

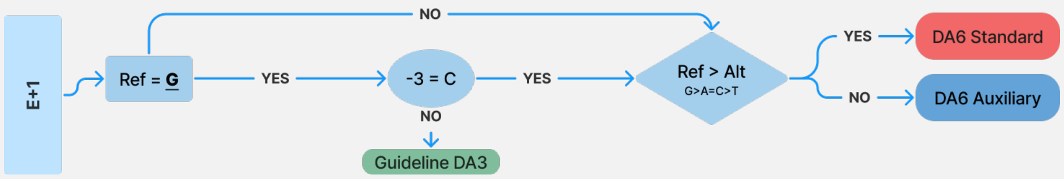

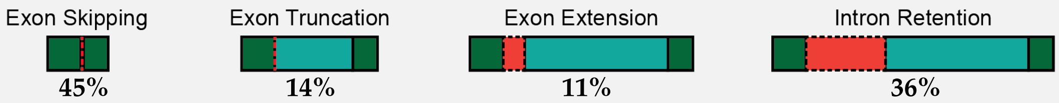

Standard Subgroup	Spliceogenicity=66.7% [58.1,75.3]	N=96
Auxiliary Subgroup	Spliceogenicity=14.8% [0,31.0]	N=27

Heuristic DA6 concerns variants affecting E+1, the first base in the 3′ exon. The main exonic base that plays a role in defining the location of the 3′SS is E+1, with a strong preference for G and a preference order of G>A=C>T (Figure 23). The 123 variants that conformed to the DA6 heuristic resulted in exon skipping (45%), exon truncation (14%), exon extension (11%), and intron retention (36%).

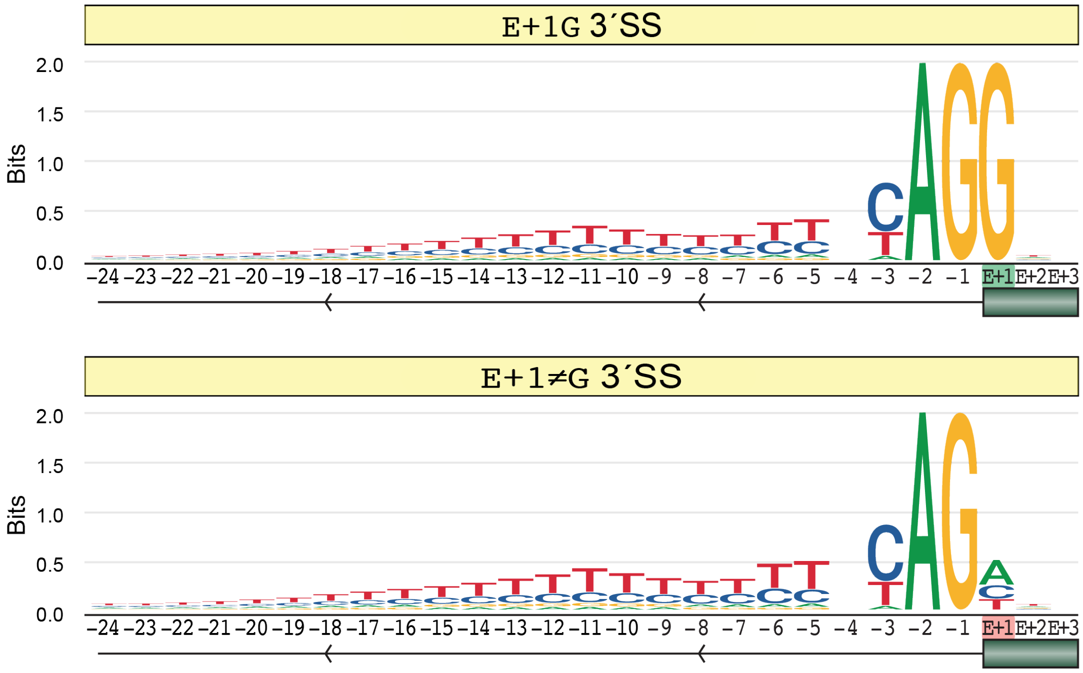

Figure 23: 3′SS sequence conservation of E+1G The top plot indicates the pattern of base conservation in the PPT and 3′SS sequence when E+1 is a G; the lower plot indicates the pattern when this is not the case. For plot terminology and construction, see Sequence Logos.

Variants at the E+1 site can be tolerated in the context of AG-independent introns[42]. AG-independent introns are rare in the human genome, and the absence of an AG at the 3'SS is compensated by having a strong PPT of 10-15nt (and perhaps other motifs). Despite this tolerance, our evaluations of PPT strength through metrics like polypyrimidine count, continuous polypyrimidine stretches, and thymine (T) content, did not improve our ability to predict the splice-altering potential of an E+1 variant.

It has been proposed that if the E+2 and E+3 positions have a high affinity to the U5 snRNA, and that they can compensate for the lack of a G at the E+1 position[37]. The proposed high-affinity bases are E+2C and E+3G. However, when we examined the 138 variants that disrupted an E+1G, there were no variants with both the E+2C and E+3G combination. Variants with one of E+2C or E+3G were slightly less likely to be splice-altering than variants with neither, although this difference was negligible (66.6% vs 72.9%).

STANDARD DA6 VARIANTS AT THE FIRST BASE OF AN EXON (E+1) ARE MODERATELY LIKELY TO AFFECT SPLICING IF THE ALTERNATIVE BASE IS LESS PREFERRED THAN THE REFERENCE

When a variant at the E+1 position is weaker (less preferred) than the reference nucleotide, the DA6 Standard subgroup applies. Spliceogenicity for the 96 observed examples was 66.7% (95% confidence interval of 58.1 to 75.3%). The confidence in predicting a splice alteration can be improved by taking into account the variant context. For example, the efficiency of core acceptor splice site recognition is influenced by the nucleotide at the −3 position, which preferably is a C. Thus, if a variant changes the E+1 to a weaker base (for example, from G to C), and there is a C at the −3 position DA6 Standard applies, but otherwise Heuristic DA3 should be consulted.

AUXILIARY DA6 E+1 VARIANTS WITH HIGHER PREFERENCE THAN THE REFERENCE WILL LIKELY NOT ALTER SPLICING

The DA6 Auxiliary subgroup concerns variants that strengthen the E+1 base (for example, from T to A). The 27 variants that fulfil this subgroup showed a spliceogenicity of 14.8% (95% confidence interval of 0 to 31%).

Heuristic DA7

For variants: In the branchpoint region

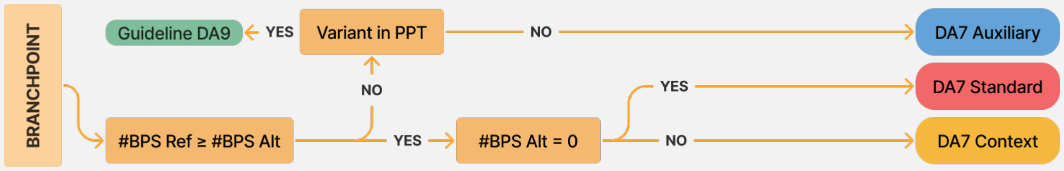

STANDARD SUBGROUP	SPLICEOGENICITY=38.9% [19,58.7]	N=18
CONTEXTUAL MODIFIER	SPLICEOGENICITY=19.2% [12.4,26.1]	N=151
AUXILIARY SUBGROUP	SPLICEOGENICITY=3.9% [1.2,6.6]	N=950

Heuristic DA7 addresses variants that affect the branchpoint. Recognition of a branchpoint is a crucial step in splicing, so any variants disrupting this process will impede lariat formation and intron excision. SpliceVarDB contained 85 SAVs out of a total of 1,119 validated variants in the region covered by DA7. However, very few studies have documented the splicing outcomes of such variants.

Of the 73 SAVs that were classified using DA7, only 5 had a reported splicing effect in SpliceVarDB. This small sample size is insufficient to establish reliable predictions regarding the splicing consequences of these variants.

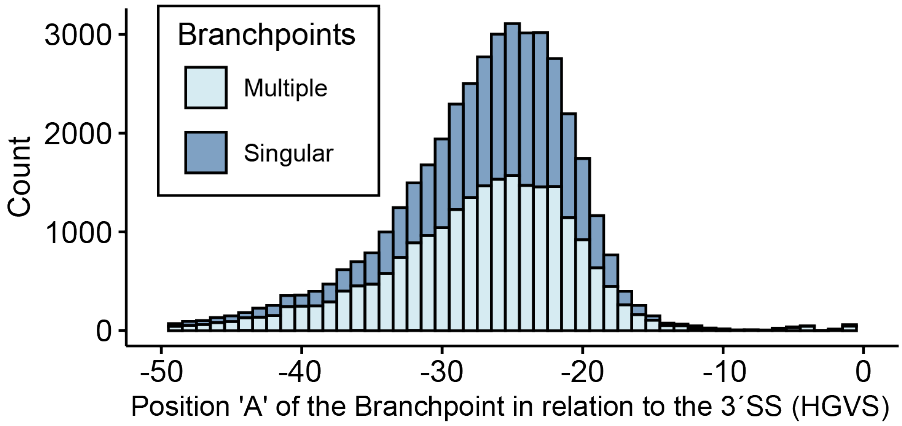

Figure 24: Location of the branchpoint within the 3′SS. The plot indicates the number of branchpoints (centred on A, branchpoint position 0) identified in our datasets, where either a single branchpoint was evident (dark blue) or where there may be multiple that could be usable (light blue).

STANDARD DA7 VARIANTS AFFECTING THE BRANCHPOINT MOTIF WILL SOMETIMES AFFECT SPLICING

As noted earlier, identifying a branchpoint motif can be challenging. Variants that disrupt a branchpoint will only affect splicing if no other branchpoint motif is present within the range of 17-50nt upstream of the acceptor splice site, a common occurrence (Figure 24). If a variant weakens a BPS, and there is no other BPS nearby, then the DA7 Standard subgroup applies. We observed 18 of these, with a moderate spliceogenicity of 38.9% (95% confidence interval of 19.0 to 58.7).

CONTEXT DA7 COMPENSATION BY OTHER BRANCHPOINTS REDUCES SPLICEOGENICITY

When a variant weakens a BPS, but an alternative, clear BPS exists within the branchpoint region (A of the branchpoint 17-50nt away from the 3'SS), the variant falls under the DA7 Contextual Modifier subgroup. In this scenario, the presence of an alternate BPS can compensate, diminishing the variant's potential impact on splicing. We observed 151 representative examples that demonstrated spliceogenicity of 19.2% (confidence interval 12.4 to 26.1%). Figure 24 highlights that many introns have more than one BPS.

AUXILIARY DA7 VARIANTS THAT CREATE OR STRENGTHEN A BPS RARELY AFFECT SPLICING

DA7 auxiliary concerns variants that strengthen an existing BPS, or create a new BPS. If such a variant falls within the PPT window, then heuristic DA9 should also be considered. For variants that fall outside the PPT window, among 950 examples, the spliceogenicity was just 3.9% (95% confidence interval of 1.2 to 6.6%).

Heuristic DA8

For variants: Affecting the -4 position

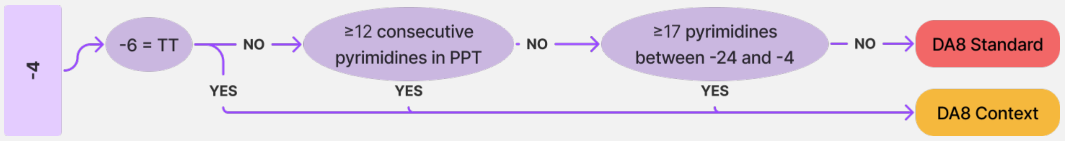

STANDARD SUBGROUP	SPLICEOGENICITY=33.3% [22.2,44.5]	N=57
CONTEXTUAL MODIFIER	SPLICEOGENICITY=7.7% [0,21.2]	N=39

Heuristic DA8 concerns variants affecting the −4 position. Our dataset from SpliceVarDB contained 96 variants of this type. However, only 16 splice-altering variants (SAVs) had well-documented

splicing outcomes, which restricted our capability to confidently predict the splicing implications associated with such variants.

Table 2: Splicing effects of variants at position −4. The proportion of variants that were splice altering, specified by the reference sequence (Ref) and variant sequence (Alt) nucleotides, of the 96 variants in our datasets that were classified under Heuristic DA8.

Ref Nucleotide \ Alt Nucleotide	A	C	G	T
A (23.8%)		0/3	2/17	1/3
C (27.2%)	1/6		2/8	5/17
G (20.6%)	3/16	2/5		5/12
T (28.4%)	0/2	1/3	0/4	

STANDARD DA8 VARIANTS AT THE −4 POSITION DO NOT GENERALLY AFFECT SPLICING

Variants consistent with the DA8 Standard subgroup did not have more than 12 consecutive pyrimidines (T or C) in the PPT, nor did they have 17 or more pyrimidines between positions −24 and −4. There were 57 variants consistent with these features that displayed a spliceogenicity of 33.3% (95% confidence interval of 22.2 to 44.5%).

CONTEXT DA8 IF A STRONG PPT IS PRESENT, OR THERE IS A TT AT −6, SPLICING WILL NOT BE AFFECTED

Variants in this subgroup had at least one of the three following features: 1) the presence of TT at position −6 to −5, 2) more than 12 consecutive pyrimidines in the PPT, and 3) at least 17 pyrimidines between positions −24 and −4. Spliceogenicity for these variants was very low at 7.7% (95% confidence interval of 0 to 21.2%), from a total of 39 variants from this subgroup.

Heuristic DA9

For variants: In the Polypyrimidine tract region

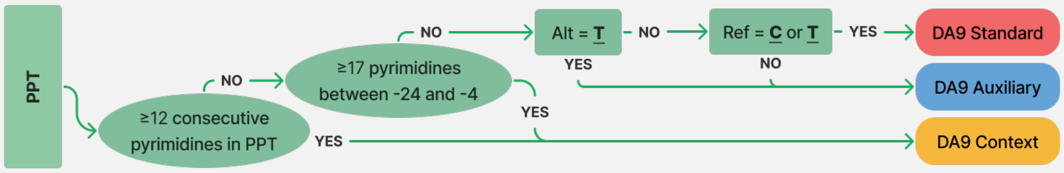

STANDARD SUBGROUP	SPLICEOGENICITY=25.4% [21.7, 29.1]	N=508
CONTEXTUAL MODIFIER	SPLICEOGENICITY=3.3% [0,10.1]	N=153
AUXILIARY SUBGROUP	SPLICEOGENICITY=4.6% [0.9,8.3]	N=524

Heuristic DA9 concerns variants in the PPT. Before proceeding with DA9, it is crucial to apply the heuristics outlined in 'Auxiliary DA6', as these variants may be situated in the branchpoint region. Evaluating the effects of a variant in the PPT is particularly challenging, as it is often unclear how or whether a minor modification to this large motif can lead to splicing alterations. Variants in this region are often overlooked as they can be very difficult to interpret, leading to a paucity of SAV examples. Nevertheless, it is useful to identify variants that are unlikely to alter splicing (for negative predictive value) and accumulate evidence of those that do.

A total of 1,185 variants fit into this heuristic subgroup. However, among these variants in SpliceVarDB there were few experimentally-validated SAVs, limiting the ability to reliably determine the splicing consequences linked to DA9.

STANDARD DA9 VARIANTS THAT REDUCE THE NUMBER OF PYRIMIDINES IN THE POLYPYRIMIDINE TRACT POTENTIALLY ALTER SPLICING

The DA9 Standard subgroup concerns variants that weaken a PPT, through the removal of a pyrimidine (`C` or `T`) from PPT's that have fewer than 12 consecutive pyrimidines and no more than 16 pyrimidines within the `-24` to `-4` region. We identified 508 variants of this type, which showed spliceogenicity of 25.4% (95% confidence interval of 21.7 to 29.1%). Further attempts to enhance the positive predictive value of this class of variants through additional stratification proved ineffective.

CONTEXT DA9 VARIANTS IN STRONG PPTS DO NOT AFFECT SPLICING

The DA9 contextual modifier subgroup concerns any variant affecting strong or long PPT, which we define has having at least 12 consecutive pyrimidines or at least 17 total pyrimidines in the `-24` to `-4` region. We identified 153 variants that fit into this subgroup, with a spliceogenicity of 4.6% (95% confidence interval of 0 to 10.1%).

AUXILIARY DA9 VARIANTS THAT STRENGTHEN PPT DO NOT AFFECT SPLICING

Variants introducing the preferred nucleotide `T` to the PPT, or a variant that replaces a purine (`G` or `A`) with a pyrimidine to a PPT that does not meet the definition of a strong PPT (see above), are classified in the DA9 Auxiliary subgroup. We identified 524 examples of these variants, which showed spliceogenicity of only 4.6% (95% confidence interval of 0 to 8.3%).

References

[1] Yeo, G. and Burge, C. B. (2004). Maximum Entropy Modeling of Short Sequence Motifs with Applications to RNA Splicing Signals. Journal of Computational Biology *11*, 377–394. 10.1089/1066527041410418.

[2] Wagih, O. (2017). ggseqlogo: a versatile R package for drawing sequence logos. Bioinformatics *33*, 3645–3647. 10.1093/bioinformatics/btx469.

[3] Mercer, T. R., Clark, M. B., Andersen, S. B., Brunck, M. E., Haerty, W., Crawford, J., Taft, R. J., Nielsen, L. K., Dinger, M. E., and Mattick, J. S. (2015). Genome-wide discovery of human splicing branchpoints. Genome Research *25*, 290–303. 10.1101/gr.182899.114.

[4] Turunen, J. J., Niemelä, E. H., Verma, B., and Frilander, M. J. (2013). The significant other: splicing by the minor spliceosome. WIREs RNA *4*, 61–76. 10.1002/wrna.1141.

[5] Moyer, D. C., Larue, G. E., Hershberger, C. E., Roy, S. W., and Padgett, R. A. (2020). Comprehensive database and evolutionary dynamics of U12-type introns. Nucleic Acids Research *48*, 7066–7078. 10.1093/nar/gkaa464.

[6] Wilkinson, M. E., Charenton, C., and Nagai, K. (2020). RNA Splicing by the Spliceosome. Annual Review of Biochemistry *89*, 359–388. 10.1146/annurev-biochem-091719-064225.

[7] Barash, Y., Calarco, J. A., Gao, W., Pan, Q., Wang, X., Shai, O., Blencowe, B. J., and Frey, B. J. (2010). Deciphering the splicing code. Nature *465*, 53–59. 10.1038/nature09000.

[8] Yıldırım, B. and Vogl, C. (2023). Purifying selection against spurious splicing signals contributes to the base composition evolution of the polypyrimidine tract. Journal of Evolutionary Biology *36*, 1295–1312. 10.1111/jeb.14205.

[9] Pastuszak, A. W., Joachimiak, M. P., Blanchette, M., Rio, D. C., Brenner, S. E., and Frankel, A. D. (2011). An SF1 affinity model to identify branch point sequences in human introns. Nucleic Acids Research *39*. 10.1093/nar/gkq1046.

[10] Wimmer, K., Schamschula, E., Wernstedt, A., Traunfellner, P., Amberger, A., Zschocke, J., Kroisel, P., Chen, Y., Callens, T., and Messiaen, L. (2020). AG-exclusion zone revisited: Lessons to learn from 91 intronic NF1 3 splice site mutations outside the canonical AG-dinucleotides. Human Mutation *41*, 1145–1156. 10.1002/humu.24005.

[11] Zhang, P., Chaldebas, M., Ogishi, M., Al Qureshah, F., Ponsin, K., Feng, Y., Rinchai, D., Milisavljevic, B., Han, J. E., Moncada-Vélez, M. *et al.* (2023). Genome-wide detection of human intronic AG-gain variants located between splicing branchpoints and canonical

splice acceptor sites. Proceedings of the National Academy of Sciences *120*, e2314225120. 10.1073/pnas.2314225120.

[12] Smith, C. W., Chu, T. T., and Nadal-Ginard, B. (1993). Scanning and competition between AGs are involved in 3′ splice site selection in mammalian introns. Molecular and Cellular Biology *13*, 4939–4952. 10.1128/MCB.13.8.4939.

[13] Chua, K. and Reed, R. (2001). An Upstream AG Determines Whether a Downstream AG Is Selected during Catalytic Step II of Splicing. Molecular and Cellular Biology *21*, 1509–1514. 10.1128/MCB.21.5.1509-1514.2001.

[14] Sohail, M. and Xie, J. (2015). Diverse regulation of 3 splice site usage. Cellular and Molecular Life Sciences *72*, 4771–4793. 10.1007/s00018-015-2037-5.

[15] Signal, B., Gloss, B. S., Dinger, M. E., and Mercer, T. R. (2018). Machine learning annotation of human branchpoints. Bioinformatics *34*, 920–927. 10.1093/bioinformatics/btx688.

[16] Leman, R., Tubeuf, H., Raad, S., Tournier, I., Derambure, C., Lanos, R., Gaildrat, P., Castelain, G., Hauchard, J., Killian, A. *et al.* (2020). Assessment of branch point prediction tools to predict physiological branch points and their alteration by variants. BMC Genomics *21*, 86. 10.1186/s12864-020-6484-5.

[17] Hort, Y., Sullivan, P., Wedd, L., Fowles, L., Stevanovski, I., Deveson, I., Simons, C., Mallett, A., Patel, C., Furlong, T. *et al.* (2023). Atypical splicing variants in PKD1 explain most undiagnosed typical familial ADPKD. npj Genomic Medicine *8*, 16. 10.1038/s41525-023-00362-z.

[18] Keegan, N. P., Wilton, S. D., and Fletcher, S. (2022). Analysis of Pathogenic Pseudoexons Reveals Novel Mechanisms Driving Cryptic Splicing. Frontiers in Genetics *12*, 806946. 10.3389/fgene.2021.806946.

[19] Conway, J. R., Lex, A., and Gehlenborg, N. (2017). UpSetR: an R package for the visualization of intersecting sets and their properties. Bioinformatics *33*, 2938–2940. 10.1093/bioinformatics/btx364.

[20] Davis, R. L., Homer, V. M., George, P. M., and Brennan, S. O. (2009). A deep intronic mutation in FGB creates a consensus exonic splicing enhancer motif that results in afibrinogenemia caused by aberrant mRNA splicing, which can be corrected in vitro with antisense oligonucleotide treatment. Human Mutation *30*, 221–227. 10.1002/humu.20839.

[21] Sangermano, R., Garanto, A., Khan, M., Runhart, E. H., Bauwens, M., Bax, N. M., van den Born, L. I., Khan, M. I., Cornelis, S. S., Verheij, J. B. G. M. *et al.* (2019). Deep-intronic ABCA4

variants explain missing heritability in Stargardt disease and allow correction of splice defects by antisense oligonucleotides. Genetics in Medicine *21*. 10.1038/s41436-018-0414-9.

[22] Bauwens, M., Garanto, A., Sangermano, R., Naessens, S., Weisschuh, N., De Zaeytijd, J., Khan, M., Sadler, F., Balikova, I., Van Cauwenbergh, C. *et al.* (2019). ABCA4-associated disease as a model for missing heritability in autosomal recessive disorders: novel noncoding splice, cis-regulatory, structural, and recurrent hypomorphic variants. Genetics in Medicine *21*, 1761–1771. 10.1038/s41436-018-0420-y.

[23] Rius, R., Riley, L. G., Guo, Y., Menezes, M., Compton, A. G., Van Bergen, N. J., Gayevskiy, V., Cowley, M. J., Cummings, B. B., Adams, L. *et al.* (2019). Cryptic intronic NBAS variant reveals the genetic basis of recurrent liver failure in a child. Molecular Genetics and Metabolism *126*, 77–82. 10.1016/j.ymgme.2018.12.002.

[24] Larrue, R., Chamley, P., Bardyn, T., Lionet, A., Gnemmi, V., Cauffiez, C., Glowacki, F., Pottier, N., and Broly, F. (2020). Diagnostic utility of whole-genome sequencing for nephronophthisis. npj Genomic Medicine *5*, 38. 10.1038/s41525-020-00147-8.

[25] Schalk, A., Greff, G., Drouot, N., Obringer, C., Dollfus, H., Laugel, V., Chelly, J., and Calmels, N. (2018). Deep intronic variation in splicing regulatory element of the ERCC8 gene associated with severe but long-term survival Cockayne syndrome. European Journal of Human Genetics *26*, 527–536. 10.1038/s41431-017-0009-y.

[26] Homolova, K., Zavadakova, P., Doktor, T. K., Schroeder, L. D., Kozich, V., and Andresen, B. S. (2010). The deep intronic c.903+469T>C mutation in the MTRR gene creates an SF2/ASF binding exonic splicing enhancer, which leads to pseudoexon activation and causes the cblE type of homocystinuria. Human Mutation *31*, 437–444. 10.1002/humu.21206.

[27] Rincón, A., Aguado, C., Desviat, L., Sánchez-Alcudia, R., Ugarte, M., and Pérez, B. (2007). Propionic and Methylmalonic Acidemia: Antisense Therapeutics for Intronic Variations Causing Aberrantly Spliced Messenger RNA. The American Journal of Human Genetics *81*, 1262–1270. 10.1086/522376.

[28] Jin, M., Li, J.-J., Xu, G.-R., Wang, N., and Wang, Z.-Q. (2020). Cryptic exon activation causes dystrophinopathy in two Chinese families. European Journal of Human Genetics *28*, 947–955. 10.1038/s41431-020-0578-z.

[29] Trabelsi, M., Beugnet, C., Deburgrave, N., Commere, V., Orhant, L., Leturcq, F., and Chelly, J. (2014). When a mid-intronic variation of DMD gene creates an ESE site. Neuromuscular Disorders *24*, 1111–1117. 10.1016/j.nmd.2014.07.003.

[30] Thanaraj, T. A. and Clark, F. (2001). Human GC-AG alternative intron isoforms with weak donor sites show enhanced consensus at acceptor exon positions. Nucleic Acids Research *29*. 10.1093/nar/29.12.2581.

[31] Lin, J. H., Masson, E., Boulling, A., Hayden, M., Cooper, D. N., Férec, C., Liao, Z., and Chen, J. M. (2020). 5 splice site GC>GT and GT>GC variants differ markedly in terms of their functionality and pathogenicity. Human Mutation *41*. 10.1002/humu.24029.

[32] Collesi, C., Santoro, M. M., Gaudino, G., and Comoglio, P. M. (1996). A Splicing Variant of the RON Transcript Induces Constitutive Tyrosine Kinase Activity and an Invasive Phenotype . Molecular and Cellular Biology *16*. 10.1128/mcb.16.10.5518.

[33] Loescher, C. M., Hobbach, A. J., and Linke, W. A. (2022). Titin (TTN): from molecule to modifications, mechanics, and medical significance. 10.1093/cvr/cvab328.

[34] Lee, C., Low, C. Y. B., Francis, P. T., Attems, J., Wong, P. T. H., Lai, M. K. P., and Tan, M. G. K. (2016). An isoform-specific role of FynT tyrosine kinase in Alzheimer's disease. Journal of Neurochemistry *136*. 10.1111/jnc.13429.

[35] Chen, J.-M., Lin, J.-H., Masson, E., Liao, Z., Férec, C., Cooper, D. N., and Hayden, M. (2020). The Experimentally Obtained Functional Impact Assessments of 5' Splice Site GT>GC Variants Differ Markedly from Those Predicted. Current Genomics *21*. 10.2174/1389202921666200210141701.

[36] Lin, J. H., Tang, X. Y., Boulling, A., Zou, W. B., Masson, E., Fichou, Y., Raud, L., Le Tertre, M., Deng, S. J., Berlivet, I. *et al.* (2019). First estimate of the scale of canonical 5 splice site GT>GC variants capable of generating wild-type transcripts. Human Mutation *40*. 10.1002/humu.23821.

[37] Artemyeva-Isman, O. V. and Porter, A. C. G. (2021). U5 snRNA Interactions With Exons Ensure Splicing Precision. Frontiers in Genetics *12*. 10.3389/fgene.2021.676971.

[38] McLaren, W., Gil, L., Hunt, S. E., Riat, H. S., Ritchie, G. R. S., Thormann, A., Flicek, P., and Cunningham, F. (2016). The Ensembl Variant Effect Predictor. Genome Biology *17*, 122. 10.1186/s13059-016-0974-4.

[39] Le Guédard-Méreuze, S., Vaché, C., Molinari, N., Vaudaine, J., Claustres, M., Roux, A. F., and Tuffery-Giraud, S. (2009). Sequence contexts that determine the pathogenicity of base substitutions at position +3 of donor splice-sites. Human Mutation *30*. 10.1002/humu.21070.

[40] Gooding, C., Clark, F., Wollerton, M. C., Grellscheid, S.-N., Groom, H., and Smith, C. W. (2006). A class of human exons with predicted distant branch points revealed by analysis of AG dinucleotide exclusion zones. Genome Biology *7*, R1. 10.1186/gb-2006-7-1-r1.

[41] Bryen, S. J., Joshi, H., Evesson, F. J., Girard, C., Ghaoui, R., Waddell, L. B., Testa, A. C., Cummings, B., Arbuckle, S., Graf, N. *et al.* (2019). Pathogenic Abnormal Splicing Due to Intronic Deletions that Induce Biophysical Space Constraint for Spliceosome Assembly. The American Journal of Human Genetics *105*, 573–587. 10.1016/j.ajhg.2019.07.013.

[42] Fu, Y., Masuda, A., Ito, M., Shinmi, J., and Ohno, K. (2011). AG-dependent 3-splice sites are predisposed to aberrant splicing due to a mutation at the first nucleotide of an exon. Nucleic Acids Research *39*, 4396–4404. 10.1093/nar/gkr026.
