# Supplementary material for: Data-driven insights to inform splice-altering variant assessment
Source: Am J Hum Genet. 2025 Mar 7;112(4):764–78. doi: 10.1016/j.ajhg.2025.02.012 (PMC12081236; doi:10.1016/j.ajhg.2025.02.012)
Supplement: Document S2. Article plus supplemental information [file mmc3.pdf]

ARTICLE

# Data-driven insights to inform splice-altering variant assessment


## Authors

Patricia J. Sullivan, Julian M.W. Quinn, Pamela Ajuyah, Mark Pinese, Ryan L. Davis, Mark J. Cowley

## Correspondence

mcowley@ccia.org.au



**Predicting the functional consequences of a putative splice-altering variant (SAV) is challenging. Here, we present a comprehensive framework for evaluating SAVs and predicting their functional consequences. It is grounded in known splicing rules and helps bridge the gap between *in silico* predictions and pathogenicity assessment.**



Sullivan et al., 2025, The American Journal of Human Genetics *112*, 764–778
April 3, 2025 © 2025 The Author(s). Published by Elsevier Inc. on behalf of American Society of Human Genetics.
https://doi.org/10.1016/j.ajhg.2025.02.012

ARTICLE

# Data-driven insights to inform splice-altering variant assessment

Patricia J. Sullivan,[1,2,3] Julian M.W. Quinn,[1] Pamela Ajuyah,[4] Mark Pinese,[1,2] Ryan L. Davis,[5,6,7] and Mark J. Cowley[1,2,7,*]

## Summary

Disease-causing genetic variants often disrupt mRNA splicing, an intricate process that is incompletely understood. Thus, accurate inference of which genetic variants will affect splicing and what their functional consequences will be is challenging, particularly for variants outside of the essential splice sites. Here, we describe a set of data-driven heuristics that inform the interpretation of human splice-altering variants (SAVs) based on the analysis of annotated exons, experimentally validated SAVs, and the currently understood principles of splicing biology. We defined requisite splicing criteria by examining around 202,000 canonical protein-coding exons and 19,000 experimentally validated splicing branchpoints. This analysis defined the sequence, spacing, and motif strength required for splicing, with 95.9% of the exons examined meeting these criteria. By considering over 12,000 experimentally validated variants from the SpliceVarDB, we defined a set of heuristics that inform the evaluation of putative SAVs. To ensure the applicability of each heuristic, only those supported by at least 10 experimentally validated variants were considered. This allowed us to establish a measure of spliceogenicity: the proportion of variants at a location (or motif site) that affected splicing in a given context. This study makes considerable advances toward bridging the gap between computational predictions and the biological process of splicing, offering an evidence-based approach to identifying SAVs and evaluating their impact. Our splicing heuristics enhance the current framework for genetic variant evaluation with a robust, detailed, and comprehensible analysis by adding valuable context over traditional binary prediction tools.

## Introduction

Genetic variants frequently influence gene splicing by altering splicing motifs, with 10%–30% of disease-causing variants estimated to affect splicing.[1] These splice-altering variants (SAVs) can lead to diverse functional consequences, such as exon skipping, intron retention, or the creation of novel splice sites, ultimately impacting the structure, function, or expression of the encoded protein.[2] Predicting the molecular consequence, and therefore pathogenicity, of a putative SAV is a crucial step in clinical variant interpretation. However, a major challenge lies in understanding the multifactorial complexity of mRNA splicing.

The process of splicing is orchestrated by the spliceosome, which is comprised of small nuclear RNAs (snRNAs) and over 100 proteins.[3] The splicesome assembles on target pre-mRNA by recognizing various splicing motifs that contain both essential and variable nucleotides. The core motifs involved in splicing include the acceptor splice site (3′SS) and donor splice site (5′SS), which flank the exon; the branchpoint sequence (BPS) that aids in lariat formation; and the polypyrimidine tract (PPT), a sequence of pyrimidine nucleotides (Cs or Ts) located between the BPS and the 3′SS.[4] These motifs work together with adjacent elements and require precise organization, strength, and spacing to facilitate the successful assembly and action of the spliceosome.[5] The disruption of this delicate balance by an SAV can lead to disease by causing the inclusion of intronic sequences or the exclusion of essential exonic sequences, both of which can have deleterious effects on the translated protein.[2]

The recognition of SAVs as an important disease-causing mechanism has resulted in considerable efforts toward enhancing the clinical variant interpretation guidelines established by the American College of Medical Genetics and Genomics (ACMG) and the Association for Molecular Pathology (AMP),[6] including updates by the ClinGen Sequence Variant Interpretation (SVI) Splicing Subgroup.[7–9] These studies offer important guidance on when to use specific ACMG evidence codes for SAVs. However, they do not provide guidance for interpreting the outcomes of SAVs, which is essential for classifying pathogenicity. In the absence of experimental validation, these recommendations rely heavily on *in silico* splice prediction tools, making the accuracy and limitations of these predictive algorithms a critical consideration.

The most accurate *in silico* splice effect prediction algorithms currently available, such as Pangolin[10] and SpliceAI,[11] are black-box artificial intelligence (AI) models. This makes unraveling their biological reasoning behind splice site usability predictions challenging. These models

[1]Children's Cancer Institute, Lowy Cancer Research Centre, UNSW Sydney, Sydney, NSW, Australia; [2]School of Clinical Medicine, UNSW Medicine & Health, UNSW Sydney, Sydney, NSW, Australia; [3]University of New South Wales Centre for Childhood Cancer Research, UNSW Sydney, Sydney, NSW, Australia; [4]Program in Medical and Population Genetics, Broad Institute of MIT and Harvard, Cambridge, MA, USA; [5]Neurogenetics Research Group, Kolling Institute, University of Sydney and Northern Sydney Local Health District, St. Leonards, NSW, Australia; [6]School of Medical Sciences, Faculty of Medicine and Health, University of Sydney, Sydney, NSW, Australia
[7]These authors contributed equally
*Correspondence: mcowley@ccia.org.au
https://doi.org/10.1016/j.ajhg.2025.02.012.
© 2025 The Author(s). Published by Elsevier Inc. on behalf of American Society of Human Genetics.
This is an open access article under the CC BY license (http://creativecommons.org/licenses/by/4.0/).

are trained on naturally occurring transcript sequences and splice junctions rather than experimentally validated variants, potentially hindering their predictive inference. This is demonstrated for variants that impact critical aspects of splicing that are not necessarily considered by *in silico* tools, such as the AG-exclusion zone (AGEZ)[12] or spacing requirements near the branchpoint,[13] both of which are well-established splicing rules.[14,15] Moreover, predictions from current *in silico* tools fail to offer reliable indications of a variant's effect on a transcript. This issue is exacerbated by the fact that the primary measure used by SpliceAI or Pangolin, the delta ($\Delta$) score, does not consistently mirror the actual outcome of an SAV. An illustrative example of this is an SAV in *PKD1* (c.11017-10C>A [GenBank: NM_001009944.3])[12] for which both Pangolin and SpliceAI correctly predicted disruption to the 3′SS, but they were not capable of extending the prediction to specify which nearby alternative splice site would be utilized in its absence (Figure S1). The spatial requirements of spliceosome assembly dictate that only one of the three alternative 3′SSs could be utilized, an outcome that was not favored by either algorithm. Due to their limited transparency, lack of multifactorial consideration (including the context a variant is found in), and inability to recapitulate known splicing rules, current models often fall short of accurately predicting the splicing-disruptive outcome of a variant.

While several studies describe the fundamental mechanisms of splicing,[5,16–18] a historical lack of large-scale supporting empirical evidence has hindered the centralization of splicing biology in AI models for *in silico* interpretation of SAVs. Researchers have had to rely on their splicing biology expertise to manually predict the splicing outcomes of potential SAVs, a process that lacked a systematic, evidence-based framework. To address this gap and provide a foundation for iterative improvements to SAV interpretation capability, we have established a set of heuristics grounded in robust splicing biology principles taken from large-scale empirical analysis of annotated splicing and splice-altering data. By analyzing relevant sequences around 183,723 annotated introns and 19,034 experimentally validated splicing branchpoints, we defined the characteristics of splicing motifs, including sequence constraints, spacing, and signal strength. We then utilized data from 11,868 experimentally validated SAVs from the SpliceVarDB (https://splicevardb.org)[19] to develop heuristics for informed SAV interpretation. Our insights begin to bridge the gap between *in silico* prediction of SAVs and the evaluation of their pathogenic effects, paving the way to filling the gap between existing approaches and properly holistic assessment of variant spliceogenicity.

## Material and methods

### Ethics approval

Ethics approval for this study was provided by the Hunter New England Human Research Ethics Committee of Hunter New England Local Health District in New South Wales, Australia (reference no. HC190484).

### Determining minimal splicing requirements

The motifs, intron/exon sizes, and positional requirements for splicing were determined using GENCODE v.44[20] filtered to exons from protein-coding genes tagged with "Ensembl canonical" ($n = 202{,}146$), including 5′ and 3′ untranslated regions (UTRs). Coordinates of the upstream and downstream exons were obtained using bedtools closest[21] to define the relative intron coordinates. Sequences were extracted from the UCSC hg38 reference genome using samtools faidx[22] with 50 nucleotide (nt) windows surrounding the annotated splice sites.

Introns excised by the minor spliceosome (U12) were identified using annotations from the Intron Annotation and Orthology Database.[23] These regions were considered separately from the major spliceosome (U2) due to differences in splice site recognition motifs. Separating the intron types yielded 183,089 U2 introns and 634 U12 introns for subsequent analysis.

Branchpoint locations and sequences were collected from a large-scale experimental branchpoint study.[24] The surrounding exons in GENCODE v.44lift37 (no filtering) were extracted using bedtools closest. Transcript IDs were matched between GENCODE v.44lift37 and the experimentally reported transcript. Sequences were extracted from the UCSC hg19 reference genome using samtools faidx with 100 nt windows encompassing the known splice sites and branchpoint locations. To ensure high-quality branchpoints and locations, branchpoint data were filtered to contain sequences with one branchpoint option and only one annotated 3′SS option closer than 50 nt downstream of the coordinate branchpoint, leaving 19,034 branchpoints for this analysis.

Sequence motif logos throughout this study were created using ggseqlogo.[25] Although splicing occurs at the RNA level, we use DNA sequences to represent the motifs, as most curators are attempting to interpret the effect of a putative SAV identified from DNA sequencing.

We adopted a splice variant nomenclature similar to the Human Genome Variation Society (HGVS) for reporting variant locations, relative to the closest exon-intron boundary (Figure 1A). For intronic changes, we use "+"to mark positions at an intron's 5′ end and "−" for positions at its 3′ end. For changes within exons, "E−" indicates positions at the exon's 3′ end, and "E+" marks positions at the 5′ end. Thus, the first base of an exon is E+1, the penultimate base of an exon is E−2, and the third base of the intron following the 5′SS is +3.

MaxEntScan scores were generated using maxentpy, a Python wrapper for MaxEntScan.[26] When considering the 3′SS and 5′SS in tandem, the combined splice site strength was measured across the intron.

When calculating the minimum and maximum distances for the splicing requirements criteria, the top and/or bottom 1% of the dataset was removed. The branchpoint maximum distance from the 3′SS was capped with the A of the branchpoint 50 nt from the 3′SS, as employed by the original data source.[24]

### Developing data-driven splicing heuristics

We took a direct approach to improve the identification of SAVs by determining splicing sequence patterns for experimentally

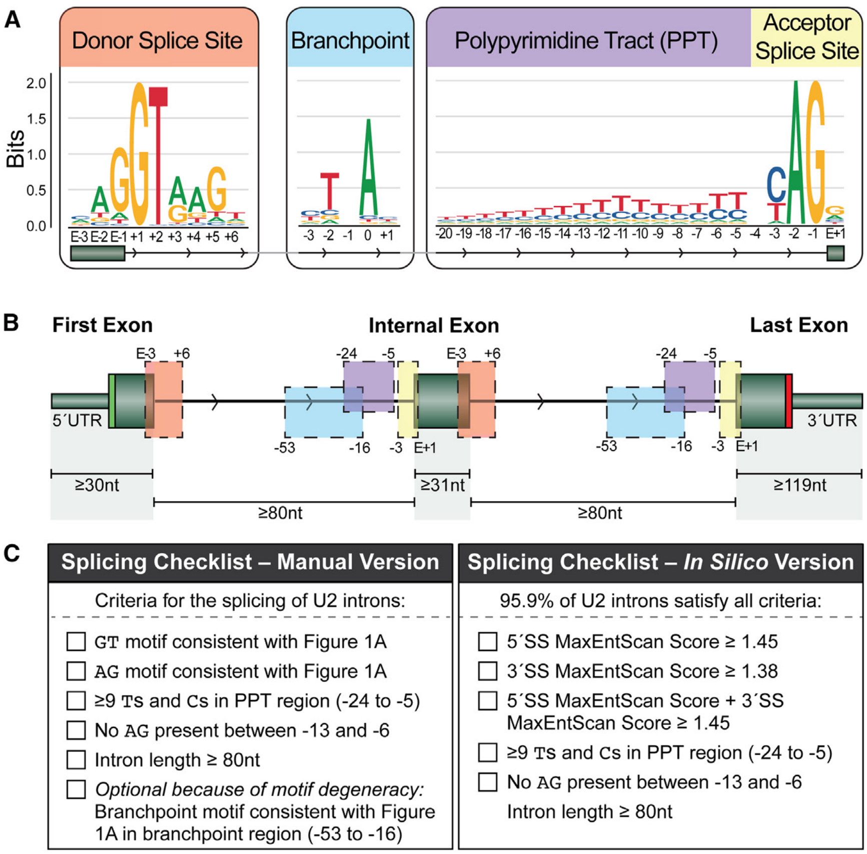


**Figure 1. Splicing requirements for the major spliceosome (U2)**

(A) Sequence logos of the four main splicing motifs in protein-coding genes. The height of a represented base (A, C, G, or T) indicates the relative sequence conservation at that position (bits), with 2 bits representing an essential or required base. The nucleotides are labeled using a naming system similar to HGVS (see material and methods).

(B) Relative location of splicing motifs and length of exons and introns. Green filled boxes represent exons, with the first, last, and internal exons displayed. Thinner sections of the first and last exons represent the untranslated regions (UTRs), demarcated by the start codon (light green) and stop codon (red). Black lines with arrows represent the introns and the direction (5′ to 3′). Colored boxes correspond to the locations of the motifs shown in (A), with the flanking numbers demarcating their start and end locations relative to the nearest exon. The minimum intron and exon lengths are annotated below.

(C) The splicing checklist, presented in both manual and *in silico* versions, for determining whether splicing will occur (see material and methods). All checklist items are required to be met unless specified otherwise. E+1, first nucleotide of the exon; E−3, third-last nucleotide of the exon; nt, nucleotide; PPT, polypyrimidine tract.

confirmed splicing variants. We obtained experimentally validated variants from the SpliceVarDB,[19] a database of >50,000 variants assayed for effects on splicing (downloaded August 19, 2024). We then filtered the data to focus on single-nucleotide variants (SNVs) that were either high-confidence SAVs labeled as "splice-altering" in the SpliceVarDB ($n = 8{,}920$) or variants with no observed splice-altering behavior, labeled as "normal" in the SpliceVarDB ($n = 2{,}948$). We then compared these groups to identify relevant differences. Genetic variants that fell in introns spliced by the minor U12 spliceosome were not included in this analysis, as there were too few to create heuristics for this variant type.

We then developed heuristics for assessing a variant's propensity to alter splicing according to its location within a gene. Variants that impacted the branchpoint or PPT motifs were grouped with the 3′SS variants. We defined three subgroups to guide variant interpretation: "standard," containing the variants most likely to be spliceogenic, "contextual modifier," where the surrounding sequence context impacts the splicing outcome, and "auxiliary," where a variant usually does not alter splicing. We endeavored to add contextual modifiers only if their inclusion led to a spliceogenicity separation of at least 15% between subgroups. At least 10 functionally validated variants informed each heuristic subgroup to establish reliability for determining splicing outcome(s) and avoid overfitting.

We defined spliceogenicity as the proportion of variants in each heuristic subgroup that were confirmed to be splice altering. In addition, we detail the number of genetic variants contributing to each subgroup ($n$) and the 95% confidence intervals (upper and lower bounds provided in square brackets) for spliceogenicity.

## Determining splicing outcomes

We utilized the standard splicing definitions of exon skipping, intron retention (where the entire intron is retained), and pseudoexon inclusion. We also employed the accepted terminology of "exon extension," where part of an intronic sequence is included, and "exon truncation," where part of an exonic sequence is excluded from the transcript.

Splicing outcomes for each heuristic were reported based on all SAVs in the SpliceVarDB, regardless of their heuristic subgroup classification. Specific splicing outcomes were only provided for a heuristic when over 20 SAVs had their impact on the transcript reported. Since we combined the subgroups to produce more robust outcome estimations, any deviations in outcome proportions between splicing subgroups were noted. As some SAVs led to more than one observed functional consequence, the outcomes can add up to more than 100%.

## Determining pseudoexon requirements

Variants from the SpliceVarDB that were denoted as creating a pseudoexon were used to determine whether the minimal splicing requirements defined above could be used to identify the mechanism of pseudoexon inclusion. We explicitly define pseudoexon-creating variants as those promoting the inclusion of an intronic region in a transcript that does not adjoin or overlap any canonical exon and is not normally detected in mature transcripts.[27] Our definition of canonical exons and mature transcripts was based on GENCODE v.44, although we note that some pseudoexons may be reclassified based on updates to transcript annotations. For the variants where a theorized mechanism of pseudoexon

creation was identified, we once again utilized the minimal splicing requirements to identify potential partner splice sites. If the variant activated a 3′SS, then we assessed all GT dinucleotides in the reference sequence within a window of 31–750 nt (corresponding to the minimum exon length vs. a practical upper limit) vs. the splicing checklist (see Figure 1C) to determine if any met the minimal splicing requirements.

All AG dinucleotides upstream of a created 5′SS and all GT dinucleotides downstream of a created 3′SS that satisfied the pseudoexon length criteria were assessed for suitability using the splicing checklist (see Figure 1C). Candidate partner splice sites were then filtered based on strength, keeping only those with a strength of at least 75% of the maximum MaxEntScan score. However, because of an observed preference for shorter pseudoexons,[28] we relaxed these requirements if the partner was within 200 nt of the newly created site with a MaxEntScan score $\geq$ 3.

## Results

### Splicing requirements

We analyzed regions around known canonical exons to ascertain the requisite sequence features that determine whether a splice site is likely to be utilized by the spliceosome. The major (U2) and minor (U12) spliceosomes recognize different motifs for the BPS, 3′SS, and 5′SS,[29] resulting in different splicing prerequisites. Thus, we focus here on the introns excised by the major spliceosome and refer the reader to the handbook (Data S1)—“minor spliceosome (U12) requirements”—for introns excised by the rare (0.5% of all introns) minor spliceosome. Of all the U2 introns in protein-coding canonical transcripts that were analyzed, we determined that 95.9% satisfy all of the defined requirements.

Recognition of the 5′SS is one of the first steps in spliceosome formation.[5] Consistent with previous reports, the 5′SS consensus sequence demarcating an exon to be spliced by the major spliceosome was determined as AG|GTRAG (where R is purine: an A or G nucleotide; “|” is the exon-intron boundary with the exon underlined throughout) (Figure 1A). The 3′SS had a consensus sequence of YAG|G (where Y is pyrimidine: a C or T nucleotide), preceded by a PPT of variable length (discussed in more detail below), which expands the 3′SS consensus sequence to $Y_n$NYAG|G (where *n* is typically between 10 and 20 and N is any nucleotide) (Figure 1A). Further upstream, but within 17–50 nt of the 3′SS, a branchpoint must be present. The branchpoint had a degenerate consensus sequence of TNA (where A is largely invariable at position 0 of the branchpoint, occurring in 91.8% of U2 branchpoints) (Figure 1A).[24]

Splicing is highly context dependent, and spliceosomal assembly, particularly at the 3′SS, necessitates the recognition of multiple motifs with specific positional constraints.[16] These positional constraints result in the creation of a window for each splicing motif (Figure 1B), the size of which was individually determined (Figure S2). The PPT occurs upstream of the acceptor motif, preferentially containing pyrimidines. When considering a broad PPT window between positions −24 and −5, the minimum number of pyrimidines required in that window was found to be nine (Figure S2D). We found that, while important for 3′SS recognition,[30] further PPT strength metrics, such as minimum uninterrupted polypyrimidines, thymine content, or maximum uninterrupted purines, were overly stringent to be applied as mandatory criteria.

The 3′SS is sensitive to AG dinucleotides between the branchpoint and canonical splice site, a region termed the AGEZ.[31] It has been shown that naturally occurring AG dinucleotides occur between the branchpoint and 3′SS in 14% of exons.[13] We observed that AG dinucleotides were depleted between positions −13 and −6 but were tolerated at the −5 position (AGYAG|G) (Figure S2F). This is due to the 3′SS AG-scanning recognition model, where the spliceosome loosely selects the first AG dinucleotide downstream of the branchpoint for lariat formation.[15] Subsequently, the precise identification of the 3′SS occurs, allowing for a stronger AG to be used if within 5 nt downstream of the first AG.[32] To further complicate the interpretation of the AGEZ, AG dinucleotides can be hidden by an RNA secondary structure within stem loops and still allow normal 3′SS recognition to occur.[15] Our heuristics do not account for RNA secondary structures, like stem loops, because these formations are uncommon and usually interfere with the recognition of the 3′SS.[15,33] In a more detailed assessment of AGEZ variants, Zheng et al. recently conducted a genome-wide study of AG dinucleotides occurring in the 3′SS region.[13]

Although there is no fixed location for the PPT, it is located between the branchpoint and the 3′SS motif, with the branchpoint’s A nucleotide located 17–50 nt upstream of the 3′SS. The branchpoint location is also a sequence marker used to determine the minimum intron length, as the “minimal intron theory” states that there must be greater than ~45 nt between the branchpoint and the closest upstream 5′SS.[34] A subsequent study further highlighted that distances shorter than 60 nt are associated with an increased risk of mis-splicing.[35] When combined with the minimal branchpoint distance from the 3′SS, in theory, the smallest intron capable of facilitating splicing is 62 nt, while introns of 77 nt or longer are less prone to mis-splicing. Furthermore, 99% of the exons we examined exhibited flanking intron lengths of at least 80 nt (Figure S2B), which is consistent with other studies[18]; hence, we adopted this value as our splicing prerequisite. Minimum exon lengths differed significantly depending on the exon number, with the last exon in a transcript generally having a much longer minimal length (99% were $\geq$119 nt) than the first exon (99% were $\geq$30 nt) or an internal exon (99% were $\geq$31 nt) (Figures 1B and S2A).

To consolidate the above splicing requirements, we created a splicing checklist that can be used to determine the minimal requirements for whether an exon is likely to be included in a transcript (Figure 1C). We have found

this checklist essential for interpreting the likely functional consequence of a given SAV, as it enables the evaluation of potential cryptic splice sites to determine if they are usable (Figure S1). This checklist serves as an initial, straightforward tool for assessing splice site viability. Below, we elaborate on heuristics for variants at established splice sites, where more rigorous criteria can be employed for analyzing a splicing motif already identified as recognizable.

We developed an *in silico* version of the checklist by translating the qualitative assessments for both the 5′SS and 3′SS into specific thresholds for MaxEntScan, a widely used *in silico* tool for evaluating the strength of splice sites (Figures 1C and S3). Although a branchpoint is essential for splicing, our checklists do not explicitly require their identification, as only 69.3% of experimentally determined BPSs are represented by the widely accepted branchpoint motif of TNA. While the branchpoint location can be more accurately predicted using specialized *in silico* tools, even the most accurate among these, Branchpointer,[36] has a low positive predictive value (PPV) of 30%,[37] indicating a high rate of false positives.

## Splice site disruption heuristics

This section presents heuristics for evaluating putative SAVs, their potential to disrupt splicing, and their likely impact. These heuristics have been developed using aggregated observations from 11,860 high-confidence, experimentally validated variants from the SpliceVarDB. To ensure the reproducibility of outcomes inferred from the splicing heuristics and avoid overfitting, we excluded instances that applied to fewer than 10 representative variants.

The heuristics are organized into categories that reflect distinct types of variant consequences: disruption of the 5′ donor SS (DD; see disruption of the 5′SS), disruption of the 3′ acceptor SS (DA; see disruption of the 3′SS), and creation of novel splice sites that can be assessed by applying the splicing requirements, with a section detailing specific applications to pseudoexons (see deep intronic variants and the creation of pseudoexons). While there were too few examples of experimentally confirmed SAVs that affect splicing regulatory elements (SREs) to make specific heuristics ($n = 103$), we anecdotally describe several examples from the literature (see SREs).

When developing the SpliceVarDB, we observed different levels of spliceogenicity when considering only the relative location of the SAV.[19] In this study, we expand on this by adding further classification steps to create subgroups of variants with similar spliceogenicity. Across heuristics, subgroups differ in spliceogenicity due to the abovementioned varying sensitivity of a location within a motif to disruption by genetic variation.

The core of each disruption heuristic starts with determining the variant location relative to an annotated splice site (see Figure 2A). The location of the variant determines which heuristics apply, such as DD4 and DD6 for a variant at the +5 position of the 5′SS (Figure 2A). Within each heuristic, variants are assigned to subgroups named according to the specific criteria used for classification rather than their spliceogenicity. All heuristics have a default standard subgroup designed to capture most SAVs impacting that location. However, splicing motifs are not recognized in isolation, and additional factors can affect the spliceogenicity of a variant. A contextual modifier subgroup was included to account for the influence of nearby variant-independent sequence context features on spliceogenicity. The auxiliary subgroup serves as a catchall for variants not included in either of these subgroups; most auxiliary subgroup variants represent variants less likely to be splice altering. We only added heuristic subgroups if doing so helped partition variants that would otherwise have diluted the overall spliceogenicity of the other subgroups.

Our framework provides a systematic approach for evaluating the splice-altering potential of genetic variants, providing both a measure of spliceogenicity and the anticipated splicing outcomes. To facilitate the use of these heuristics, we have devised a diagram that indicates which heuristic(s) should be applied based on the location of a variant (Figure 2), as well as step-by-step flowcharts for the disruption of the donor (Figure 3) and acceptor (Figure 4) splice sites. These heuristics can also be applied to insertions or deletions (indels), though the process is more complex, as multiple heuristics may apply (Figure S4).

We refer the reader to the handbook (Data S1) for a full description and in-depth exploration of every heuristic, while here we provide several examples of how the heuristics can be applied.

## Disruption of the 5′SS

Splice site selection at the 5′SS is dependent on the donor motif. Of the 5,104 experimentally validated variants at the 5′SS, 93.8% ($n = 4,786$) were SAVs, highlighting that the vast majority of variants in this region affect splicing. Applying the splicing requirements checklist to the SAVs revealed that 46% ($n = 2,205$) rendered the original splice site unusable and 53% ($n = 2,542$) weakened the original motif but left it functional. The remaining SAVs ($n = 39$) were anticipated, based on the splicing criteria, to either maintain the 5′SS strength or enhance it. Here, we have translated our insights into heuristics to inform whether a variant at an established 5′SS is an SAV (Figure 3). We illustrate the application of our donor splicing heuristics through four examples of increasing complexity.

### *Canonical dinucleotides of the 5′SS (+1 or +2)*

In the first example, we consider a variant altering the +1 or +2 position of the 5′SS. Any disruption to the canonical GT donor sequence at either the +1 or +2 position will be classified under the "DD2 standard" with a spliceogenicity of 99.9% 95% CI (98.1–100) abbreviated to [98.1,100] throughout ($n = 2,218$).

Non-canonical GC donor motifs are well described, and most variants will activate the heuristic "DD1 standard," with 100% [84.1–100] spliceogenicity ($n = 28$). The

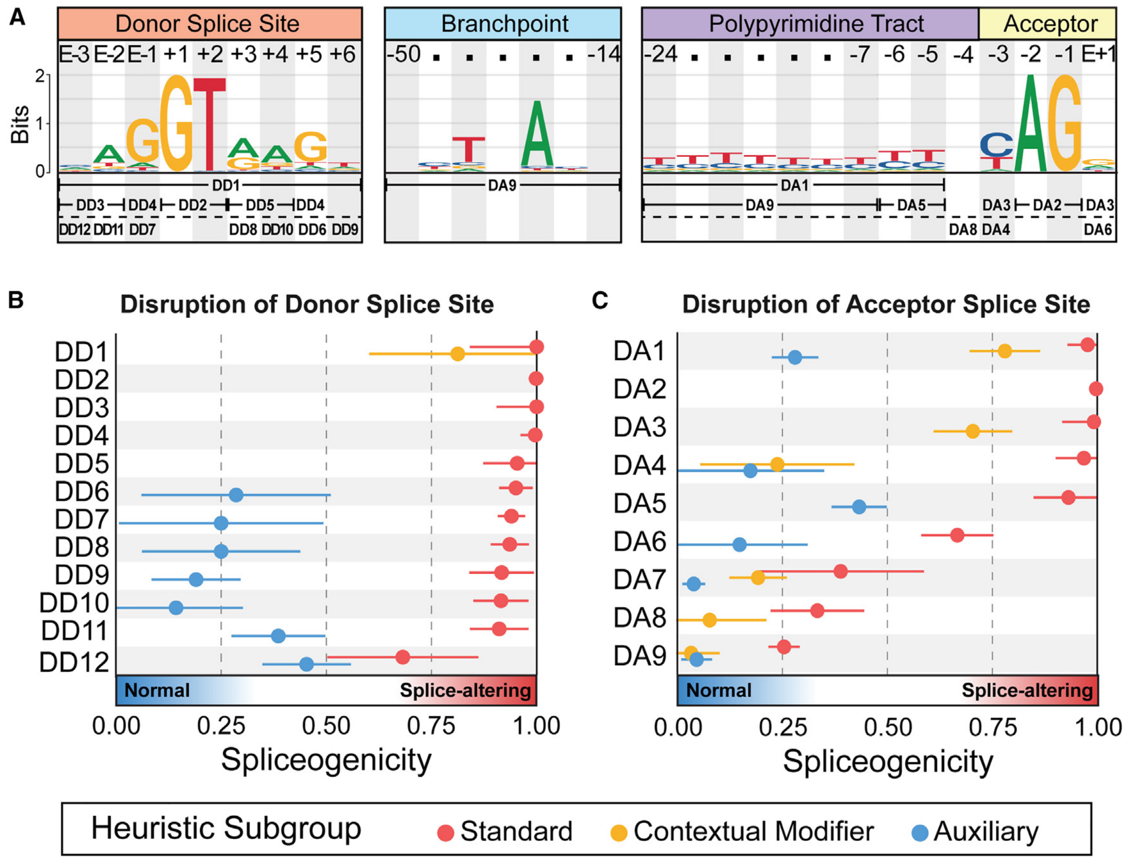


**Figure 2. Overview of splicing heuristics for donor and acceptor splice site disruption**

(A) Sequence logos for splicing motifs at the donor splice site, branchpoint, polypyrimidine tract, and acceptor splice site. These logos represent the consensus sequences and relative frequencies of nucleotides at each position within the splicing motifs. The relevant splicing heuristics (outlined in B and C) are indicated below their corresponding nucleotide location.

(B and C) Spliceogenicity (the likelihood of a splice-altering event occurring) of heuristics for variants that (B) disrupt the donor splice site (DD; 5′SS) and (C) disrupt the acceptor splice site (DA; 3′SS). The dots on the horizontal lines reflect the spliceogenicity level of each subgroup, while the extent of the line denotes the 95% confidence interval, illustrating the precision of the spliceogenicity estimate.

spliceogenicity decreases for variants that create a GT motif or those that create a motif with at least 8 of the most frequent nucleotides (see Figure 1A and the GC-exon-specific splice donor motif that accompanies the DD1 heuristic in the supplemental information). Both scenarios fall under the "DD1 context" heuristic with 81.2% [60.2–100] spliceogenicity ($n = 16$).

***Fifth intronic base of the 5′SS (+5)***

For the second example, we consider another well-established SAV location: the +5 position. The +5 position is the only splicing location outside of the canonical acceptor and donor dinucleotides to have its own Variant Effect Predictor (VEP) consequence ("splice donor 5th base variant").[38] However, variants at the +5 position are not as clear-cut as canonical dinucleotide variants, as variants at this position do not always affect splicing. For the variant type +5 of Figure 3, we determined that the reference nucleotide was the most effective indicator of whether a variant at this position will be splice altering. Following the flowchart, variants at +5 where the reference is not a G are not likely to be splice altering and are classified as "DD6 auxiliary" (spliceogenicity 28.6% [6.1–51.1]; $n = 14$). As only 14 variants fell into this auxiliary subgroup, no further sub-classification rules were defined.

Variants with G as a reference at +5 nearly always alter splicing, but the final base of the exon E−1 influences how often this happens. If E−1 is not a G, a variant causing +5 to also not be a G is classified under "DD4 standard" with a 99.7% [96.2–100.0] ($n = 583$) spliceogenicity. The presence of the G at E−1 slightly downgrades the spliceogenicity to "DD6 standard" with a 95.1% [91.1–99.1] spliceogenicity ($n = 446$).

***Last base of the exon (E−1)***

For the third example, we consider the effect of a variant at E−1 on splicing. The importance of this position in the splicing process has traditionally been underestimated, with greater emphasis around the impact on the associated amino acid encoded.[39] However, our analysis identified that 95% of variants at this position do indeed affect splicing, with a strong preference for a G at E−1 (Figure 1A). The frequency of nucleotides at E−1 in Figure 1A suggests a preference in the order G>A>T>C. However, when analyzing experimentally validated variants at this location, none of the five T>C variants in

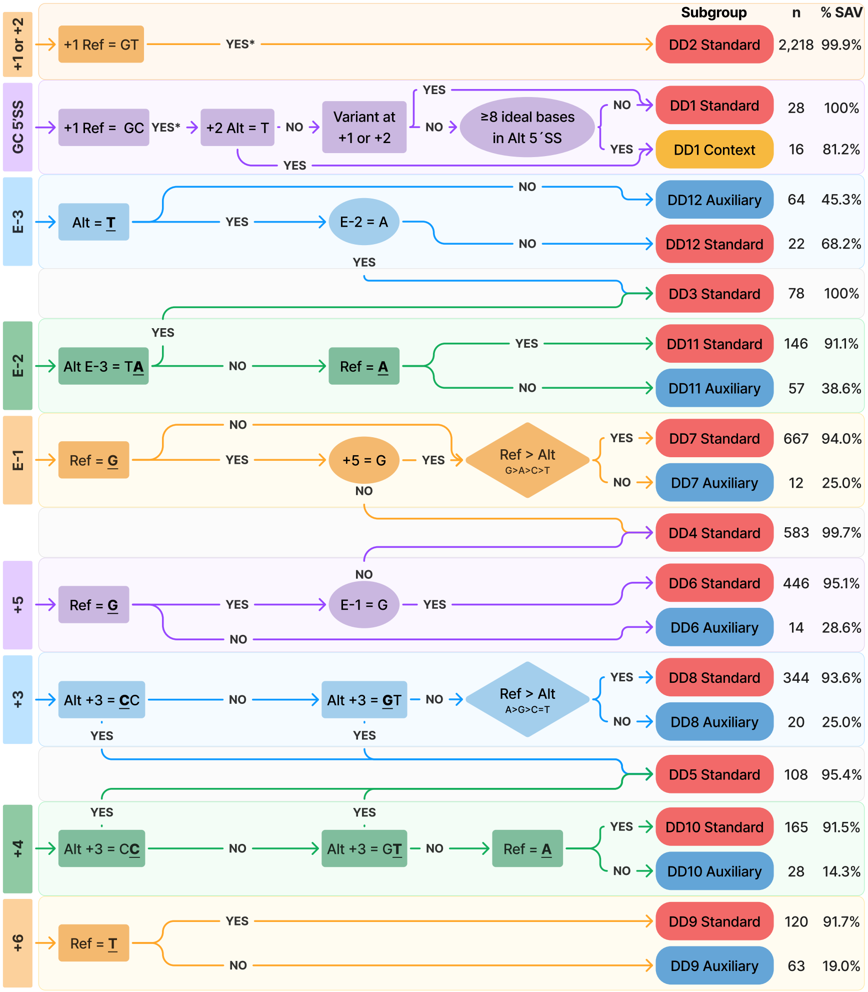


**Figure 3. Donor splice site disruption variant curation flowchart**

The flowchart starts on the left, where variant locations are categorized as affecting a canonical GT or rare GC donor splice site, the last three bases of an exon (E−3, E−2, and E−1), or the intronic bases of the donor splice region (+3 to +6). Each curation step follows a detailed decision pathway to determine the likelihood of a variant impacting splicing (spliceogenicity, abbreviated as %SAV). Rectangular nodes represent decision points concerning the attributes of the variant itself (variant nucleotides are bolded and underlined). Elliptical nodes indicate decisions related to other splicing elements not at the variant site. Diamond nodes denote base cascade rules, prioritizing specific nucleotide bases within a given motif. Variants from the SpliceVarDB ($n = 5{,}104$) are categorized using the heuristics depicted in this figure, resulting in subgroups with differing levels of spliceogenicity. Ref, reference nucleotide(s); Alt, variant nucleotide(s).

the SpliceVarDB affected splicing, whereas four of eleven C>T variants did alter splicing. Therefore, we redefined the order of preference in these heuristics to G>A>C>T at the last base of an exon. These nucleotide preferences are referred to as "base cascade rules" in the flowcharts (Figures 4 and 3). If the reference nucleotide is more preferred than the alternative nucleotide (for example, an A>T variant), then the variants are classified as "DD7 standard" with 94.0% [90.7–97.3] spliceogenicity ($n$ = 667). Variants where the reference nucleotide is less preferred (for example, an A>G variant) are classified as "DD7 auxiliary" and have a lower spliceogenicity (25.0% [0.7–49.3]; $n$ = 12).

***Third intronic base of the 5′SS (+3)***

For the fourth example, we consider a variant at the +3 position of the 5′SS. What is not obvious from the sequence logo in Figure 1A is that there is a marked depletion of CC and GT dinucleotides at the +3 and +4 positions. Following the flowchart in Figure 3, variants that create such combinations at +3 will be classified as "DD5 standard" (spliceogenicity 95.4% [87.3–100]; $n$ = 108). However, if a variant at the +3 site does not create a CC or GT dinucleotide, then the heuristics consider whether the variant is more or less preferred than the reference nucleotide, which we have determined to be A>G>C=T, with C and T being equally unfavorable. Variants at the +3 position with a less preferred alternative nucleotide (for example, a C) are classified as "DD8 standard" (spliceogenicity 93.6% [89.1–98.1]; $n$ = 344). Variants where the alternative nucleotide is more preferred (for example, an A) are classified as "DD8 auxiliary" (spliceogenicity 25.0% [6.2–43.8]; $n$ = 20).

All twelve donor disruption classification heuristics can be found in the DD flowchart (Figure 3), with additional explanations and splicing outcome insights found in the handbook (Data S1).

**Disruption of the 3′SS**

Unlike the 5′SS, recognition of the 3′SS relies on splicing elements outside of the acceptor motif itself. Variants affecting the acceptor motif, the branchpoint, and the PPT are considered in the 3′SS disruption splicing heuristics below, as they are all necessary for 3′SS selection. Of the 6,764 experimentally validated variants at the 3′SS, 61.1% ($n$ = 4,134) were SAVs. Applying the splicing requirement checklist to variants at the 3′SS revealed that 52% ($n$ = 2,151) render the original splice site unusable and 46% ($n$ = 1,919) weaken the original motif but leave it functional. The remaining SAVs ($n$ = 64) were anticipated, based on the splicing criteria, to strengthen the 3′SS. Here, we have translated our insights into heuristics to inform whether a variant at an established 3′SS is an SAV (Figure 4).

Building on the basic concepts covered by the examples for the 5′SS disruption (DD) heuristics, these examples will

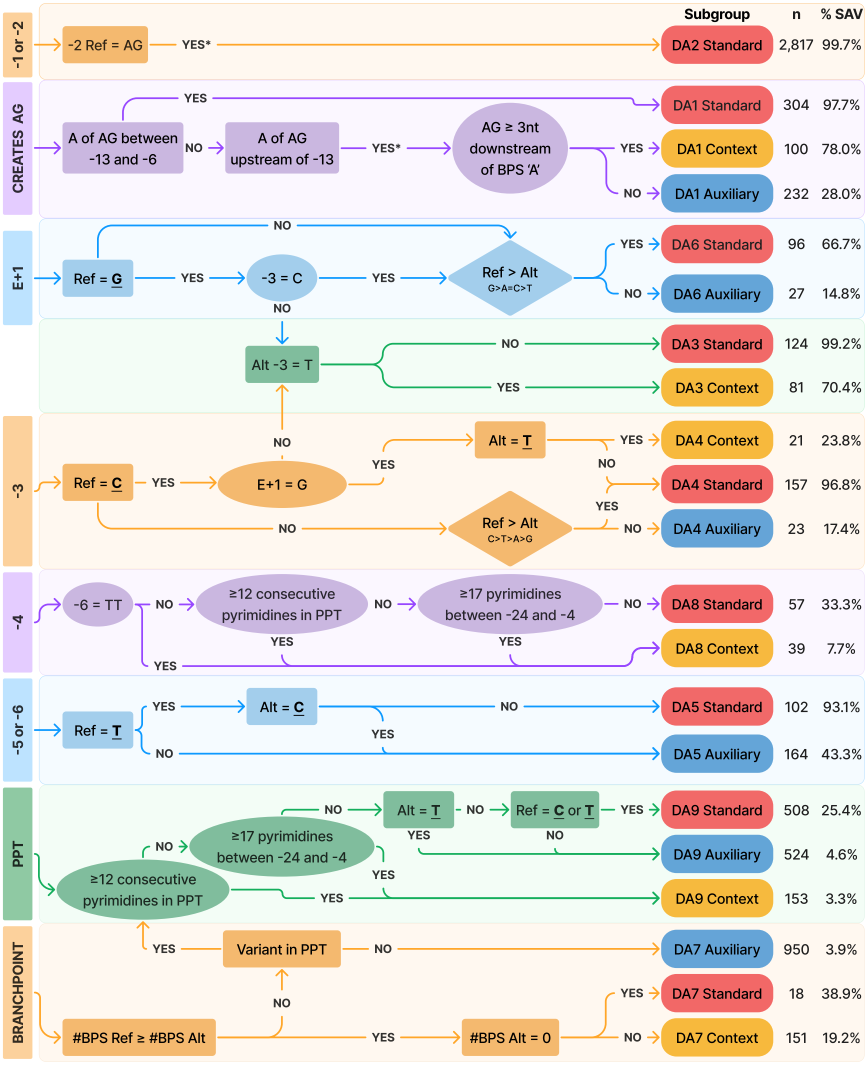


**Figure 4. Acceptor splice site disruption variant curation flowchart**

The flowchart starts on the left, with variant locations categorized as affecting the canonical AG dinucleotides, the first base of the exon (E+1), −3 to −6, the polypyrimidine tract (PPT), or the branchpoint. Each curation step follows a detailed decision pathway to determine the likelihood of a variant impacting splicing (spliceogenicity, abbreviated as % SAV). Rectangular nodes represent decision points concerning the attributes of the variant itself (variant nucleotides are bolded and underlined). Elliptical nodes indicate decisions related to other splicing elements not at the variant site. Diamond nodes denote base cascade rules, prioritizing specific nucleotide bases within a given motif. Variants from the SpliceVarDB ($n = 6{,}764$) are categorized using the heuristics depicted in this figure, resulting in subgroups with differing levels of spliceogenicity. Ref, reference nucleotide(s); Alt, variant nucleotide(s); *, only "YES" is considered, variants outside of this location are not covered by this heuristic; #BPS, number of sequences matching the branchpoint motif YNA.

walk through some of the more complex 3′SS disruption (DA) heuristics. Similar to the 5′SS disruption heuristics, no additional steps are needed to classify variants affecting the invariant AG dinucleotide of the 3′SS, categorized as "DA2 standard" with a spliceogenicity of 99.7% [98.1–100] ($n = 2{,}817$).

### *First base of the exon (E+1)*

The exonic bases adjacent to the splice site play less of a role in 3′SS selection than 5′SS selection; however, there is greater weight on the first base of the exon (E+1) for determining the location of the 3′SS. We determined the preference order at the first base to be G>A=C>T, where A or C was interchangeable in terms of resultant splice site strength. Following the acceptor flowchart, a variant with the reference E+1G is classified as "DA6 standard" with 66.7% [58.1–75.3] spliceogenicity ($n = 96$). However, recognition of the acceptor motif also depends on the −3 position. The spliceogenicity of an E+1G variant may be upgraded by considering the surrounding sequence context. If the nucleotide at −3 is not a pyrimidine, then "DA3 standard" is applicable with 99.2% [91.6–100] spliceogenicity ($n = 124$). We note that variants at the first base of the exon are highly relevant for an established splicing theory of AG-dependent exons, which have a G as the first exonic base. This theory proposed that AG-dependent exons do not require a long PPT to recognize the 3′SS.[40] However, in our analyses, including PPT length or strength as a contextual modifier did not assist in the classification of E+1 variants.

### *PPT variants (−24 to −5)*

Evaluating the effect of a variant in the polypyrimidine tract (PPT) is challenging because it is often unclear whether a minor modification to a larger motif can lead to an appreciable impact on splicing. Compared to other 3′SS disruption heuristics, there are a limited number of SAVs that fall under the DA9 heuristic (SAV $n = 146$; normal $n = 1{,}039$), despite the motif covering a large window. The acceptor disruption heuristics were unable to pinpoint which PPT variants cause splicing alterations, consistent with a spliceogenicity of at most 25.4%. Nevertheless, in this case, the utility of the heuristic comes from suggesting which variants in this region are unlikely to alter splicing, for example, exons preceded by a strong or long PPT classified as "DA9 context" with a spliceogenicity of only 3.3% [0–10.1] ($n = 153$). We defined a strong PPT as 12 or more consecutive pyrimidines (Ts or Cs) or 17 total pyrimidines between −4 and −24 (inclusive). Variants introducing the preferred nucleotide T to the PPT, or a variant that affects a non-pyrimidine in the PPT region, are classified as "DA9

auxiliary" (spliceogenicity 4.6% [0.9–8.3]; $n = 524$). The remaining variants are classified as "DA9 standard" with a spliceogenicity of 25.4% [21.7–29.1] ($n = 508$).

***Creation of additional AG dinucleotides upstream of the canonical 3′SS***

Due to the relatively broad sequence region of the 3′SS and associated motifs, there is a sensitivity to additional AG dinucleotides upstream of the canonical splice site. If an AG dinucleotide is created between the −13 and −6 positions (indexing on the A), then the variant is categorized as "DA1 standard" with a spliceogenicity of 97.7% [92.9–100] ($n = 304$). This region between the branchpoint and the 3′SS is known as the AGEZ.[14,31] The DA1 standard rule encompasses variants with high spliceogenicity,[34] regardless of the branchpoint location. In line with the standard AGEZ definition, "DA1 context" captures variants that create an AG dinucleotide at least 3 nt downstream of the closest branchpoint A (e.g., YNANNAG) but upstream of the −13 position captured in DA1 standard. Here, we define the BPS as a permissive sequence of TNA, although, as noted in splicing requirements, we have observed a high degree of sequence heterogeneity at the branchpoint. As a result, variants in "DA1 context" had a slightly reduced spliceogenicity of 78.0% [69.6–86.4] ($n = 100$), likely due to the highly degenerate branchpoint motifs often encountered. AG-creating variants that do not fall in either subgroup are classified as "DA1 auxiliary" and are less likely to be splice-altering (spliceogenicity of 28.0% [22.5–33.5]; $n = 232$) unless the new 3′SS satisfies the splicing requirements and outcompetes the existing 3′SS.

All acceptor disruption classification heuristics can be found in the DA flowchart (Figure 4). Additional literature details, confidence intervals, splicing outcomes, and further walkthroughs of all heuristics are available in the handbook (Data S1).

### Deep intronic variants and the creation of pseudoexons

Identifying variants that create novel splice sites is more challenging than evaluating damage to annotated splice sites. This is because successful splicing depends on the sequence context, with motifs present in a precise order with correct spacing and sufficient strength. While our splice disruption heuristics can indicate whether a variant will disrupt existing splice sites, our splicing checklists (Figure 1) help the investigator assess whether a deep intronic variant may lead to the creation of new splice sites or the activation of cryptic splice motifs. In this study, we apply these checklists to variants from the SpliceVarDB identified as creating a pseudoexon, aiming to uncover the requisite sequence features associated with pseudoexon inclusion.

A pseudoexon, or a poison exon, is normally an intronic segment that becomes recognized by the spliceosome and is included in the mRNA as an exon due to a deep intronic SAV.[27] The alteration of just one base can be enough to result in pseudoexon inclusion, implying that most of the sequence elements required for an exon to be recognized are already present, albeit with suboptimal motif strength to have been recognized. Pseudoexons are predominantly deleterious, as they often introduce a premature termination codon to the transcript through a frameshift or the inclusion of a stop codon in the newly included intronic sequence.

In the SpliceVarDB, 167 deep intronic variants were reported by their original publications to create a pseudoexon, usually through the creation of canonical 5′SS GT (36.5%; $n = 61$) or 3′SS AG (13.8%; $n = 23$) dinucleotides (Figure 5). An additional 44 variants (26.0%) occurred at a non-canonical base and strengthened a pre-existing, yet unutilized, 3′SS or 5′SS (Figure 5, "candidate site in Ref & Alt"). Twenty-two (13.1%) variants created pseudoexons through a variety of other mechanisms, including strengthening the PPT and removing an AG from an AGEZ (Figure 5, "candidate site only in Alt"). For the remaining variants ($n = 17$; 12.0%), we could not initially determine using our checklist why they had led to a pseudoexon (Figure 5, "no candidate sites in Alt"). Eleven of these variants were reported to create or disrupt a splicing enhancer and/or silencer motif,[41–50] which is not part of our checklist. The remaining variants appeared to promote the use of unsatisfactory splice sites ($n = 3$), insufficiently strengthened a splice site ($n = 2$), or were outside the typical window to affect a nearby 5′SS ($n = 1$).

Predicting whether a deep intronic SAV creates a pseudoexon involves finding nearby cryptic splice sites that could pair with it. By applying our splicing criteria (Figure 1C) to the sequence around the variant (see material and methods), we located, on average, three suitable cryptic splice sites for each variant capable of generating a pseudoexon, including 82% of those confirmed by experimental evidence.

### SREs

Core splicing elements, such as the 3′SS and 5′SS, are considered the primary determinants of splicing location. However, exon inclusion can be influenced by splicing regularory element variants (SREs), although how this is achieved remains poorly understood at present.[52] The effect on splicing of variants that strengthen or damage SRE motifs remains challenging to predict and interpret accurately, due in part to a lack of *bona fide* examples in the literature and SpliceVarDB.

We propose that the traditional view of SREs acting solely as enhancers or silencers of splicing is oversimplified. In reality, the impact of splicing regulatory proteins (SRPs) that bind to SREs depends on their relative abundance, competitive binding ability, and position relative to the exon, often with context-dependent effects on splicing.[53] Such effects include improving the recognition of splice sites that are typically considered weak,[54] determining splice site selection when multiple usable motifs exist,[55] and moderating tissue-specific alternative splicing.[56,57]

Examining SAVs that were not defined as splice altering by our heuristics revealed that some may disrupt or create potential SRE motifs. These overlooked changes to SREs could be the reason why some SAVs with a minimal impact

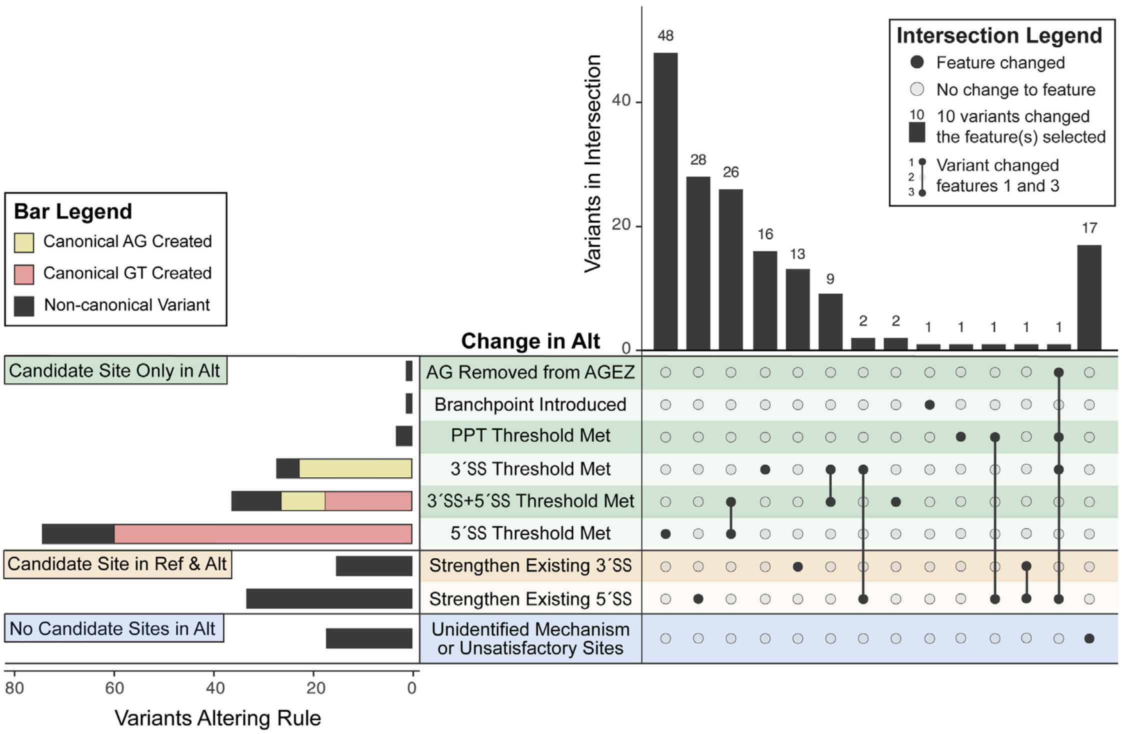


**Figure 5. Theoretical reconciliation of pseudoexon inclusion mechanisms**
UpSet[51] plot of the alterations made by deep intronic variants that promote the inclusion of pseudoexons, as determined by applying the splicing heuristics to the reference (Ref) and alternative (Alt) sequences. Alterations are divided into variants that were deemed by the splicing requirements (see splicing requirements) to create a usable splice site not present in the reference sequence (green), variants that strengthened a cryptic splice site that pre-existed in the reference sequence (orange), and variants where the mechanism of pseudoexon alteration could not be determined using the requirements (blue). The number of variants that matched each feature change is shown in the left horizontal bar plot, where variants are colored according to whether the variant created a canonical AG in a candidate 3′SS (yellow) or a canonical GT in a candidate 5′SS (red) or altered a splicing motif at a non-canonical nucleotide (black).

on the primary splice elements are not flagged by the heuristics. In one example, a variant in *CAPN3* (c.1187A>G [GenBank: NM_000070.2] [p.Gln396Gly]) created an SRE motif that overlapped the 5′SS, blocking the spliceosome from recognizing the original 5′SS, causing the exon to extend.[58] As mentioned above, we observed eleven deep intronic variants that created an SRE. Taking a deeper look at one of these, a deep intronic variant in *FGB* (c.115-600A>G [GenBank: NM_005141.2]) simultaneously damaged a silencer motif and strengthened an enhancer motif, which activated spliceosomal recognition of pre-existing requisite splice signals and resulted in the formation of a pseudoexon that included the SNV.[41]

Through our development of Introme, we observed that none of the current *in silico* splice prediction tools accurately identified SAVs that affect SREs.[59] Although databases and online tools for annotating SRP sites have been developed,[60,61] they do not fully capture the intricate interplay between different types of SREs, the variable expression of SRPs, and how these factors collectively influence splicing outcomes.

## Quantifying different splicing outcomes

The splicing outcome of a given variant critically depends on the sequence context surrounding the original exon and the potential compensation by nearby usable splice sites. In this section, we note some of the general trends for variants occurring in the splicing regions of known exons (Figure 6A), with the spliceogenicity of each splicing outcome for specific heuristics detailed in the handbook (Data S1). We observed multiple splicing outcomes for 19.1% of variants, which were predominantly exon skipping (71.3%) or intron retention (44.2%) alongside an additional outcome.

Exon skipping was identified as the most prevalent splicing outcome, observed for 51.3% of all variants in known splicing regions. The remaining alterations were exon truncation for 36.3%, intron retention for 17.4%, and exon extension for 16.2% of the variants. Notably, the majority of cryptic splice sites utilized were in close proximity (less than 100 nt) to the original splice site, with 93.3% of cryptic intronic sites and 86.8% of cryptic exonic sites falling within this range.

Variants that affected splicing upstream of the 3′SS canonical AG motif were more likely to cause an upstream intronic cryptic splice site to be used than a downstream exonic splice site (43% vs. 20%), thereby resulting in exon extension. The opposite was true for variants that affect the first base of an exon or canonical 3′SS, where the use of a downstream exonic splice site is much more

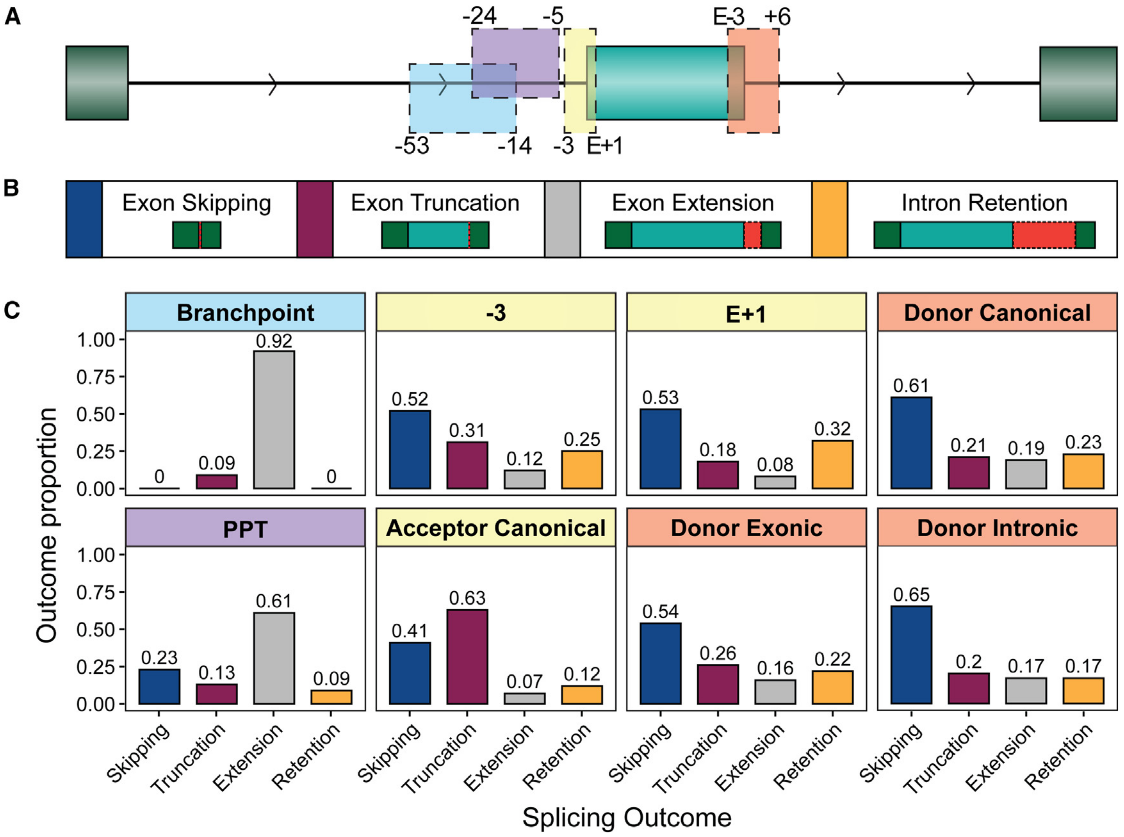


**Figure 6. Splicing outcomes by variant location**
(A) Green filled boxes represent exons, with the exon impacted by the variant colored a lighter green. Black lines with arrows represent the introns and the direction (5′ to 3′). Colored boxes correspond to the locations of relevant motifs, with the flanking numbers demarcating sequence windows in relation to the exon: branchpoint region (blue), polypyrimidine tract (purple), acceptor motif (yellow), and donor splice site (orange).
(B) Depictions of the observed splicing outcomes: exon skipping, exon truncation, exon extension, and full intron retention. Red dotted lines or boxes show the impact of the aberrant splicing outcome on the transcript (red lines: removed exonic sequence; red boxes: inserted intronic sequence). Colored boxes are used as the legend for the bar plots in (C).
(C) The splicing outcome proportions observed for variants that are splice altering, faceted by splicing regions. Multiple splicing outcomes can occur for a given variant; thus, the proportions do not sum to 100%. PPT, polypyrimidine tract.

likely than an upstream intronic cryptic splice site (62% vs. 7%), thereby resulting in exon truncation. This observation aligns with the scanning 3′SS selection model, where the spliceosome primarily recognizes and utilizes the first appropriate AG motif downstream of the branchpoint.[15,32]

A higher incidence of intron retention was observed at the 5′SS as opposed to the 3′SS, with occurrences of 25% and 13%, respectively. This trend was accentuated when considering cases where only one splicing outcome was reported (5′SS: 16%; 3′SS: 5%). This suggests that a damaged 5′SS is more likely to be the cause of full intron retention.

## Discussion

The splicing heuristics presented here are data driven and grounded in biology to support the interpretation of SAVs. By analyzing annotated exons and experimentally validated SAVs, we provide insight, clarity, and understanding of the effects of SAVs. This study provides a quantitative assessment of spliceogenicity for variants in particular contexts. While recognizing the value of *in silico* prediction tools for identifying SAVs, we aim to provide additional context to aid in the interpretation and pathogenicity assessment of SAVs. This work contributes to bridging the gap between *in silico* predictive capability and the clinical classification and interpretation of variants.

The effectiveness of these heuristics is inherently tied to the scope of genetic variants considered, making them limited by current understanding and available data. We encourage user contribution of functionally validated variants to the SpliceVarDB (https://splicevardb.org)[19] to expand the database. Continuous advancements in this field will enable the ongoing refinement of these heuristics and the *in silico* tools that may utilize them. While there is an ascertainment bias in the literature, with non-SAVs being underreported, high-throughput splicing assays may address this issue. Indeed, several such studies have already been incorporated into the SpliceVarDB.[62–64] The quality of the experimental data is crucial, as the reliability of our findings depends on the accuracy of the experimental

validation. Some *in vitro* assays, such as those measuring exon inclusion rates, may not detect altered splice site utilization, leading to partial exon extension or intron retention. Additionally, the context-dependent nature of splicing means that validation results may vary based on the experimental cellular system or the patient-derived tissue used.

While one of our initial objectives was to improve predictions of transcript alterations associated with SAVs, this study has revealed significant heterogeneity in splicing outcomes (Figure 6). The genetic context in which an SAV occurs significantly impacts the functional outcome(s) of the variant. Certain regions are more susceptible to specific mis-splicing patterns, highly influenced by the presence of cryptic splice sites and regulatory elements. Resources that catalog novel splice junctions, like SpliceVault[65] and OncoSplicing,[66] are valuable for understanding the mis-splicing likely to be associated with a region.

A notable constraint of these heuristics is their inability to account for edge cases within the complex splicing landscape, which is especially true for genetic variants affecting SREs.[59] By excluding SREs from our analysis, these heuristics only consider the core components of the spliceosome, effectively making our approach tissue independent. Additional multi-consideration splicing theories, such as intron vs. exon definition,[67,68] RNA secondary structure,[69] methylation,[70,71] and splicing order,[72] were considered in our analysis but ultimately not utilized in these heuristics due to limited examples of relevant variants. Further consideration of these and other unresolved mechanisms (e.g., splicing regulatory landscape, tissue specificity, and branchpoint location) as data become available will lead to improvements in these heuristics at the potential risk of adding further complexity.

In developing these splicing heuristics, we aimed to introduce heuristic subgroups that maximized spliceogenicity values within specific sequence contexts, ensuring there were enough example variants to avoid overfitting. Our objective was to create heuristics that seasoned practitioners could easily follow despite the inherent complexity of splicing biology. A future opportunity would be the development of a bioinformatic tool that implements these heuristics, enhancing their uptake and supporting high-throughput SAV curation. Such a tool would then permit the systematic benchmarking of these heuristics, ultimately determining their predictive value. Through intentional integration of empirical evidence into the development of such tools, we can mitigate the limitations faced by powerful deep learning algorithms that have, to date, been narrowly trained on naturally occurring splice junctions and relatively few experimentally confirmed SAVs. Reinforcing the predictive capabilities of *in silico* tools with robust, empirically validated heuristics will more closely align computational predictions with the functional impacts of SAVs for improved accuracy.

These data-driven splicing heuristics lay a solid foundation for interpreting SAVs, offering a biologically grounded approach that supports the curation scientist to interpret candidate SAVs identified by existing *in silico* prediction tools. In the long term, we hope that they will serve as essential building blocks for creating biologically informed *in silico* prediction tools. This approach guarantees transparency, multifactorial consideration, and the effective translation of known splicing biology into predictive models. By integrating insights from large numbers of experimentally validated SAVs, we aim to improve the precision of SAV interpretation, underpin the advancement of personalized medicine, and deepen our understanding of genetic disease mechanisms associated with SAVs.

## Data and code availability

Original source data for figures in the paper is available at https://github.com/CCICB/SplicingHeuristics2025.

## Acknowledgments

We thank Kimberley Dias, Paulette Barahona, Dr. Dianne Sylvester, Dr. Noemi Fuentes-Bolanos, and Chelsea Mayoh for helpful discussions about SAV curation. We thank Dr. Amali Mallawaarachchi and Dr. Yvonne Hort for the *PKD1* splicing example. We thank Prof. Heidi Rehm for helpful discussions about positioning our heuristics.

P.J.S. was supported by an Australian government Research Training Program (RTP) scholarship, the Kids Cancer Alliance PhD Top Up scholarship, the Petre Foundation PhD Top Up scholarship, and a Fulbright Future scholarship. M.P. is supported by an NHMRC Investigator Grant (#1176265). R.L.D. was supported by an NSW Health Early-Mid Career Fellowship. M.J.C. was supported by an NSW Health Early-Mid Career Fellowship and the Medical Research Future Fund (MRFF) Emerging Priorities and Consumer-Driven Research initiative. This project was supported by grant 1165556 awarded through the 2018 Priority-driven Collaborative Cancer Research Scheme and co-funded by Cancer Australia and My Room. The authors would like to acknowledge Luminesce Alliance – Innovation for Children's Health for its contribution and support. Luminesce Alliance is a not-for-profit cooperative joint venture between the Sydney Children's Hospitals Network, the Children's Medical Research Institute, and the Children's Cancer Institute. It has been established with the support of the NSW government to coordinate and integrate pediatric research. Luminesce Alliance is also affiliated with the University of Sydney and the University of New South Wales Sydney.

## Author contributions

P.J.S. analyzed the variants, determined the splicing requirements, and developed the splicing rules. P.A. provided variant curation expertise. P.J.S. and M.J.C. conceptualized the study. P.J.S., J.M.W.Q., M.P., R.L.D., and M.J.C. wrote the paper. M.P., R.L.D., and M.J.C. supervised the work, and M.J.C. obtained funding for the study. All authors reviewed and approved the manuscript.

## Declaration of interests

The authors declare no competing interests.

## Declaration of generative AI and AI-assisted technologies in the writing process

During the preparation of this work, the authors used GPT-4 in order to refine the grammar in some parts of the manuscript. After using this tool, the authors reviewed and edited the content as needed and take full responsibility for the content of the publication.

## Supplemental information

Supplemental information can be found online at https://doi.org/10.1016/j.ajhg.2025.02.012.

## Web resources

GenBank, https://www.ncbi.nlm.nih.gov/genbank/
SpliceVarDB, http://splicevardb.org

Received: March 11, 2024
Accepted: February 11, 2025
Published: March 7, 2025

## References

1. Baralle, D., Lucassen, A., and Buratti, E. (2009). Missed threads: The impact of pre-mRNA splicing defects on clinical practice. EMBO Rep. *10*, 810–816. https://doi.org/10.1038/embor.2009.170.
2. Scotti, M.M., and Swanson, M.S. (2016). RNA mis-splicing in disease. Nat. Rev. Genet. *17*, 19–32. https://doi.org/10.1038/nrg.2015.3.
3. Kastner, B., Will, C.L., Stark, H., and Lührmann, R. (2019). Structural Insights into Nuclear pre-mRNA Splicing in Higher Eukaryotes. Cold Spring Harb. Perspect. Biol. *11*, a032417. https://doi.org/10.1101/cshperspect.a032417.
4. Baralle, M., and Baralle, F.E. (2018). The splicing code. Biosystems *164*, 39–48. https://doi.org/10.1016/j.biosystems.2017.11.002.
5. Wilkinson, M.E., Charenton, C., and Nagai, K. (2020). RNA Splicing by the Spliceosome. Annu. Rev. Biochem. *89*, 359–388. https://doi.org/10.1146/annurev-biochem-091719-064225.
6. Richards, S., Aziz, N., Bale, S., Bick, D., Das, S., Gastier-Foster, J., Grody, W.W., Hegde, M., Lyon, E., Spector, E., et al. (2015). Standards and guidelines for the interpretation of sequence variants: a joint consensus recommendation of the American College of Medical Genetics and Genomics and the Association for Molecular Pathology. Genet. Med. *17*, 405–424. https://doi.org/10.1038/gim.2015.30.
7. Abou Tayoun, A.N., Pesaran, T., DiStefano, M.T., Oza, A., Rehm, H.L., Biesecker, L.G., Harrison, S.M.; and ClinGen Sequence Variant Interpretation Working Group ClinGen SVI (2018). Recommendations for interpreting the loss of function PVS1 ACMG/AMP variant criterion. Hum. Mutat. *39*, 1517–1524. https://doi.org/10.1002/humu.23626.
8. Walker, L.C., Hoya, M.d. l., Wiggins, G.A.R., Lindy, A., Vincent, L.M., Parsons, M.T., Canson, D.M., Bis-Brewer, D., Cass, A., Tchourbanov, A., et al. (2023). Using the ACMG/AMP framework to capture evidence related to predicted and observed impact on splicing: Recommendations from the ClinGen SVI Splicing Subgroup. Am. J. Hum. Genet. *110*, 1046–1067. https://doi.org/10.1016/j.ajhg.2023.06.002.
9. Ellingford, J.M., Ahn, J.W., Bagnall, R.D., Baralle, D., Barton, S., Campbell, C., Downes, K., Ellard, S., Duff-Farrier, C., FitzPatrick, D.R., et al. (2022). Recommendations for clinical interpretation of variants found in non-coding regions of the genome. Genome Med. *14*, 73. https://doi.org/10.1186/s13073-022-01073-3.
10. Zeng, T., and Li, Y.I. (2022). Predicting RNA splicing from DNA sequence using Pangolin. Genome Biol. *23*, 103. https://doi.org/10.1186/s13059-022-02664-4.
11. Jaganathan, K., Kyriazopoulou Panagiotopoulou, S., McRae, J.F., Darbandi, S.F., Knowles, D., Li, Y.I., Kosmicki, J.A., Arbelaez, J., Cui, W., Schwartz, G.B., et al. (2019). Predicting Splicing from Primary Sequence with Deep Learning. Cell *176*, 535–548.e24. https://doi.org/10.1016/j.cell.2018.12.015.
12. Hort, Y., Sullivan, P., Wedd, L., Fowles, L., Stevanovski, I., Deveson, I., Simons, C., Mallett, A., Patel, C., Furlong, T., et al. (2023). Atypical splicing variants in PKD1 explain most undiagnosed typical familial ADPKD. npj NPJ Genom. Med. *8*, 16. https://doi.org/10.1038/s41525-023-00362-z.
13. Zhang, P., Chaldebas, M., Ogishi, M., Al Qureshah, F., Ponsin, K., Feng, Y., Rinchai, D., Milisavljevic, B., Han, J.E., Moncada-Vélez, M., et al. (2023). Genome-wide detection of human intronic AG-gain variants located between splicing branchpoints and canonical splice acceptor sites. Proc. Natl. Acad. Sci. USA *120*, e2314225120. https://doi.org/10.1073/pnas.2314225120.
14. Gooding, C., Clark, F., Wollerton, M.C., Grellscheid, S.-N., Groom, H., and Smith, C.W.J. (2006). A class of human exons with predicted distant branch points revealed by analysis of AG dinucleotide exclusion zones. Genome Biol. *7*, R1. https://doi.org/10.1186/gb-2006-7-1-r1.
15. Smith, C.W., Chu, T.T., and Nadal-Ginard, B. (1993). Scanning and competition between AGs are involved in 3′ splice site selection in mammalian introns. Mol. Cell Biol. *13*, 4939–4952. https://doi.org/10.1128/MCB.13.8.4939.
16. Barash, Y., Calarco, J.A., Gao, W., Pan, Q., Wang, X., Shai, O., Blencowe, B.J., and Frey, B.J. (2010). Deciphering the splicing code. Nature *465*, 53–59. https://doi.org/10.1038/nature09000.
17. Ward, A.J., and Cooper, T.A. (2010). The pathobiology of splicing. J. Pathol. *220*, 152–163. https://doi.org/10.1002/path.2649.
18. Wachutka, L., Caizzi, L., Gagneur, J., and Cramer, P. (2019). Global donor and acceptor splicing site kinetics in human cells. Elife *8*, e45056. https://doi.org/10.7554/eLife.45056.
19. Sullivan, P.J., Quinn, J.M.W., Wu, W., Pinese, M., and Cowley, M.J. (2024). SpliceVarDB: A comprehensive database of experimentally validated human splicing variants. Am. J. Hum. Genet. *111*, 2164–2175. https://doi.org/10.1016/j.ajhg.2024.08.002.
20. Frankish, A., Diekhans, M., Jungreis, I., Lagarde, J., Loveland, J.E., Mudge, J.M., Sisu, C., Wright, J.C., Armstrong, J., Barnes, I., et al. (2021). GENCODE 2021. Nucleic Acids Res. *49*, D916–D923. https://doi.org/10.1093/nar/gkaa1087.
21. Quinlan, A.R., and Hall, I.M. (2010). BEDTools: a flexible suite of utilities for comparing genomic features. Bioinformatics *26*, 841–842. https://doi.org/10.1093/bioinformatics/btq033.
22. Danecek, P., Bonfield, J.K., Liddle, J., Marshall, J., Ohan, V., Pollard, M.O., Whitwham, A., Keane, T., McCarthy, S.A.,

Davies, R.M., and Li, H. (2021). Twelve years of SAMtools and BCFtools. GigaScience *10*, giab008. https://doi.org/10.1093/gigascience/giab008.
23. Moyer, D.C., Larue, G.E., Hershberger, C.E., Roy, S.W., and Padgett, R.A. (2020). Comprehensive database and evolutionary dynamics of U12-type introns. Nucleic Acids Res. *48*, 7066–7078. https://doi.org/10.1093/nar/gkaa464.
24. Mercer, T.R., Clark, M.B., Andersen, S.B., Brunck, M.E., Haerty, W., Crawford, J., Taft, R.J., Nielsen, L.K., Dinger, M.E., and Mattick, J.S. (2015). Genome-wide discovery of human splicing branchpoints. Genome Res. *25*, 290–303. https://doi.org/10.1101/gr.182899.114.
25. Wagih, O. (2017). ggseqlogo: a versatile R package for drawing sequence logos. Bioinformatics *33*, 3645–3647. https://doi.org/10.1093/bioinformatics/btx469.
26. Yeo, G., and Burge, C.B. (2004). Maximum Entropy Modeling of Short Sequence Motifs with Applications to RNA Splicing Signals. J. Comput. Biol. *11*, 377–394. https://doi.org/10.1089/1066527041410418.
27. Keegan, N.P., Wilton, S.D., and Fletcher, S. (2021). Analysis of Pathogenic Pseudoexons Reveals Novel Mechanisms Driving Cryptic Splicing. Front. Genet. *12*, 806946. https://doi.org/10.3389/fgene.2021.806946.
28. Sakaguchi, N., and Suyama, M. (2021). In silico identification of pseudo-exon activation events in personal genome and transcriptome data. RNA Biol. *18*, 382–390. https://doi.org/10.1080/15476286.2020.1809195.
29. Turunen, J.J., Niemelä, E.H., Verma, B., and Frilander, M.J. (2013). The significant other: splicing by the minor spliceosome. WIREs RNA *4*, 61–76. https://doi.org/10.1002/wrna.1141.
30. Yıldırım, B., and Vogl, C. (2023). Purifying selection against spurious splicing signals contributes to the base composition evolution of the polypyrimidine tract. J. Evol. Biol. *36*, 1295–1312. https://doi.org/10.1111/jeb.14205.
31. Wimmer, K., Schamschula, E., Wernstedt, A., Traunfellner, P., Amberger, A., Zschocke, J., Kroisel, P., Chen, Y., Callens, T., and Messiaen, L. (2020). AG-exclusion zone revisited: Lessons to learn from 91 intronic NF1 3 splice site mutations outside the canonical AG-dinucleotides. Hum. Mutat. *41*, 1145–1156. https://doi.org/10.1002/humu.24005.
32. Chua, K., and Reed, R. (2001). An Upstream AG Determines Whether a Downstream AG Is Selected during Catalytic Step II of Splicing. Mol. Cell Biol. *21*, 1509–1514. https://doi.org/10.1128/MCB.21.5.1509-1514.2001.
33. Sohail, M., and Xie, J. (2015). Diverse regulation of 3 splice site usage. Cell. Mol. Life Sci. *72*, 4771–4793. https://doi.org/10.1007/s00018-015-2037-5.
34. Bryen, S.J., Joshi, H., Evesson, F.J., Girard, C., Ghaoui, R., Waddell, L.B., Testa, A.C., Cummings, B., Arbuckle, S., Graf, N., et al. (2019). Pathogenic Abnormal Splicing Due to Intronic Deletions that Induce Biophysical Space Constraint for Spliceosome Assembly. Am. J. Hum. Genet. *105*, 573–587. https://doi.org/10.1016/j.ajhg.2019.07.013.
35. Zhang, K.Y., Joshi, H., Marchant, R.G., Bryen, S.J., Dawes, R., Yuen, M., Cooper, S.T., and Evesson, F.J. (2024). Refining clinically relevant parameters for mis-splicing risk in shortened introns with donor-to-branchpoint space constraint. Eur. J. Hum. Genet. *32*, 972–979. https://doi.org/10.1038/s41431-024-01632-9.
36. Signal, B., Gloss, B.S., Dinger, M.E., and Mercer, T.R. (2018). Machine learning annotation of human branchpoints. Bioinformatics *34*, 920–927. https://doi.org/10.1093/bioinformatics/btx688.
37. Leman, R., Tubeuf, H., Raad, S., Tournier, I., Derambure, C., Lanos, R., Gaildrat, P., Castelain, G., Hauchard, J., Killian, A., et al. (2020). Assessment of branch point prediction tools to predict physiological branch points and their alteration by variants. BMC Genom. *21*, 86. https://doi.org/10.1186/s12864-020-6484-5.
38. McLaren, W., Gil, L., Hunt, S.E., Riat, H.S., Ritchie, G.R.S., Thormann, A., Flicek, P., and Cunningham, F. (2016). The Ensembl Variant Effect Predictor. Genome Biol. *17*, 122. https://doi.org/10.1186/s13059-016-0974-4.
39. Aoto, Y., Horinouchi, T., Yamamura, T., Kondo, A., Nagai, S., Ishiko, S., Okada, E., Rossanti, R., Sakakibara, N., Nagano, C., et al. (2022). Last Nucleotide Substitutions of COL4A5 Exons Cause Aberrant Splicing. Kidney Int. Rep. *7*, 108–116. https://doi.org/10.1016/j.ekir.2021.10.012.
40. Fu, Y., Masuda, A., Ito, M., Shinmi, J., and Ohno, K. (2011). AG-dependent 3-splice sites are predisposed to aberrant splicing due to a mutation at the first nucleotide of an exon. Nucleic Acids Res. *39*, 4396–4404. https://doi.org/10.1093/nar/gkr026.
41. Davis, R.L., Homer, V.M., George, P.M., and Brennan, S.O. (2009). A deep intronic mutation in FGB creates a consensus exonic splicing enhancer motif that results in afibrinogenemia caused by aberrant mRNA splicing, which can be corrected in vitro with antisense oligonucleotide treatment. Hum. Mutat. *30*, 221–227. https://doi.org/10.1002/humu.20839.
42. Sangermano, R., Garanto, A., Khan, M., Runhart, E.H., Bauwens, M., Bax, N.M., van den Born, L.I., Khan, M.I., Cornelis, S.S., Verheij, J.B.G.M., et al. (2019). Deep-intronic ABCA4 variants explain missing heritability in Stargardt disease and allow correction of splice defects by antisense oligonucleotides. Genet. Med. *21*, 1751–1760. https://doi.org/10.1038/s41436-018-0414-9.
43. Bauwens, M., Garanto, A., Sangermano, R., Naessens, S., Weisschuh, N., De Zaeytijd, J., Khan, M., Sadler, F., Balikova, I., Van Cauwenbergh, C., et al. (2019). ABCA4-associated disease as a model for missing heritability in autosomal recessive disorders: novel noncoding splice, cis-regulatory, structural, and recurrent hypomorphic variants. Genet. Med. *21*, 1761–1771. https://doi.org/10.1038/s41436-018-0420-y.
44. Rius, R., Riley, L.G., Guo, Y., Menezes, M., Compton, A.G., Van Bergen, N.J., Gayevskiy, V., Cowley, M.J., Cummings, B.B., Adams, L., et al. (2019). Cryptic intronic NBAS variant reveals the genetic basis of recurrent liver failure in a child. Mol. Genet. Metab. *126*, 77–82. https://doi.org/10.1016/j.ymgme.2018.12.002.
45. Larrue, R., Chamley, P., Bardyn, T., Lionet, A., Gnemmi, V., Cauffiez, C., Glowacki, F., Pottier, N., and Broly, F. (2020). Diagnostic utility of whole-genome sequencing for nephronophthisis. npj NPJ Genom. Med. *5*, 38. https://doi.org/10.1038/s41525-020-00147-8.
46. Schalk, A., Greff, G., Drouot, N., Obringer, C., Dollfus, H., Laugel, V., Chelly, J., and Calmels, N. (2018). Deep intronic variation in splicing regulatory element of the ERCC8 gene associated with severe but long-term survival Cockayne syndrome. Eur. J. Hum. Genet. *26*, 527–536. https://doi.org/10.1038/s41431-017-0009-y.
47. Homolova, K., Zavadakova, P., Doktor, T.K., Schroeder, L.D., Kozich, V., and Andresen, B.S. (2010). The deep intronic

c.903+469T>C mutation in the MTRR gene creates an SF2/ASF binding exonic splicing enhancer, which leads to pseudoexon activation and causes the cblE type of homocystinuria. Hum. Mutat. *31*, 437–444. https://doi.org/10.1002/humu.21206.
48. Rincón, A., Aguado, C., Desviat, L., Sánchez-Alcudia, R., Ugarte, M., and Pérez, B. (2007). Propionic and Methylmalonic Acidemia: Antisense Therapeutics for Intronic Variations Causing Aberrantly Spliced Messenger RNA. Am. J. Hum. Genet. *81*, 1262–1270. https://doi.org/10.1086/522376.
49. Jin, M., Li, J.-J., Xu, G.-R., Wang, N., and Wang, Z.-Q. (2020). Cryptic exon activation causes dystrophinopathy in two Chinese families. Eur. J. Hum. Genet. *28*, 947–955. https://doi.org/10.1038/s41431-020-0578-z.
50. Trabelsi, M., Beugnet, C., Deburgrave, N., Commere, V., Orhant, L., Leturcq, F., and Chelly, J. (2014). When a mid-intronic variation of DMD gene creates an ESE site. Neuromuscul. Disord. *24*, 1111–1117. https://doi.org/10.1016/j.nmd.2014.07.003.
51. Conway, J.R., Lex, A., and Gehlenborg, N. (2017). UpSetR: an R package for the visualization of intersecting sets and their properties. Bioinformatics *33*, 2938–2940. https://doi.org/10.1093/bioinformatics/btx364.
52. Zhang, X.H.-F., Kangsamaksin, T., Chao, M.S.P., Banerjee, J.K., and Chasin, L.A. (2005). Exon Inclusion Is Dependent on Predictable Exonic Splicing Enhancers. Mol. Cell Biol. *25*, 7323–7332. https://doi.org/10.1128/MCB.25.16.7323-7332.2005.
53. Schmok, J.C., Jain, M., Street, L.A., Tankka, A.T., Schafer, D., Her, H.-L., Elmsaouri, S., Gosztyla, M.L., Boyle, E.A., Jagannatha, P., et al. (2024). Large-scale evaluation of the ability of RNA-binding proteins to activate exon inclusion. Nat. Biotechnol. *42*, 1429–1441. https://doi.org/10.1038/s41587-023-02014-0.
54. Wei, W.-J., Mu, S.-R., Heiner, M., Fu, X., Cao, L.-J., Gong, X.-F., Bindereif, A., and Hui, J. (2012). YB-1 binds to CAUC motifs and stimulates exon inclusion by enhancing the recruitment of U2AF to weak polypyrimidine tracts. Nucleic Acids Res. *40*, 8622–8636. https://doi.org/10.1093/nar/gks579.
55. Ptok, J., Müller, L., Theiss, S., and Schaal, H. (2019). Context matters: Regulation of splice donor usage. Biochim. Biophys. Acta. Gene Regul. Mech. *1862*, 194391. https://doi.org/10.1016/j.bbagrm.2019.06.002.
56. Liu, Q., Fang, L., and Wu, C. (2022). Alternative Splicing and Isoforms: From Mechanisms to Diseases. Genes *13*, 401. https://doi.org/10.3390/genes13030401.
57. Naro, C., Cesari, E., and Sette, C. (2021). Splicing regulation in brain and testis: common themes for highly specialized organs. Cell Cycle *20*, 480–489. https://doi.org/10.1080/15384101.2021.1889187.
58. Dionnet, E., Defour, A., Da Silva, N., Salvi, A., Lévy, N., Krahn, M., Bartoli, M., Puppo, F., and Gorokhova, S. (2020). Splicing impact of deep exonic missense variants in CAPN3 explored systematically by minigene functional assay. Hum. Mutat. *41*, 1797–1810. https://doi.org/10.1002/humu.24083.
59. Sullivan, P.J., Gayevskiy, V., Davis, R.L., Wong, M., Mayoh, C., Mallawaarachchi, A., Hort, Y., McCabe, M.J., Beecroft, S., Jackson, M.R., et al. (2023). Introme accurately predicts the impact of coding and noncoding variants on gene splicing, with clinical applications. Genome Biol. *24*, 118. https://doi.org/10.1186/s13059-023-02936-7.
60. Cartegni, L., Wang, J., Zhu, Z., Zhang, M.Q., and Krainer, A.R. (2003). ESEfinder: a web resource to identify exonic splicing enhancers. Nucleic Acids Res. *31*, 3568–3571. https://doi.org/10.1093/nar/gkg616.
61. Piva, F., Giulietti, M., Burini, A.B., and Principato, G. (2012). SpliceAid 2: A database of human splicing factors expression data and RNA target motifs. Hum. Mutat. *33*, 81–85. https://doi.org/10.1002/humu.21609.
62. Chong, R., Insigne, K.D., Yao, D., Burghard, C.P., Wang, J., Hsiao, Y.-H.E., Jones, E.M., Goodman, D.B., Xiao, X., and Kosuri, S. (2019). A Multiplexed Assay for Exon Recognition Reveals that an Unappreciated Fraction of Rare Genetic Variants Cause Large-Effect Splicing Disruptions. Mol. Cell *73*, 183–194.e8. https://doi.org/10.1016/j.molcel.2018.10.037.
63. Rhine, C.L., Neil, C., Glidden, D.T., Cygan, K.J., Fredericks, A.M., Wang, J., Walton, N.A., and Fairbrother, W.G. (2019). Future directions for high-throughput splicing assays in precision medicine. Hum. Mutat. *40*, 1225–1234. https://doi.org/10.1002/humu.23866.
64. Rhine, C.L., Neil, C., Wang, J., Maguire, S., Buerer, L., Salomon, M., Meremikwu, I.C., Kim, J., Strande, N.T., and Fairbrother, W.G. (2022). Massively parallel reporter assays discover de novo exonic splicing mutants in paralogs of Autism genes. PLoS Genet. *18*, e1009884. https://doi.org/10.1371/journal.pgen.1009884.
65. Dawes, R., Bournazos, A.M., Bryen, S.J., Bommireddipalli, S., Marchant, R.G., Joshi, H., and Cooper, S.T. (2023). SpliceVault predicts the precise nature of variant-associated mis-splicing. Nat. Genet. *55*, 324–332. https://doi.org/10.1038/s41588-022-01293-8.
66. Zhang, Y., Yao, X., Zhou, H., Wu, X., Tian, J., Zeng, J., Yan, L., Duan, C., Liu, H., Li, H., et al. (2022). OncoSplicing: an updated database for clinically relevant alternative splicing in 33 human cancers. Nucleic Acids Res. *50*, D1340–D1347. https://doi.org/10.1093/nar/gkab851.
67. De Conti, L., Baralle, M., and Buratti, E. (2013). Exon and intron definition in pre-mRNA splicing. WIREs RNA *4*, 49–60. https://doi.org/10.1002/wrna.1140.
68. Carranza, F., Shenasa, H., and Hertel, K.J. (2022). Splice site proximity influences alternative exon definition. RNA Biol. *19*, 829–840. https://doi.org/10.1080/15476286.2022.2089478.
69. Buratti, E., and Baralle, F.E. (2004). Influence of RNA Secondary Structure on the Pre-mRNA Splicing Process. Mol. Cell Biol. *24*, 10505–10514. https://doi.org/10.1128/MCB.24.24.10505-10514.2004.
70. Lev Maor, G., Yearim, A., and Ast, G. (2015). The alternative role of DNA methylation in splicing regulation. Trends Genet. *31*, 274–280. https://doi.org/10.1016/j.tig.2015.03.002.
71. Mendel, M., Delaney, K., Pandey, R.R., Chen, K.-M., Wenda, J.M., Vågbø, C.B., Steiner, F.A., Homolka, D., and Pillai, R.S. (2021). Splice site m6A methylation prevents binding of U2AF35 to inhibit RNA splicing. Cell *184*, 3125–3142.e25. https://doi.org/10.1016/j.cell.2021.03.062.
72. Choquet, K., Baxter-Koenigs, A.R., Dülk, S.-L., Smalec, B.M., Rouskin, S., and Churchman, L.S. (2023). Pre-mRNA splicing order is predetermined and maintains splicing fidelity across multi-intronic transcripts. Nat. Struct. Mol. Biol. *30*, 1064–1076. https://doi.org/10.1038/s41594-023-01035-2.

The American Journal of Human Genetics, Volume *112*

# Supplemental information

# Data-driven insights to inform splice-altering variant assessment

**Patricia J. Sullivan, Julian M.W. Quinn, Pamela Ajuyah, Mark Pinese, Ryan L. Davis, and Mark J. Cowley**

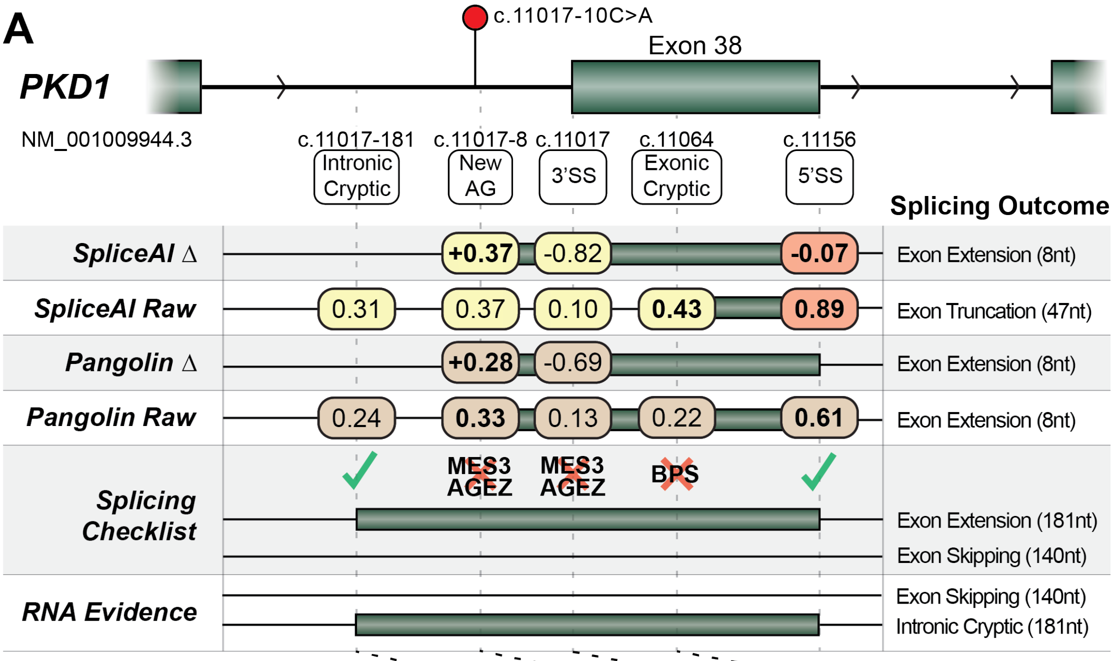


## B Splicing Checklist

| Metric | Motif Region | Value | Intronic Cryptic | New AG | Original 3’SS | Exonic Cryptic |
|---|---|---|---|---|---|---|
| 5’SS MaxEntScan | Upstream Donor | ≥ 1.45 | 8.19 | 8.19 | 8.19 | 8.19 |
| 3’SS MaxEntScan | AG motif | ≥ 1.38 | 13.02 | -2.62 | -2.06 | 2.29 |
| Total MaxEntScan | (Above) | ≥ 7.41 | 21.21 | 5.57 | 6.13 | 10.48 |
| Number Ts and Cs | c.x-24 to c.x-5 | ≥ 9 | 12 | 10 | 12 | 11 |
| Additional AG | c.x-13 to c.x-6 | = 0 | 0 | 1 | 1 | 0 |
| Intron length | Intron | ≥ 80 | 269 | 442 | 450 | 497 |
| * Branchpoint to AG | c.x-52 to c.x-15 | ≥ 17 | 35 | 17 | 25 | 14 |

## C Checklist Evaluation

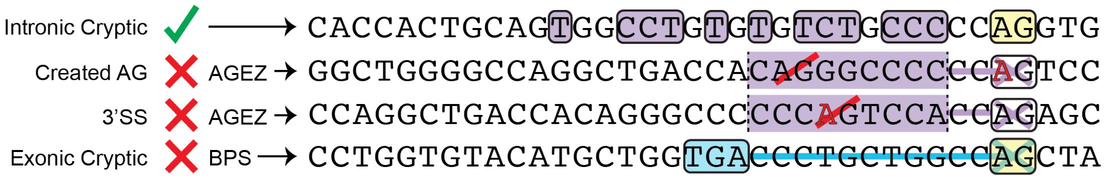


**Figure S1: *In silico* prediction and heuristics comparison for a variant in *PKD1*. A)** Lollipop plot of *PKD1* (NM_001009944.3) showing the transcript with the locations of the variants (not to scale). Dotted lines connect to the existing (3’SS and 5’SS) and proposed locations of splice sites, labeled with the exon’s first base (last base for 5’SS). Predictions are listed for the *PKD1*:c.11017-10C>A variant, using SpliceAI, Pangolin, our heuristics, and RNA Evidence (RT-PCR)[1]. Scores are color-coded based on the score class given (yellow: acceptor splice site; orange: donor splice site; brown: unspecified). The column on the right depicts the proposed splice site utilized as inferred from the scores. Δ: maximum score difference between the variant and wildtype. **B)** Heuristics-based interpretation of the proposed 3’SS locations. Values that do not meet the requirements are marked with a cross. **C)** Box colors correspond to the motif they represent (purple: polypyrimidine tract; yellow: acceptor motif; blue: branchpoint), white boxes at the canonical acceptor dinucleotides indicate the threshold for MaxEntScan 3’SS was not met. *: Not explicitly a requirement due to the difficulty of rule application.

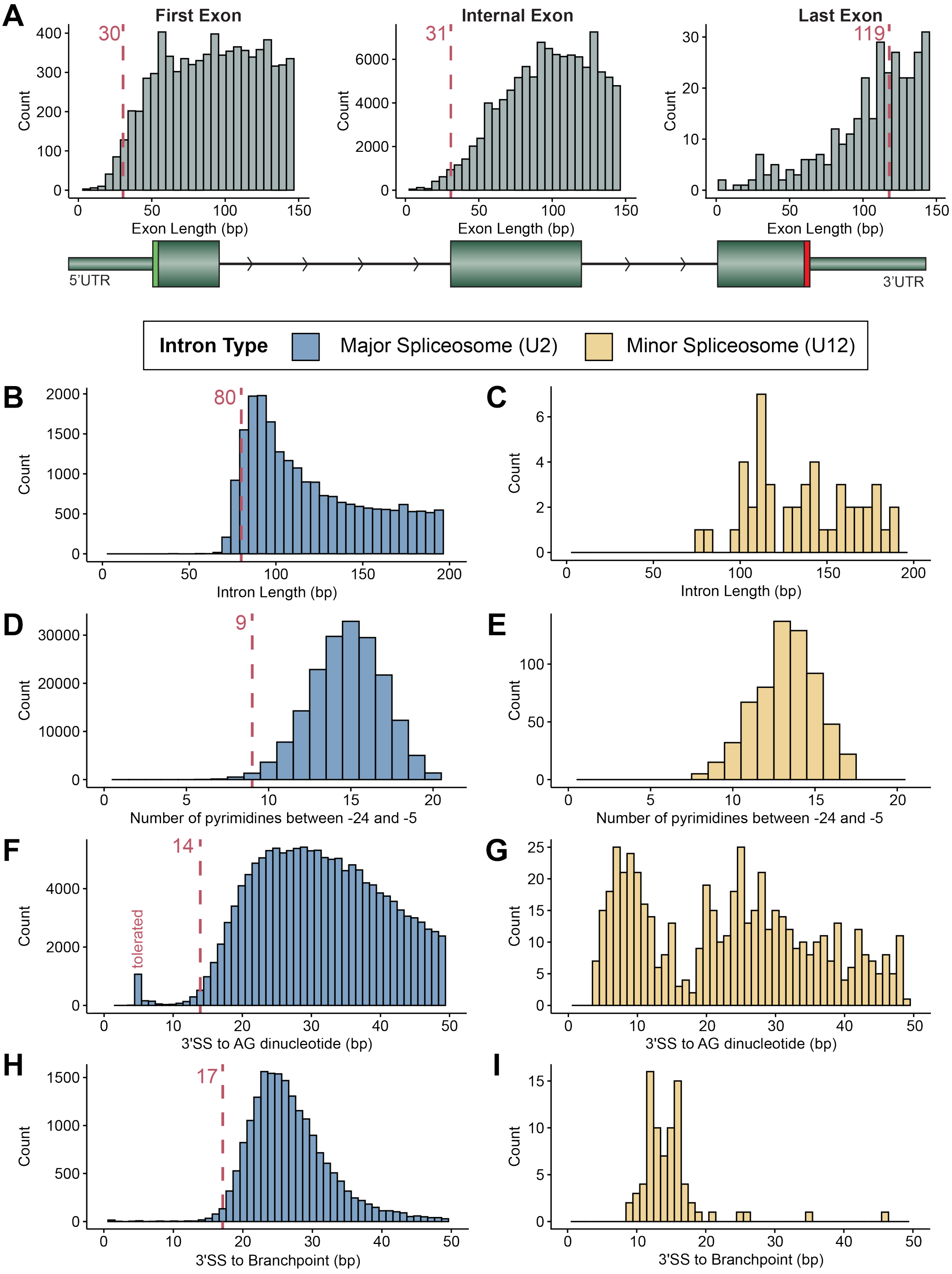


**Figure S2: Characteristics of splicing features.** Red lines show the value at which 99% of the U2 introns (left) are of equal value or greater. Characteristics are additionally shown for U12 introns (right) to show the difference in requirements for U2 and U12 introns. **A)** Minimum exon lengths for the first exon, internal exon (i.e., any exon not first or last), and last exon. **B-C)** Minimum intron lengths. **D-E)** Number of polypyrimidines (`T`s or `C`s) in the designated Polypyrimidine Tract Region ($-24$ to $-5$). **F-G)** Location of the closest `AG` dinucleotide to the 3′SS. `AG` dinucleotides occurring at the $-4$ location upstream of the 3′SS are tolerated. **H-I)** Positional requirement for the branchpoint motif in relation to the 3′SS. UTR: Untranslated Region. 3′SS: Acceptor Splice Site.

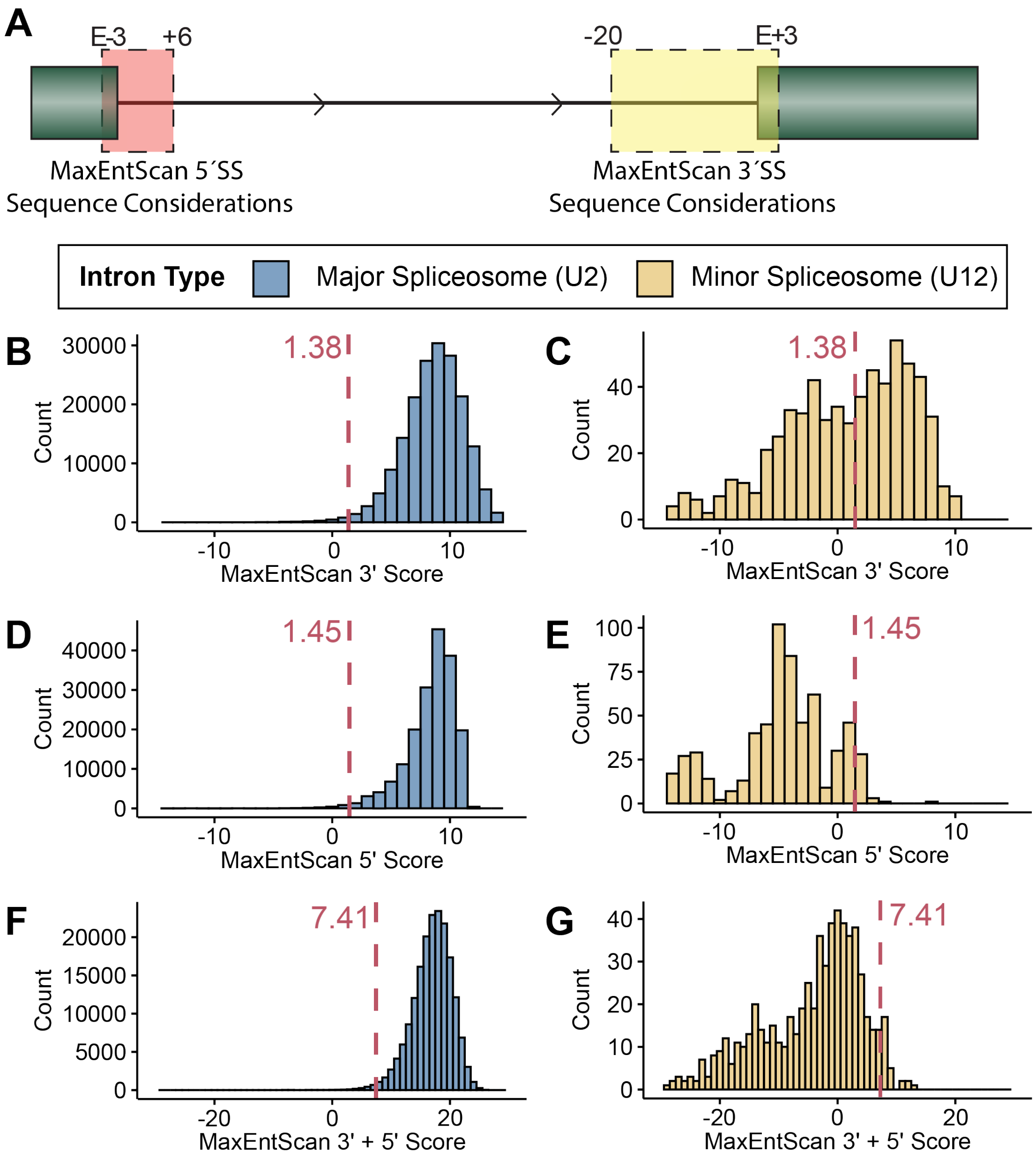


**Figure S3: Splicing *In Silico* Score Distributions for the U2 Checklist. A)** Green-filled boxes represent exons, and black lines with arrows represent the introns and the direction (5′ to 3′). The regions highlighted represent the sequence considered by MaxEntScan for 5′SS and 3′SS strength. **B-E)** Red lines show the value at which 99% of the U2 introns (left) are of equal value or greater. Score distributions are additionally shown for U12 introns (right) to show the limited applicability of the U2 requirements for U12 introns. **B-C)** MaxEntScan 3′ score distribution at the acceptor splice site. **D-E)** MaxEntScan 5′ score distribution at the donor splice site. **F-G)** MaxEntScan 3′ + 5′ score distribution at the upstream donor and acceptor splice sites (spanning the intron).

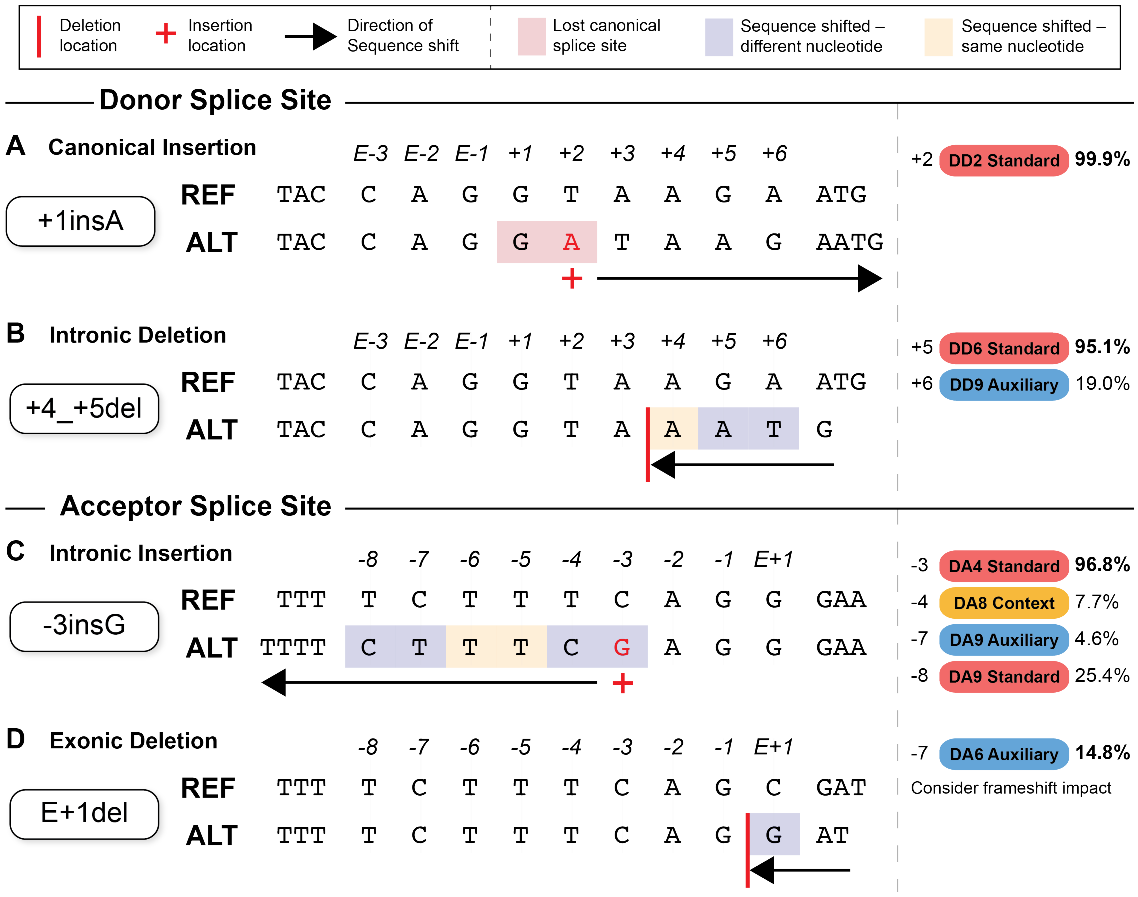


**Figure S4: Application of the heuristics to small insertions and deletions.** Splicing heuristics can be applied to small insertions and deletions by comparing the reference (ref) and variant (alt) sequences. The heuristics are applied to the nucleotides that differ between these sequences, with the highest spliceogenicity value assigned to the short insertion or deletion (indicated in bold).

# References


[1] Hort, Y., Sullivan, P., Wedd, L., Fowles, L., Stevanovski, I., Deveson, I., Simons, C., Mallett, A., Patel, C., Furlong, T. *et al.* (2023). Atypical splicing variants in PKD1 explain most undiagnosed typical familial ADPKD. npj Genomic Medicine *8*. doi:10.1038/s41525-023-00362-z.
